# Topical Analgesic Therapies for Acute, Sports‐Associated Soft Tissue Injuries

**DOI:** 10.1155/tsm2/9124204

**Published:** 2026-09-01

**Authors:** Mitchell S. Howard, Wade Lee-Smith, Justin P. Reinert

**Affiliations:** ^1^ Department of Pharmacy Practice, The University of Toledo College of Pharmacy and Pharmaceutical Sciences, Toledo, Ohio, USA; ^2^ University Libraries, The University of Toledo, Toledo, Ohio, USA, utoledo.edu

## Abstract

Acute musculoskeletal injuries are common among sports participants and are frequently treated with topical analgesics; however, the comparative efficacy and safety of topical formulations remain under investigation. The objective of this study was to evaluate the efficacy and safety of topical nonsteroidal anti‐inflammatory drugs (NSAIDs) and alternative topical therapies in the management of acute sports‐associated soft tissue injuries, including sprains, strains, contusions, and soreness. A systematic review and meta‐analysis were conducted following Preferred Reporting Items for Systematic Reviews and Meta‐Analyses (PRISMA) criteria, including randomized controlled trials that evaluated topical NSAIDs or other topical analgesics in patients with acute sports‐related musculoskeletal injuries. Bias was assessed with the RoB‐2 tool. The primary efficacy outcome was reduction in pain, measured by the visual analog scale (VAS) or similar validated tools, while the primary safety outcome was the incidence of treatment‐emergent adverse effects. Eight randomized controlled trials comprising 1027 patient encounters met inclusion criteria. Across studies, topical diclofenac, ibuprofen, ketoprofen, and etofenamate demonstrated statistically significant reductions in VAS pain scores compared with placebo. Meta‐analysis of all included trials revealed no significant reduction in pain intensity in active topical interventions versus placebo (95% CI −0.3768–5.2806; *p* = 0.09). A direct comparative analysis of diclofenac versus other NSAIDs did not demonstrate a statistically significant difference (effect size −2.07; 95% CI −5.60–1.46; *p* = 0.25), though heterogeneity of studies may limit these findings. Adverse effects were primarily mild and localized and were no different between groups (RR = 0.79; 95% CI 0.45–1.39; *p* = 0.41). Topical NSAIDs and alternative topical formulations may provide improved pain outcomes but did not show superiority between NSAID agents.

## 1. Introduction

Sports‐associated injuries are common in both professional and amateur athletes, with a postulated injury incident rate ranging from 65/1000 h of sport in track and field to approximately 4/1000 h in baseball and softball [1]. Even when reviewed in adolescent athletes, the injury incident rate is significant in soccer (7.21/1000 h), of which musculoskeletal muscle strains were the most commonly reported injury at 12.24% [2].

Musculoskeletal damage, including muscle sprains, strains, contusions, and over‐exertion, is the most commonly diagnosed sports‐associated soft tissue injury across the globe [3]. While the impact on the performance of elite, professional athletes due to injury is well documented across physical, emotional, and financial domains, the ramifications for the recreational population are less well defined [4]. Sports‐associated soft tissue injuries appear to be more common in adults as opposed to adolescents and younger people and present with a higher incidence in women than in men [5]. While clinical practice consensus statements have been published regarding treatment modalities for such injuries, the World Anti‐Doping Agency’s Therapeutic Use Exemption Physician Guidelines for Pain Management in Sport specifically endorse nonpharmacological measures as the first‐line treatment for acute pain, with non‐steroidal anti‐inflammatory drugs (NSAIDs) or other non‐narcotic analgesics being considered an appropriate first‐line pharmacological therapy in chronic pain to be used as needed [6, 7].

As postulated by Dubois and colleagues, the current rehabilitation strategy for sports‐associated soft tissue injuries relies upon deployment of the PEACE and LOVE strategies, meaning protection, elevation, avoidance of anti‐inflammatory medications, compression, education, love, optimism, vascularization, and exercise [8]. The position to avoid anti‐inflammatory medications is discussed by Fowler et al., who summarize earlier evidence that NSAIDs as well as ice and cooling agents may be associated with impaired tissue healing, angiogenesis, and revascularization [9]. The regular use of topical NSAIDs by athletes raises an important discrepancy between evidence‐based practice and historic use of the products. Despite this proposition, several NSAID products have been specifically formulated and marketed toward sports or exercise‐induced injury, especially topical pharmacologic products [10].

Several NSAIDs are available in either over‐the‐counter or prescription‐strength topical formulations, including diclofenac, ibuprofen, and ketorolac. Though subtle differences exist related to their respective potencies and drug attributes, all provide anti‐inflammatory effects due to their mechanism of inhibiting cyclooxygenase (COX) enzymatic activity, which in turn drastically decreases the production of pro‐inflammatory prostaglandins [11]. Systemic absorption of topical NSAID products is considered to be minimal, affecting primarily the outer layers of skin and the dermis (∼3–4 mm deep), and noncontributory to efficacy in localized injuries. Adverse effects associated with oral or intravenous administration of these medications, including acute kidney injury and gastrointestinal hemorrhage, are rare and not of overt clinical concern [12, 13].

Given the ambiguity of their recommended use in practice and the differences between drug products and topical formulations, the objective of this systematic review and meta‐analysis was to determine the efficacy and safety of topical analgesic therapies for acute, sports‐associated soft tissue injuries.

## 2. Methods

A systematic review and meta‐analysis was conducted following the Preferred Reporting Items for Systematic Reviews and Meta‐Analyses (PRISMA) guidelines [14]. This study protocol was registered with the International Platform of Registered Systematic Review and Meta‐Analysis Protocols (protocol number: INPLASY202670096). All study authors contributed equally to the study design, data extraction, synthesis, and manuscript preparation. Ethics approval and informed consent were not necessary for this systematic review.

**FIGURE 1:**
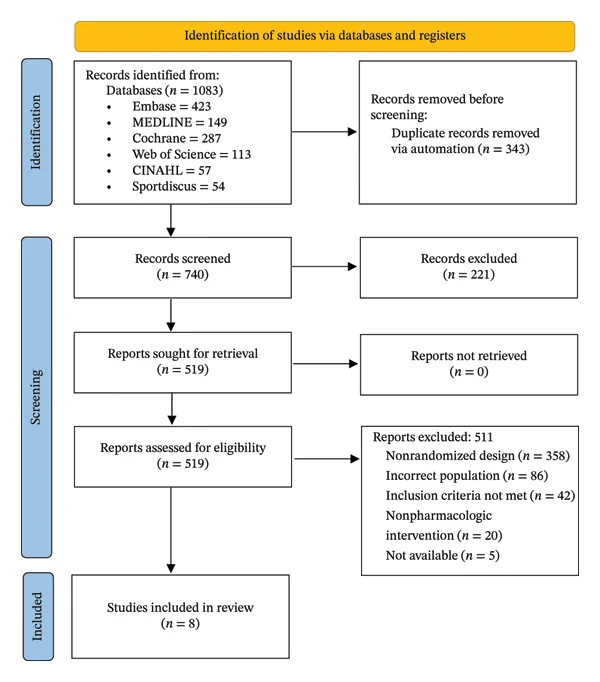
PRISMA diagram [9].

A comprehensive search strategy to identify reports of topical analgesia used to treat sports‐related injuries was constructed in Embase (Embase.com, Elsevier) by an experienced health sciences librarian (Wade Lee‐Smith) using truncated keywords, phrases, and subject headings and subheadings. Searches for common sports injuries, specific sports, and general terms relating to sport medicine were combined with terms for analgesics as a drug classification, specific analgesics, and analgesia in general and then further limited to topical or transdermal administration or dosage forms. This strategy was translated to MEDLINE (OVID platform), Cochrane Central Register of Controlled Trials (CochraneLibrary.com, Wiley), the Web of Science Core Collection (Web of Science platform, Clarivate), CINAHL, and SPORTDiscus (EBSCO Research Database platform), with all searches performed on 30 May 2025. The Supporting Section Table 1 provides details for the full search strategies. No publication date or language limits were used; however, animal studies, previous evidence syntheses, reviews, and case reports were excluded whenever possible using database indexing. All results were exported to EndNote 21 citation management software (Clarivate, Philadelphia, PA, USA), and duplicates were removed by successive iterations of EndNote’s duplicate detection algorithms and manual inspection. These deduplicated results were then exported via RIS file for screening in Lean Library Workspace (Technology from Sage, Thousand Oaks, CA, USA). A PRISMA flow diagram of our search strategy can be viewed in (Figure 1). Randomized controlled trials that provided complete efficacy and safety outcomes of topical analgesics in sports or exercise‐induced soft tissue injuries in adult patients were eligible for inclusion. The use of concomitant, nonpharmacological therapies was considered eligible for inclusion based on the primary studies’ protocols. Time‐to‐intervention was not a controlled parameter for study inclusion, and primary literature was considered eligible for inclusion so long as patients within the original studies followed the protocol within those studies. Non‐randomized trials, those utilizing combination therapies of multiple pharmaceutical agents concomitantly, those assessing other types of injuries in athletes not specifically identified as a soft tissue injury, and those not available or readily translatable to English were excluded. No modifications to inclusion and exclusion criteria were made during the screening and data extraction processes. Priority for data extraction was given to those studies that clearly documented patient outcomes related to improved pain symptoms and reductions in pain scores. Two authors were responsible for screening and deciding upon inclusion or exclusion of studies (Mitchell S. Howard and Justin P. Reinert), with any unresolved discrepancies being forwarded to the third author for adjudication (Wade Lee‐Smith). Automation was utilized to remove duplicate entries in Lean Library Workspace and confirmed for accuracy by the authors (Mitchell S. Howard and Justin P. Reinert). Data extraction from included studies was completed by two authors independently and then reviewed collectively for precision. The Risk of Bias in Randomized Trials (ROB‐2) was completed by two authors (Mitchell S. Howard and Justin P. Reinert), with any conflicting results being referred to a third party for adjudication (Wade Lee‐Smith). The results of the ROB‐2 assessment can be found in Table 1 https://sciwheel.com/work/citation?ids=7468130%26pre=%26suf=%26sa=0%26dbf=0 [15].

**TABLE 1:** Risk of bias assessment [10].

	Risk of bias arising from the randomization process	Risk of bias due to deviations from the intended interventions (effect of assignment to intervention)	Risk of bias due to deviations from the intended interventions (effect of adhering to intervention)	Missing outcome data	Risk of bias in measurement of the outcome	Risk of bias in selection of the reported result	Overall risk of bias
Galer et al. [13]	Low	Low	Low	Low	Moderate	Low	Low
Airaksinen et al. [14]	Low	Low	Moderate	Low	High	Low	Moderate
Cannavino et al. [15]	Low	Low	Low	Low	Moderate	Low	Low
Lionberger et al. [16]	Low	Low	Low	Low	Moderate	Low	Low
Predel et al. 2016 [17]	Low	Low	Low	Low	Moderate	Low	Low
Predel et al. [18]	Low	Low	Low	Low	High	Low	Moderate
Predel et al. [19]	Low	Low	Low	Low	High	Moderate	Moderate
Predel et al. [20]	Low	Low	Low	Low	High	Low	Moderate

A meta‐analysis was conducted on Meta‐Mar (v4.0.2) [16]. Standard mean difference was utilized to compare analgesic efficacy outcomes when heterogeneity existed regarding pain assessment variables. A random effects model was utilized for a comparison of active interventions compared to placebo and for diclofenac in comparison to other NSAID preparations. A risk ratio was calculated for safety outcomes across all studies in aggregate. The threshold for statistical significance was set at an alpha value of < 0.05. Heterogeneity of included studies was evaluated using *I* ^2^ values.

## 3. Results

### 3.1. Systematic Review

After the initial screening following the inclusion and exclusion criteria, 8 randomized controlled trials were identified and assessed in this study [17–24]. The results are provided in Table 2. These studies included 1039 patients receiving either active pharmaceutical intervention (*n* = 519) or placebo (*n* = 520). A total of 1027 patients were included in the meta‐analysis of NSAID‐related outcomes. Agents were applied as patches, plasters, gel, or cream.

**TABLE 2:** Results.

Author/year	Mechanism of injury	Participants	Intervention	Comparator	Efficacy	Adverse effects	Additional information
Galer et al., 2000 [13]	Sprains, strains, and contusions	213 patients; naturally occurring sports injury within 72 h of enrollment	Diclofenac epolamine 1.3% patch (*n* = 106)	Placebo (*n* = 107)	Mean VAS reduction favored diclofenac at Day 14 (4.7 vs. 4.2 mm, *p* < 0.044)	3/106 patients discontinued diclofenac due to a skin rash (*n* = 1), worsening pain (*n* = 1), and a skin infection (*n* = 1). No statistically significant differences were observed between groups	Baseline mean VAS = 6.41 ± 1.08

Airaksinen et al., 2003 [14]	Soft tissue injury of ankle, leg, knee, hand	74 patients; naturally occurring minor soft tissue injury of extremities < 48 h before exam	Ice Power Cold Gel 5 g AAA QID (Ethanol 8% & Menthol 3.5%) (*n* = 37)	Placebo Gel 5 g AAA QID (*n* = 37)	Mean VAS for pain at rest favored Ice Power (45 vs. 33 mm, *p* < 0.0001) POM during normal fxn VAS Decreased CG 51 vs. Placebo 46 *p* < 0.001 Fxn disability VAS Decreased each week with both treatments. Week 1: 32 vs. 14 Differences *p* < 0.001 Secondary: Patient Global Assessment and Satisfaction (Score 0–3) (2.3 0.3 for cold gel vs. 1.4 0.3 for placebo, *p* < 0.001) Investigators′ Global Assessment and Satisfaction (2.4 0.3 for cold gel versus 1.5 0.3 for placebo, *p* < 0.001)	2 patients in each group reported minor skin abrasions after application. No patients discontinued the study. No other ADR reported	Additional reductions of efficacy markers at each check NSAIDs allowed in both groups as rescue med (recorded in Table 3–cold 6 vs. Placebo 9) Knee/Ankle injuries provided elastic bandages × 14 days

Cannavino et al., 2003 [15]	Induced muscle weakness and soreness	32 patients; induced exercise	Ketoprofen 10% topical cream (*n* = 16)	Placebo (*n* = 16)	Reduction of DOMS scores in legs favored ketoprofen through 48 h (4.56 vs. 2.88 DOMS score, *p* = 0.002)	No adverse effects reported.	Reviewed serum ketoprofen levels were evaluated and reported to be minimal and not clinically significant

Lionberger et al., 2011 [16]	Ankle sprains	134 patients with naturally occurring sports injury within 28 h of enrollment	Diclofenac epolamine 1.3% patch (*n* = 68)	Placebo (*n* = 66)	Mean reduction in VAS at day 7 demonstrated a statistically significant difference in favor of diclofenac (56.4 mm vs. 51.6 mm, *p* = 0.0008). Investigator‐specified efficacy endpoint of 30% reduction in baseline pain favored diclofenac (36.8 vs. 16.7% of patients, *p* = 0.01)	Localized adverse effects occurred in 3% of the diclofenac group vs. 4.5% of the placebo group. No serious effects were reported in either group	Approximately 40% of participants used ice, equally split between cohorts

Predel et al., 2016 [17]	Impact soft tissue injuries, including sprains, strains, and contusions to extremities	168 patients with naturally occurring sports injuries within 3 h prior to randomization	Diclofenac sodium 140 mg patch (*n* = 84)	Placebo (*n* = 84)	Mean reduction in VAS = −42.34 ± 20.69 mm for diclofenac vs. −18.09 ± 15.11 mm for placebo (*p* < 0.001). Mean reduction of VAS at day 7 was also statistically significant (−43.17 ± 18.77 mm for diclofenac vs. −33.18 ± 17.34 mm for placebo, *p* < 0.001)	Adverse effects were reported in 6% of the diclofenac group vs. 8.3% in the placebo group. All described localized adverse effects. No serious effects occurred in either group	Ice/cooling spray permitted prior to randomization; tepid water and RICE permitted throughout the study; acetaminophen permitted as rescue medication throughout study

Predel et al., 2017 [18]	Traumatic blunt soft tissue injury or contusion to extremities	132 patients with naturally occurring sports injuries within 3 h of enrollment	Ibuprofen medicated plaster (*n* = 64)	Placebo (*n* = 68)	Ibuprofen was associated with a statistically significant reduction of POM VAS at 72 h (−65.2 mm vs. −38.8 mm, *p* < 0.0001). Reduction in POM was also favorably reduced with ibuprofen at hours 12, 24, 28, and 120 (*p* < 0.0001 for each)	Localized adverse effects occurred in 1.6% of the ibuprofen group vs. 8.8% of the placebo group. No serious adverse effects were reported	RICE permitted prior to randomization

Predel et al., 2018 [19]	Acute sports‐related blunt soft tissue injury or contusion	130 patients; naturally occurring sports injury within 3 h of enrollment	Ibuprofen 200 mg plaster (*n* = 66)	Placebo (*n* = 64)	VAS AUC was statistically significantly reduced, favoring ibuprofen through 72 h (61.71 vs. 47.48 mm, *p* = 0.001)	7 patients in the ibuprofen cohort and 8 patients in the placebo cohort experienced mild, localized adverse effects. No serious adverse effects were reported	SSRI/SNRI medications are permitted if a home medication and dose was not titrated within 4 weeks of randomization

Predel et al., 2021 [20]	Ankle sprain	156 patients; Naturally occurring sports injury	Etofenamate 70 mg plaster (*n* = 78)	Placebo (*n* = 78)	Reduction in VAS score from baseline to 72 h was statistically significant in favor of etofenamate (40.55 vs.18.12 mm reduction, *p* < 0.0001). POM improved more in the etofenamate cohort through 168 h (mean difference 19.1 mm, *p* < 0.0001)	2 patients in the etofenamate group and 3 patients in the placebo group experienced mild, localized reactions. No serious adverse effects were reported	NNT for VAS reduction at 72 h = 3

*Note:* Legend: AUC, area under the curve.

Abbreviations: DOMS, delayed onset muscle soreness; POM, pain on movement; RICE, rest, ice, compression, elevation; ROM, range of motion; SNRI, selective norepinephrine reuptake inhibitor; SPID, summed pain intensity difference; SSRI, selective serotonin reuptake inhibitor; VAS, visual analog scale.

#### 3.1.1. Pain Reduction

All patients receiving active intervention demonstrated statistically significant improvements in pain scores when compared to placebo. This also applied to one study that reported delayed onset muscle soreness (DOMS) scores, where ketoprofen 10% topical cream was found to be more efficacious than placebo. While most studies evaluated time points within the 5–14 days post‐dose period for considerations of efficacy, the studies conducted by Cannavino et al., Predel et al. (2018), and Predel et al. (2021) demonstrated sustained efficacy and statistical significance of the benefit of interventions for periods of 24–72 h [14, 18, 19].

#### 3.1.2. Adverse Effects

In one study, 3 patients discontinued treatment due to skin rash, worsening pain, or skin infection [12]. In 6 studies, 19 patients receiving the intervention and 29 patients receiving placebo reported minor localized adverse effects but did not discontinue treatment. [12, 15–19]. One study reported no adverse effects throughout their trial [14]. All studies reported no statistically significant difference in the safety profile of the interventions versus the placebos.

### 3.2. Meta‐Analysis

**FIGURE 2:**
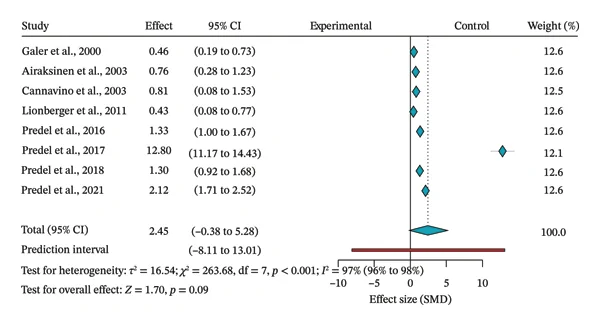
Reduction in average pain scores between active interventions and placebo.

**FIGURE 3:**
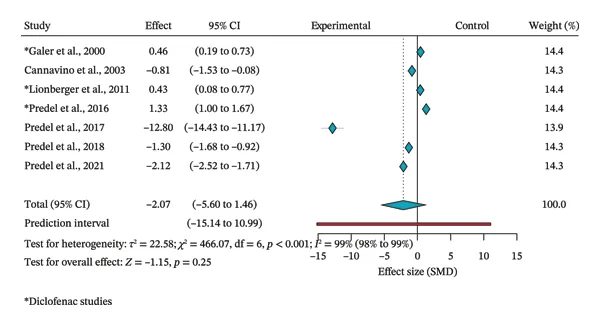
Comparative efficacy of diclofenac vs. other NSAIDS. ^∗^Diclofenac studies.

**FIGURE 4:**
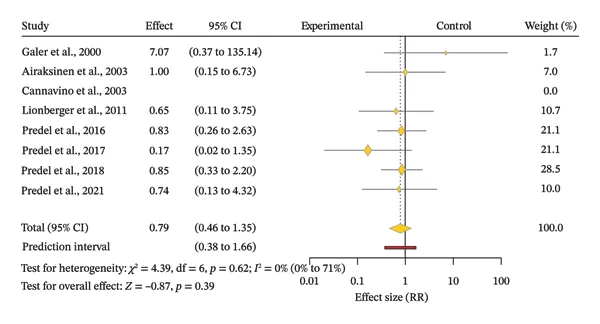
Adverse effects between active intervention and placebo.

Regarding the primary outcome, 1027 patient encounters were analyzed using a random‐effects model that produced a pooled standardized mean difference (SMD) of 2.4519 with a 95% confidence interval of −0.3768 to 5.2806 [17–24]. The Z‐value was 1.6989, and the *p* value was 0.09, indicating that the effect was not statistically significant (Figure 2). When comparing diclofenac‐containing products (*n* = 3 studies) to other NSAID‐containing products, including ibuprofen (*n* = 3 studies) and ketoprofen (*n* = 1 study), the random‐effects model reported a pooled effect size of Standardized Mean Difference (SMD) = −2.0719 with a 95% CI of (−5.6031, 1.4592). The Z‐value was −1.15, with a *p* value of 0.25, indicating no statistically significant effect (Figure 3) [17, 19–24]. Regarding safety outcomes, a binary effects model was used to quantify the incidence of adverse effects among all included studies (Figure 4) [17–24]. No difference was identified between active pharmaceutical preparations and placebo, with a risk ratio of 0.79 (95% CI = 0.45–1.39, *p* = 0.41). Heterogeneity of studies exceeded a 40% threshold in this analysis, indicating that baseline populations and comparative outcome measures represented a broad scope.

## 4. Discussion

The provision of active pharmaceutical products in formulations did not demonstrate more benefit than placebo across all domains, and only minor, if any, safety concerns were reported in the studies reviewed in this work. Despite this, there are several findings of this systematic review and meta‐analysis that warrant discussion.

The evaluation scales of pain used by trials included in this study introduced significant heterogeneity; however, it is important to note that universal improvement was noted in cohorts receiving active ingredients [25, 26]. The visual analog scale (VAS) was the most commonly adopted metric across all studies, though it was applied to different domains and types of painful presentation that may be encountered by athletes [27]. Evaluating pain upon movement (POM) may have different clinical significance than in the prevention or treatment of DOMS, and each patient should be managed according to their presentation and with specific goals of care [28, 29]. Interestingly, only one of the studies included in this review provided their definition of a statistically significant reduction in the VAS score of 30% from baseline, whereas others seemingly compared the post‐intervention averages between two groups [30]. This 30% threshold aligns with previous conclusions reached in acute care settings, where a reduction of 30 mm on a 100 mm VAS tape was found to correlate with patients’ expectancy of adequate pain control. Despite this, it is also relevant to consider that reductions of 13 mm were found to be noticeable by patients and clinically significant in the same study [30]. When applying these findings to our work, it should be noted that many of the included studies did not reach the threshold of a 13 mm reduction between intervention and placebo. One potential explanation for this may be that, across all included studies, the initial pain encountered was relatively low on the VAS scale when compared to other types of acute pain encountered, such as in emergency medical settings.

This analysis also commands consideration of the variation of drug formulations applied. Topical dosage forms differ in their physicochemical properties and structural organization, which directly influence drug release from the vehicle, partitioning into the stratum corneum, and subsequent percutaneous absorption [31]. These formulation‐dependent factors are central determinants of local bioavailability, onset and duration of action, and systemic exposure. Ointments are hydrophobic, largely anhydrous semisolid systems that exert a pronounced occlusive effect, increasing stratum corneum hydration and drug residence time at the application site [32]. This occlusion can enhance penetration of certain active pharmaceutical ingredients, although it may also limit patient acceptability [32]. Creams are emulsion‐based systems with lower occlusivity, and drug delivery from creams is governed by emulsion type, excipient selection, and phase–skin interactions [33]. Gels, which are commonly aqueous or hydroalcoholic polymeric systems, often facilitate more efficient drug release from the vehicle and uniform skin contact. Comparative studies have demonstrated higher permeation from gel formulations than from creams for selected drugs, indicating that relative bioavailability cannot be inferred solely from dosage form category [34]. Spray formulations, particularly film‐forming systems, deliver drug as a thin layer that rapidly dries to form a residual film on the skin, with absorption characteristics influenced by solvent evaporation, film integrity, and drug concentration within the deposited matrix [35]. Medicated plasters and topical patch systems are designed to provide prolonged skin contact and controlled release, resulting in sustained local drug exposure [36]. For topical nonsteroidal anti‐inflammatory drugs, these systems have been shown to achieve therapeutically relevant tissue concentrations with minimal systemic exposure relative to oral administration, while also demonstrating formulation‐specific differences in absorption kinetics and overall exposure when compared with gels and sprays [37].

The findings of the present systematic review and meta‐analysis expand upon prior evidence regarding topical analgesic therapies for acute musculoskeletal injuries. In the Cochrane review by Derry et al., topical NSAIDs were shown to provide clinically meaningful analgesia for acute musculoskeletal pain with favorable tolerability, particularly diclofenac and ketoprofen preparations, while demonstrating lower systemic adverse event risk compared with oral NSAIDs [38]. Similarly, Nudo et al. specifically evaluated injured athletic populations and concluded that topical analgesics were generally comparable to oral therapies for pain relief while maintaining a superior safety profile, although significant heterogeneity and variability in formulations limited definitive recommendations [39]. The present study builds upon these earlier analyses by narrowing the focus specifically to acute, sports‐associated soft tissue injuries and by comparatively evaluating multiple topical NSAID formulations to nonactive pharmacotherapeutic alternatives and delivery systems, including plasters, patches, creams, and gels, within a sports medicine framework. Crucially, and in contrast to the reviews conducted previously, our work showed that there was no difference between active pharmacologic products and placebo, raising the question as to whether topical medications are necessary in these types of injuries.

One important consideration for clinicians managing athletic injuries is the balance between analgesia, inflammation, and tissue healing. The therapeutic effect of topical NSAIDs is mediated through inhibition of COX‐1 and COX‐2 enzymes, reducing local prostaglandin synthesis responsible for pain sensitization, vasodilation, and inflammatory signaling [11]. Although attenuation of excessive inflammation may improve comfort and facilitate earlier participation in rehabilitation, the acute inflammatory response also represents a component of normal tissue repair, promoting angiogenesis, cellular recruitment, extracellular matrix remodeling, and subsequent regeneration of injured muscle, tendon, and ligament [3, 9]. This physiological relationship has prompted contemporary sports medicine recommendations to discourage the routine suppression of inflammation immediately following injury, emphasizing progressive loading and rehabilitation over aggressive pharmacologic intervention [8]. The findings of the present review support this evolving paradigm by demonstrating that topical NSAIDs provide a favorable safety profile with predominantly mild, localized adverse effects while failing to demonstrate clear superiority over placebo in pooled analyses, suggesting that improvements in athlete recovery may be driven as much by the natural healing process and structured rehabilitation as by pharmacologic intervention itself [12, 13]. From a practical sports medicine perspective, topical NSAIDs may be most appropriately considered as adjunctive therapies intended to improve short‐term symptom control and permit participation in evidence‐based rehabilitation rather than as interventions that accelerate biologic tissue healing. This distinction is particularly important for athletes and clinicians seeking to optimize return‐to‐play decisions while minimizing unnecessary pharmacologic exposure and preserving the normal physiologic processes required for complete musculoskeletal recovery [6–10].

### 4.1. Limitations

This study has several limitations that should be considered when interpreting the findings. Although a systematic screening process was used, there remains the possibility that relevant articles may have been inadvertently omitted during the screening process, which could have introduced selection bias. Additionally, the number of articles included in the analysis was relatively small (*n* = 8), along with the number of participants in each study, limiting the robustness and generalizability of the findings. Furthermore, participant characteristics, including age and physical fitness levels, while not explicitly stated in the demographics, could have varied across studies and may have influenced injury risk, recovery, and reported efficacy or safety outcomes. One authorship group was responsible for publishing four of the included studies, though no evidence of overlapping patient cohorts or interventions was able to be determined.

Heterogeneity among the included studies represents another limitation. One study evaluated experimentally induced muscle pain or soreness before treatment [19], whereas the remaining studies examined naturally occurring injuries and follow‐up care, which may limit comparability of the DOMS and VAS outcomes.

Finally, not all products evaluated in the included studies are commercially available. For example, some studies examined the use of topical plasters, which may require specialized formulation through compounding pharmacies rather than being readily accessible as commercially marketed products. Some patients may also attempt to treat their soft‐tissue injury through self‐care. Products may vary in their status as a prescription‐only medication vs. an over‐the‐counter product. This limitation may affect the applicability and real‐world implementation of the findings, with treatment in these studies occurring in < 3–72 h.

## 5. Conclusion

This systematic review and meta‐analysis demonstrated that topical NSAID formulations did not provide statistically significant analgesic benefit compared with placebo for acute, sports‐associated soft tissue injuries, though they retained a favorable safety profile characterized primarily by mild, localized adverse effects. Despite the lack of statistical significance, active pharmacologic therapy consistently improved pain outcomes, with no clear evidence of superiority among individual NSAID agents. Future high‐quality trials with standardized outcome measures, longer follow‐up, and direct head‐to‐head comparisons of topical dosage forms are warranted to better define optimal use strategies and to clarify the relationship between formulation‐dependent pharmacokinetics and clinically meaningful outcomes in sports‐related soft tissue injury management.

## Funding

No funding was received for this manuscript.

## Disclosure

This work was previously presented as a poster and abstract supplement as “Topical Analgesic Therapies for Acute, Sports‐Associated Soft Tissue Injuries: A Systematic Review and Meta‐Analysis” at The American Pharmacists Association Annual Meeting in March 2026 and July 2026, respectively [40].

## Conflicts of Interest

The authors declare no conflicts of interest.

## Supporting Information

Additional supporting information can be found online in the Supporting Information section.

## Supporting information


**Supporting Information 1** The supporting section includes a table that describes the full search strategies for identifying potential articles for this manuscript.

**Supporting Information 2** PRISMA_2020_checklist_Topical STI Review.

## Data Availability

Data sharing is not applicable to this article as no datasets were generated or analyzed during the current study.
