# Supplementary material for: Topical Analgesic Therapies for Acute, Sports‐Associated Soft Tissue Injuries
Source: Transl Sports Med. 2026 Sep 1;2026:9124204. doi: 10.1155/tsm2/9124204 (PMC13535159; doi:10.1155/tsm2/9124204)
Supplement: Supplementary file 2 [file TSM2-2026-9124204-s002.docx]

**Supplementary Section Table 1: Full Search Strategies**

Embase (Embase.com, Elsevier)

| No. | Embase Query | Results |
| --- | --- | --- |
| #1 | sport medicine':ti,ab,kw OR 'sport physiology':ti,ab,kw OR 'sports physiology':ti,ab,kw OR 'sports medicine':ti,ab,kw OR 'rehabilitation science':ti,ab,kw OR 'rehabilitation medicine':ti,ab,kw OR 'sports medicine'/exp OR 'rehabilitation medicine'/exp | 53173 |
| #2 | 'return to sport'/exp OR 'sport injury'/exp OR 'athlete injury':ti,ab,kw OR 'athlete trauma':ti,ab,kw OR 'athletic injuries':ti,ab,kw OR 'athletic injury':ti,ab,kw OR 'athletic trauma':ti,ab,kw OR 'injury, athletic':ti,ab,kw OR 'sport accident':ti,ab,kw OR 'sport related injury':ti,ab,kw OR 'sport trauma':ti,ab,kw OR 'sportrelated injury':ti,ab,kw OR 'sports injury':ti,ab,kw OR 'sports related injury':ti,ab,kw OR 'sports trauma':ti,ab,kw OR 'sportsrelated injury':ti,ab,kw OR 'sport injury':ti,ab,kw OR 're-sumption of sport':ti,ab,kw OR 'resumption of sporting activities':ti,ab,kw OR 'return to play (sport)':ti,ab,kw OR 'return to playing sport':ti,ab,kw OR 'return to sporting activities':ti,ab,kw OR 'return to sport':ti,ab,kw | 50114 |
| #3 | sport*:ti,ab,kw OR athlet*:ti,ab,kw OR 'sport'/de OR 'athletics'/exp OR 'ball sport'/exp OR 'body building'/exp OR 'calisthenics'/exp OR 'climbing sport'/exp OR 'collision sport'/exp OR 'combat sport'/exp OR 'contact sport'/exp OR 'cross training (sport)'/exp OR 'cycling'/exp OR 'disabled sport'/exp OR 'endurance sport'/exp OR 'extreme sport'/exp OR 'gymnastics'/exp OR 'horseback riding'/exp OR 'jogging'/exp OR 'motor sport'/exp OR 'non contact sport'/exp OR 'nordic walking'/exp OR 'racewalking'/exp OR 'racquet sport'/exp OR 'roller skating'/exp OR 'running'/exp OR 'skateboarding'/exp OR 'team sport'/exp OR 'triathlon'/exp OR 'water sport'/exp OR 'weight lifting'/exp OR 'winter sport'/exp OR 'youth sport'/exp OR 'athlete'/exp | 368552 |
| #4 | tennis elbow'/exp OR 'medial epicondylitis'/exp OR 'achilles tendinitis'/exp OR 'anterior cruciate ligament injury'/exp OR 'patellofemoral pain syndrome'/exp OR 'bursitis'/exp OR 'rotator cuff injury'/exp OR ('ligament injury'/exp AND 'ulnar collateral ligament'/exp) OR ('knee meniscus rupture'/exp NOT (eye OR ocular OR ophtalm*)) OR 'ankle sprain'/exp OR ('ligament injury'/exp AND 'ankle lateral ligament'/exp) OR ('hamstring muscle'/exp AND 'muscle injury'/exp) OR 'bursitis, radiohumeral' OR 'elbow, tennis' OR 'epicondylatgia humeri' OR 'epicondylitis humeri' OR 'epicondylitis lateralis' OR 'epicondylitis, external humeral' OR 'epicondylitis, radiohumeral' OR 'epycondylitis humeri' OR 'external humeral epicondylitis' OR 'humeral epicondylitis' OR 'humerus epicondylitis' OR 'lateral elbow tendinopathy' OR 'lateral elbow tendinosis' OR 'lateral elbow tendonopathy' OR 'lateral epicondylitis' OR 'lateral humeral epicondylitis' OR 'radiohumeral bursitis' OR 'radiohumeral epicondylitis' OR 'tennis arm' OR 'tennis elbow' OR 'epicondylitis medialis' OR 'golfer arm' OR 'golfer elbow' OR 'medial elbow tendinopathy' OR 'medial elbow tendinosis' OR 'medial humeral epicondylitis' OR 'medial epicondylitis' OR 'anterior cruciate ligament injuries' OR 'anterior cruciate ligament injury' OR 'anterior cruciate knee ligament rupture' OR 'rupture, anterior crucial ligament' OR 'anterior cruciate ligament rupture' OR 'achilles tendinopathy' OR 'achilles tendinosis' OR 'achilles tendonopathy' OR 'achilles tendinitis' OR 'patella femoral pain syndrome' OR 'patellar femoral pain syndrome' OR 'patello-femoral pain syndrome' OR 'patello-femoral stress syndrome' OR 'patello-femoral syndrome' OR 'patellofemoral stress syndrome' OR 'patellofemoral syndrome' OR 'patellofemoral pain syndrome' OR 'bursitis' OR 'calcaneal bursitis' OR 'capped hock' OR 'heel bursitis' OR 'retrocalcaneal bursitis' OR 'infection in the bursa' OR 'infectious bursitis (human)' OR 'infective bursitis' OR 'septic bursitis' OR 'shoulder bursitis' OR 'shoulder`s bursitis' OR 'subacromial-subdeltoid bursitis' OR 'subacromial/subdeltoid bursitis' OR 'subdeltoid bursitis' OR 'subacromial bursitis' OR 'rotator cuff impingement' OR 'rotator cuff syndrome' OR 'rotator cuff tendinitis' OR 'rotator cuff tendonitis' OR 'shoulder cuff syndrome' OR 'shoulder impingement' OR 'subacromial impingement' OR 'subacromial impingement syndrome' OR 'syndrome, shoulder impingement' OR 'shoulder impingement syndrome' OR 'cuff injury' OR 'cuff lesion' OR 'rotator cuff disease' OR 'rotator cuff disorder' OR 'rotator cuff disorders' OR 'rotator cuff injuries' OR 'rotator cuff lesion' OR 'rotator cuff lesions' OR 'rotator cuff pathology' OR 'rotator cuff tendinopathies' OR 'rotator cuff tendinopathy' OR 'shoulder rotator cuff degeneration' OR 'shoulder rotator cuff lesion' OR 'rotator cuff injury' OR 'quadrangular space syndrome' OR 'quadrilateral space syndrome' OR 'rotator cuff ruptures' OR 'rotator cuff tear' OR 'rotator cuff tears' OR 'rotatory cuff rupture' OR 'shoulder rotator cuff rupture' OR 'shoulder rotator cuff tear' OR 'rotator cuff rupture' OR 'apatite-associated destructive arthritis' OR 'cuff tear arthropathy' OR 'milwaukee shoulder' OR 'milwaukee shoulder syndrome' OR 'rotator cuff tear arthropathy' OR (('collateral ligament, ulnar' OR 'ligamentum collaterale ulnare' OR 'ulna collateral ligament' OR 'ulnar collateral ligament') AND ('ligament injuries' OR 'ligament trauma' OR 'ligament injury' OR 'ligament rupture')) OR 'fracture, meniscus' OR 'knee meniscal rupture' OR 'knee meniscus injury' OR 'knee meniscus lesion' OR 'knee meniscus trauma' OR 'lesion, meniscus' OR 'meniscal fracture' OR 'meniscal lesion' OR 'meniscal lesions' OR 'meniscal rupture, knee' OR 'meniscopathy' OR 'meniscus damage' OR 'meniscus fracture' OR 'meniscus injury' OR 'meniscus lesion' OR 'meniscus rupture, knee' OR 'meniscus tear' OR 'meniscus trauma' OR 'rupture, meniscus' OR 'tibial meniscus injuries' OR 'torn meniscus' OR 'trauma, meniscus' OR ('knee meniscus rupture' NOT (eye OR ocular OR ophtalm*)) OR 'ankle joint sprain' OR 'ankle ligament sprain' OR 'sprain, ankle' OR 'sprained ankle' OR 'ankle sprain' OR (('ankle ligament, lateral' OR 'lateral ligament, ankle' OR 'ligament, ankle, lateral' OR 'ankle lateral ligament') AND ('ligament injuries' OR 'ligament trauma' OR 'ligament injury' OR 'ligament rupture')) OR ('hip flexor*' AND (strain* OR trauma* OR injur*)) OR 'groin pull*' OR 'pulled groin*' OR 'groin injur*' OR 'groin strain*' OR 'hamstring pull*' OR 'pulled hamstring*' OR 'hamstring injur*' OR 'hamstring strain*' | 73216 |
| #5 | 'arthralgia'/exp OR 'myalgia'/de OR 'exercise induced muscle pain'/exp OR 'muscle soreness'/exp OR 'soreness'/de OR 'muscle injury'/exp OR 'joint injury'/exp OR 'foot injury'/exp OR 'medial tibial stress syndrome'/exp OR 'ligament and tendon injury'/exp OR 'muscle strain'/exp OR 'sprain'/exp OR 'exercise induced muscle damage'/de OR 'exertional pain'/de OR 'arthrodynia' OR 'joint ache' OR 'joint pain' OR 'arthralgia' OR myalgia OR 'muscle pain*' OR 'muscle soreness*' OR 'joint injur*' OR 'ligament injur*' OR 'tendon injur*' OR 'shin splint*' OR sprain* OR 'muscle strain*' OR 'muscle injur*' OR 'muscular pain*' OR 'muscular sore*' OR 'muscular injur*' OR 'strained muscle*' OR 'injured muscle*' OR 'sore muscle*' | 495698 |
| #6 | ((pain* NEAR/3 (relie* OR manag* OR control* OR mitigat* OR reduc*)):ti,ab,kw) OR analgesi*:ti,ab,kw OR 'analgesia'/de OR 'patient controlled analgesia'/de | 499402 |
| #7 | 'analgesic agent'/exp/dd_tp,dd_td | 14115 |
| #8 | 'lidocaine':ti,ab,kw,de OR '2 (diethylamino) n (2, 6 dimethylbenzene) acetamide':ti,ab,kw,de OR '2 (diethylamino) n (2, 6 dimethylphenyl) acetamide':ti,ab,kw,de OR '2 diethylamino 2`, 6` acetoxylidide':ti,ab,kw,de OR 'acoin':ti,ab,kw,de OR 'actilogic':ti,ab,kw,de OR 'aeroderm':ti,ab,kw,de OR 'after burn spray':ti,ab,kw,de OR 'akten':ti,ab,kw,de OR 'algrx 3268':ti,ab,kw,de OR 'algrx3268':ti,ab,kw,de OR 'alpha diethylamino 2, 6 dimethylacetanilide':ti,ab,kw,de OR 'alphacaine':ti,ab,kw,de OR 'anestacaine':ti,ab,kw,de OR 'anestacon':ti,ab,kw,de OR 'anestacone':ti,ab,kw,de OR 'aritmal':ti,ab,kw,de OR 'asensil':ti,ab,kw,de OR 'basicaina':ti,ab,kw,de OR 'betacaine':ti,ab,kw,de OR 'bobodent':ti,ab,kw,de OR 'bravera control':ti,ab,kw,de OR 'calmotop':ti,ab,kw,de OR 'cidancaina':ti,ab,kw,de OR 'col 1077':ti,ab,kw,de OR 'col1077':ti,ab,kw,de OR 'corus 1030':ti,ab,kw,de OR 'corus1030':ti,ab,kw,de OR 'cuivasil spray':ti,ab,kw,de OR 'dalcaine':ti,ab,kw,de OR 'dentinox':ti,ab,kw,de OR 'dentipatch':ti,ab,kw,de OR 'dequaspray':ti,ab,kw,de OR 'dermovagisil':ti,ab,kw,de OR 'diethylamino 2, 6 dimethylacetanilide hydrochloride':ti,ab,kw,de OR 'diethylaminodimethylacetanilid hydrochloride':ti,ab,kw,de OR 'diethylaminodimethylacetanilide hydrochloride':ti,ab,kw,de OR 'dolicaine':ti,ab,kw,de OR 'dolocopin':ti,ab,kw,de OR 'dube spray':ti,ab,kw,de OR 'duncaine':ti,ab,kw,de OR 'dynexan':ti,ab,kw,de OR 'dynexangival':ti,ab,kw,de OR 'ecocain':ti,ab,kw,de OR 'ela-max':ti,ab,kw,de OR 'esliva':ti,ab,kw,de OR 'esracain':ti,ab,kw,de OR 'esracaine':ti,ab,kw,de OR 'farmacaina':ti,ab,kw,de OR 'gesicain':ti,ab,kw,de OR 'glydo':ti,ab,kw,de OR 'gravocain':ti,ab,kw,de OR 'instillido':ti,ab,kw,de OR 'isicaine':ti,ab,kw,de OR 'jetocaine':ti,ab,kw,de OR 'jetokain':ti,ab,kw,de OR 'l-caine':ti,ab,kw,de OR 'lambdalina':ti,ab,kw,de OR 'lecasin':ti,ab,kw,de OR 'leostesin':ti,ab,kw,de OR 'licain':ti,ab,kw,de OR 'lida mantle':ti,ab,kw,de OR 'lidbree':ti,ab,kw,de OR 'lidiaq':ti,ab,kw,de OR 'lidiq':ti,ab,kw,de OR 'lido-tec':ti,ab,kw,de OR 'lidocain':ti,ab,kw,de OR 'lidocard':ti,ab,kw,de OR 'lidocaton':ti,ab,kw,de OR 'lidocor':ti,ab,kw,de OR 'lidocorit':ti,ab,kw,de OR 'lidoderm':ti,ab,kw,de OR 'lidofast':ti,ab,kw,de OR 'lidogalen':ti,ab,kw,de OR 'lidonest':ti,ab,kw,de OR 'lidonostrum':ti,ab,kw,de OR 'lidopain':ti,ab,kw,de OR 'lidopen':ti,ab,kw,de OR 'lidoposterin':ti,ab,kw,de OR 'lidorx':ti,ab,kw,de OR 'lidosen':ti,ab,kw,de OR 'lidotec':ti,ab,kw,de OR 'lidothesin':ti,ab,kw,de OR 'lignocaine':ti,ab,kw,de OR 'lignostab':ti,ab,kw,de OR 'lignox':ti,ab,kw,de OR 'lincaine':ti,ab,kw,de OR 'linisol':ti,ab,kw,de OR 'liquocaine':ti,ab,kw,de OR 'liris':ti,ab,kw,de OR 'll 30':ti,ab,kw,de OR 'll30':ti,ab,kw,de OR 'lmx 4':ti,ab,kw,de OR 'lmx 5':ti,ab,kw,de OR 'lmx4':ti,ab,kw,de OR 'lonioban':ti,ab,kw,de OR 'luan':ti,ab,kw,de OR 'maricaine':ti,ab,kw,de OR 'maxilene (lidocaine)':ti,ab,kw,de OR 'medocaine':ti,ab,kw,de OR 'n, n diethyl n` (2, 6 dimethylphenyl) glycinamide':ti,ab,kw,de OR 'nanolipo':ti,ab,kw,de OR 'neo lidocaton':ti,ab,kw,de OR 'neo novutox':ti,ab,kw,de OR 'neolidocaton':ti,ab,kw,de OR 'nydriar':ti,ab,kw,de OR 'omega diethylamino 2, 6 dimethylacetanilide':ti,ab,kw,de OR 'ophtesic':ti,ab,kw,de OR 'ortodermina':ti,ab,kw,de OR 'otipax':ti,ab,kw,de OR 'otocalmine':ti,ab,kw,de OR 'penles':ti,ab,kw,de OR 'posterisan akut':ti,ab,kw,de OR 'radiaguard':ti,ab,kw,de OR 'ralvo':ti,ab,kw,de OR 'remicaine gel':ti,ab,kw,de OR 'roxicaina':ti,ab,kw,de OR 'rucaina':ti,ab,kw,de OR 'ruciana':ti,ab,kw,de OR 'ryligency':ti,ab,kw,de OR 'solarcaine':ti,ab,kw,de OR 'solcaine':ti,ab,kw,de OR 'sp 103':ti,ab,kw,de OR 'sp103':ti,ab,kw,de OR 'trachilid':ti,ab,kw,de OR 'trachisan':ti,ab,kw,de OR 'truvinut':ti,ab,kw,de OR 'truxacaine':ti,ab,kw,de OR 'uad caine':ti,ab,kw,de OR 'vagisil':ti,ab,kw,de OR 'vasocaine':ti,ab,kw,de OR 'versatis':ti,ab,kw,de OR 'vessatis':ti,ab,kw,de OR 'vobolon':ti,ab,kw,de OR 'xidocaine':ti,ab,kw,de OR 'xilina':ti,ab,kw,de OR 'xiline':ti,ab,kw,de OR 'xilocaina viscosa':ti,ab,kw,de OR 'xilonest pomada':ti,ab,kw,de OR 'xilonibsa':ti,ab,kw,de OR 'xilotane gel':ti,ab,kw,de OR 'xilyne':ti,ab,kw,de OR 'xylanaest':ti,ab,kw,de OR 'xylcaine':ti,ab,kw,de OR 'xylestesin':ti,ab,kw,de OR 'xylocain':ti,ab,kw,de OR 'xylocaina':ti,ab,kw,de OR 'xylocaine':ti,ab,kw,de OR 'xylocard':ti,ab,kw,de OR 'xylocitin':ti,ab,kw,de OR 'xylocontact':ti,ab,kw,de OR 'xyloctin':ti,ab,kw,de OR 'xylojell':ti,ab,kw,de OR 'xyloneural':ti,ab,kw,de OR 'xylonor':ti,ab,kw,de OR 'xyloproct n':ti,ab,kw,de OR 'xyloton':ti,ab,kw,de OR 'xylotox':ti,ab,kw,de OR 'xylozan':ti,ab,kw,de OR 'xylyne':ti,ab,kw,de OR 'zingo':ti,ab,kw,de OR 'ztlido':ti,ab,kw,de OR 'salicylic acid methyl ester':ti,ab,kw,de OR '2 hydroxybenzoic acid methyl ester':ti,ab,kw,de OR 'betula oil':ti,ab,kw,de OR 'gaultheria oil':ti,ab,kw,de OR 'methyl 2 hydroxybenzoate':ti,ab,kw,de OR 'methyl salicylate':ti,ab,kw,de OR 'methylsalicylate':ti,ab,kw,de OR 'oil of wintergreen':ti,ab,kw,de OR 'sweet birch oil':ti,ab,kw,de OR 'wintergreen oil':ti,ab,kw,de OR 'hydroxyethyl-salicylate':ti,ab,kw,de OR '2 hydroxyethyl salicylate':ti,ab,kw,de OR 'ethyl glycol salicylate':ti,ab,kw,de OR 'ethylene glycol monosalicylate':ti,ab,kw,de OR 'ethylene glycol salicylate':ti,ab,kw,de OR 'ethylene glycol salicylic ester':ti,ab,kw,de OR 'ethyleneglycol monosalicylate':ti,ab,kw,de OR 'ethyleneglycol monosalicylic acid':ti,ab,kw,de OR 'gl 7':ti,ab,kw,de OR 'glycol monosalicylate':ti,ab,kw,de OR 'glycolsalicylate':ti,ab,kw,de OR 'glysal':ti,ab,kw,de OR 'monoglycol salicylate':ti,ab,kw,de OR 'phardol mono':ti,ab,kw,de OR 'rheumacyl':ti,ab,kw,de OR 'salicylic acid 2 hydroxyethyl ester':ti,ab,kw,de OR 'salicylic acid glycol ester':ti,ab,kw,de OR 'salicylic acid monoglycol ester':ti,ab,kw,de OR 'spirosal':ti,ab,kw,de OR 'traumasenex':ti,ab,kw,de OR 'glycol salicylate':ti,ab,kw,de OR 'ethyl salicylate':ti,ab,kw,de OR 'tetrahydrofurfuryl salicylate':ti,ab,kw,de OR 'diethylamine salicylate':ti,ab,kw,de OR 'acetylsalicylic acid':ti,ab,kw,de OR 'salicylic acid':ti,ab,kw,de OR 'methyl nicotinate':ti,ab,kw,de OR 'methylnicotinate':ti,ab,kw,de OR 'midalgan':ti,ab,kw,de OR 'nicotinic acid methyl ester':ti,ab,kw,de OR 'hexyl nicotinate':ti,ab,kw,de OR 'hexylnicotinate':ti,ab,kw,de OR 'nicotinic acid hexyl ester':ti,ab,kw,de OR 'benzylnicotinate':ti,ab,kw,de OR 'nicotinic acid benzyl ester':ti,ab,kw,de OR 'pycaril':ti,ab,kw,de OR 'revulsin':ti,ab,kw,de OR 'rubriment':ti,ab,kw,de OR 'benzyl nicotinate':ti,ab,kw,de OR 'hh 50':ti,ab,kw,de OR 'n (3 methoxy 4 hydroxybenzyl) nonanamide':ti,ab,kw,de OR 'n (4 hydroxy 3 methoxybenzyl) nonanamide':ti,ab,kw,de OR 'n nonanoylvanillylamine':ti,ab,kw,de OR 'n vanillylnonamide':ti,ab,kw,de OR 'n vanillylnonanamide':ti,ab,kw,de OR 'nonanoic acid vanillylamide':ti,ab,kw,de OR 'nonanoylvanillylamine':ti,ab,kw,de OR 'nonilylic acid vanillylamide':ti,ab,kw,de OR 'nonylic acid vanillylamide':ti,ab,kw,de OR 'nonylvanillamide':ti,ab,kw,de OR 'vanillyl n nonoylamide':ti,ab,kw,de OR 'vanillyl n nonylamide':ti,ab,kw,de OR 'vanillylnonamide':ti,ab,kw,de OR 'vanillylnonanamide':ti,ab,kw,de OR 'nonivamide':ti,ab,kw,de OR 'nonivamid':ti,ab,kw,de OR '2 butoxyethyl nicotinate':ti,ab,kw,de OR 'butoxyethyl nicotinate':ti,ab,kw,de OR 'n 51':ti,ab,kw,de OR 'nicotinic acid 2 butoxyethyl ester':ti,ab,kw,de OR 'nicoboxil':ti,ab,kw,de OR 'diclofenac':ti,ab,kw,de OR '2 (2, 6 dichloroanilino) phenylacetic acid':ti,ab,kw,de OR '2 (2, 6 dichlorophenylamino) phenylacetic acid':ti,ab,kw,de OR '2 [ (2, 6 dichlorophenyl) amino] benzeneacetic acid':ti,ab,kw,de OR '2 [2 [ (2, 6 dichlorophenyl) amino] phenyl] acetic acid':ti,ab,kw,de OR 'abdiflam':ti,ab,kw,de OR 'abitren':ti,ab,kw,de OR 'acuflam':ti,ab,kw,de OR 'akis':ti,ab,kw,de OR 'algipatch':ti,ab,kw,de OR 'algistick':ti,ab,kw,de OR 'algopain eze':ti,ab,kw,de OR 'algoplast':ti,ab,kw,de OR 'allvoran':ti,ab,kw,de OR 'almiral':ti,ab,kw,de OR 'almiral gel':ti,ab,kw,de OR 'almiral sr':ti,ab,kw,de OR 'alonpin':ti,ab,kw,de OR 'amz 001':ti,ab,kw,de OR 'amz001':ti,ab,kw,de OR 'antalcalm':ti,ab,kw,de OR 'apo-diclofenac ec':ti,ab,kw,de OR 'arcanafenac':ti,ab,kw,de OR 'arthrifen':ti,ab,kw,de OR 'artren':ti,ab,kw,de OR 'artrenac':ti,ab,kw,de OR 'artrites':ti,ab,kw,de OR 'artrites retard':ti,ab,kw,de OR 'assaren':ti,ab,kw,de OR 'athrofen':ti,ab,kw,de OR 'ba 47210':ti,ab,kw,de OR 'ba47210':ti,ab,kw,de OR 'batafil':ti,ab,kw,de OR 'berafen gel':ti,ab,kw,de OR 'berifen':ti,ab,kw,de OR 'berifen gel':ti,ab,kw,de OR 'betaren':ti,ab,kw,de OR 'bolabomin':ti,ab,kw,de OR 'calozan':ti,ab,kw,de OR 'catanac':ti,ab,kw,de OR 'catas':ti,ab,kw,de OR 'cencenag':ti,ab,kw,de OR 'clo-far':ti,ab,kw,de OR 'clofec':ti,ab,kw,de OR 'clofen':ti,ab,kw,de OR 'clonac':ti,ab,kw,de OR 'clonaren':ti,ab,kw,de OR 'clonodifen':ti,ab,kw,de OR 'cordralan':ti,ab,kw,de OR 'curinflam':ti,ab,kw,de OR 'ddl plaster':ti,ab,kw,de OR 'declophen':ti,ab,kw,de OR 'decrol':ti,ab,kw,de OR 'deflam-k':ti,ab,kw,de OR 'deflamat':ti,ab,kw,de OR 'delimon (diclofenac)':ti,ab,kw,de OR 'delphinac':ti,ab,kw,de OR 'denaclof':ti,ab,kw,de OR 'depain':ti,ab,kw,de OR 'depain plaster':ti,ab,kw,de OR 'dic 075v':ti,ab,kw,de OR 'dic075v':ti,ab,kw,de OR 'diceus':ti,ab,kw,de OR 'dicipan':ti,ab,kw,de OR 'diclac':ti,ab,kw,de OR 'diclac duo':ti,ab,kw,de OR 'diclax':ti,ab,kw,de OR 'diclax sr':ti,ab,kw,de OR 'diclo':ti,ab,kw,de OR 'diclo basan':ti,ab,kw,de OR 'diclo divido':ti,ab,kw,de OR 'diclo divido long':ti,ab,kw,de OR 'diclo puren':ti,ab,kw,de OR 'diclo recip':ti,ab,kw,de OR 'diclo-basan':ti,ab,kw,de OR 'diclobasan':ti,ab,kw,de OR 'diclobene':ti,ab,kw,de OR 'diclod':ti,ab,kw,de OR 'diclodent':ti,ab,kw,de OR 'diclodoc':ti,ab,kw,de OR 'diclodolor':ti,ab,kw,de OR 'diclofen':ti,ab,kw,de OR 'diclofen cremogel':ti,ab,kw,de OR 'diclofenac rekur':ti,ab,kw,de OR 'diclofenac resin':ti,ab,kw,de OR 'diclofenac resinate':ti,ab,kw,de OR 'diclofenac sodium':ti,ab,kw,de OR 'dicloflam':ti,ab,kw,de OR 'diclohexal':ti,ab,kw,de OR 'dicloin':ti,ab,kw,de OR 'diclomax':ti,ab,kw,de OR 'diclomol':ti,ab,kw,de OR 'diclon':ti,ab,kw,de OR 'diclopax':ti,ab,kw,de OR 'diclophenac sodium':ti,ab,kw,de OR 'diclopuren':ti,ab,kw,de OR 'dicloral':ti,ab,kw,de OR 'dicloran gel':ti,ab,kw,de OR 'diclorecip':ti,ab,kw,de OR 'dicloren':ti,ab,kw,de OR 'dicloreum':ti,ab,kw,de OR 'diclosan sr':ti,ab,kw,de OR 'diclosian':ti,ab,kw,de OR 'diclotec':ti,ab,kw,de OR 'diclowal':ti,ab,kw,de OR 'dicsnal':ti,ab,kw,de OR 'difen':ti,ab,kw,de OR 'difena':ti,ab,kw,de OR 'difenac':ti,ab,kw,de OR 'difenol gel':ti,ab,kw,de OR 'difnal k':ti,ab,kw,de OR 'dioxaflex':ti,ab,kw,de OR 'dioxaflex retard':ti,ab,kw,de OR 'divoltar':ti,ab,kw,de OR 'dixol':ti,ab,kw,de OR 'dixol forte':ti,ab,kw,de OR 'doflastad':ti,ab,kw,de OR 'doflex':ti,ab,kw,de OR 'dolaren':ti,ab,kw,de OR 'dolaut gel':ti,ab,kw,de OR 'dolflam-retard':ti,ab,kw,de OR 'dolo voltaren':ti,ab,kw,de OR 'doloflam':ti,ab,kw,de OR 'dolotren':ti,ab,kw,de OR 'dolotren gel':ti,ab,kw,de OR 'dolotren retard':ti,ab,kw,de OR 'doragon':ti,ab,kw,de OR 'dosanac':ti,ab,kw,de OR 'duravolten':ti,ab,kw,de OR 'dycon sr':ti,ab,kw,de OR 'dyloject':ti,ab,kw,de OR 'ecofenac':ti,ab,kw,de OR 'econac':ti,ab,kw,de OR 'effekton':ti,ab,kw,de OR 'effekton retard':ti,ab,kw,de OR 'eflagen':ti,ab,kw,de OR 'epifenac':ti,ab,kw,de OR 'eslofen':ti,ab,kw,de OR 'evadol':ti,ab,kw,de OR 'evinopon':ti,ab,kw,de OR 'feloran':ti,ab,kw,de OR 'fenac':ti,ab,kw,de OR 'fenadium':ti,ab,kw,de OR 'fenaspec':ti,ab,kw,de OR 'flameril':ti,ab,kw,de OR 'flexagen':ti,ab,kw,de OR 'flogofenac':ti,ab,kw,de OR 'flogosin d':ti,ab,kw,de OR 'flogozan':ti,ab,kw,de OR 'fortfen sr':ti,ab,kw,de OR 'freejex':ti,ab,kw,de OR 'gavin (diclofenac)':ti,ab,kw,de OR 'glimbax (diclofenac)':ti,ab,kw,de OR 'gp 45840':ti,ab,kw,de OR 'grofenac':ti,ab,kw,de OR 'hizemin':ti,ab,kw,de OR 'imflac':ti,ab,kw,de OR 'inac gel':ti,ab,kw,de OR 'indicam':ti,ab,kw,de OR 'inflamac':ti,ab,kw,de OR 'inflanac':ti,ab,kw,de OR 'inforce (drug)':ti,ab,kw,de OR 'isv 205':ti,ab,kw,de OR 'isv205':ti,ab,kw,de OR 'jonac gel':ti,ab,kw,de OR 'kadiflam':ti,ab,kw,de OR 'kinespir':ti,ab,kw,de OR 'klofen l':ti,ab,kw,de OR 'klotaren':ti,ab,kw,de OR 'kriplex':ti,ab,kw,de OR 'lesflam':ti,ab,kw,de OR 'leviogel':ti,ab,kw,de OR 'lifenac':ti,ab,kw,de OR 'lofenac':ti,ab,kw,de OR 'lotirac':ti,ab,kw,de OR 'lynxorb':ti,ab,kw,de OR 'magluphen':ti,ab,kw,de OR 'merflam':ti,ab,kw,de OR 'modifenac':ti,ab,kw,de OR 'monoflam':ti,ab,kw,de OR 'motifene':ti,ab,kw,de OR 'naboal':ti,ab,kw,de OR 'nac gel':ti,ab,kw,de OR 'naclof':ti,ab,kw,de OR 'nacoflar':ti,ab,kw,de OR 'nadifen':ti,ab,kw,de OR 'novapirina':ti,ab,kw,de OR 'novo-difenac':ti,ab,kw,de OR 'novolten':ti,ab,kw,de OR 'ofenac':ti,ab,kw,de OR 'olfen':ti,ab,kw,de OR 'olfen gel':ti,ab,kw,de OR 'olfen roll-on':ti,ab,kw,de OR 'olfen-75 sr':ti,ab,kw,de OR 'optanac':ti,ab,kw,de OR 'orthophen':ti,ab,kw,de OR 'ortofen forte':ti,ab,kw,de OR 'osteoflam':ti,ab,kw,de OR 'painstop':ti,ab,kw,de OR 'panamor':ti,ab,kw,de OR 'pennsaid':ti,ab,kw,de OR 'profenac':ti,ab,kw,de OR 'prosorb-d':ti,ab,kw,de OR 'relaxyl gel':ti,ab,kw,de OR 'remethan':ti,ab,kw,de OR 'remethan gel':ti,ab,kw,de OR 'renvol emulgel':ti,ab,kw,de OR 'rewodina':ti,ab,kw,de OR 'rheufenac':ti,ab,kw,de OR 'rheumafen':ti,ab,kw,de OR 'rheumatac':ti,ab,kw,de OR 'rheumatac retard':ti,ab,kw,de OR 'rhewlin':ti,ab,kw,de OR 'rhewlin forte':ti,ab,kw,de OR 'rhewlin sr':ti,ab,kw,de OR 'rhumalgan':ti,ab,kw,de OR 'rolactin':ti,ab,kw,de OR 'sailib':ti,ab,kw,de OR 'savismin':ti,ab,kw,de OR 'sefnac':ti,ab,kw,de OR 'slofenac':ti,ab,kw,de OR 'sodium diclofenac':ti,ab,kw,de OR 'solaraze':ti,ab,kw,de OR 'sophenoderm':ti,ab,kw,de OR 'soproxen':ti,ab,kw,de OR 'spraymik':ti,ab,kw,de OR 'sr 318t':ti,ab,kw,de OR 'staren':ti,ab,kw,de OR 'sting gel':ti,ab,kw,de OR 'tabiflex':ti,ab,kw,de OR 'tds 943':ti,ab,kw,de OR 'tds943':ti,ab,kw,de OR 'tigen plaster':ti,ab,kw,de OR 'toraren':ti,ab,kw,de OR 'traulen':ti,ab,kw,de OR 'tsudohmin':ti,ab,kw,de OR 'uniclonax':ti,ab,kw,de OR 'uniren':ti,ab,kw,de OR 'urigon (diclofenac)':ti,ab,kw,de OR 'valentac':ti,ab,kw,de OR 'vartelon':ti,ab,kw,de OR 'vartelon gel':ti,ab,kw,de OR 'veral':ti,ab,kw,de OR 'voldal':ti,ab,kw,de OR 'voldic':ti,ab,kw,de OR 'voldic emulgel':ti,ab,kw,de OR 'volero':ti,ab,kw,de OR 'volfenac':ti,ab,kw,de OR 'volna-k':ti,ab,kw,de OR 'volsaid':ti,ab,kw,de OR 'volsaid retard':ti,ab,kw,de OR 'volta':ti,ab,kw,de OR 'voltadex emulgel':ti,ab,kw,de OR 'voltadvance':ti,ab,kw,de OR 'voltalen':ti,ab,kw,de OR 'voltalen emulgel':ti,ab,kw,de OR 'voltalgan':ti,ab,kw,de OR 'voltaren':ti,ab,kw,de OR 'voltaren acti-go':ti,ab,kw,de OR 'voltaren arthritis pain':ti,ab,kw,de OR 'voltaren colirio':ti,ab,kw,de OR 'voltaren forte':ti,ab,kw,de OR 'voltaren gel':ti,ab,kw,de OR 'voltaren k migraine':ti,ab,kw,de OR 'voltaren ofta':ti,ab,kw,de OR 'voltaren oftalmico':ti,ab,kw,de OR 'voltaren ophta':ti,ab,kw,de OR 'voltaren ophtha':ti,ab,kw,de OR 'voltaren ophthalmic':ti,ab,kw,de OR 'voltaren retard':ti,ab,kw,de OR 'voltaren spe':ti,ab,kw,de OR 'voltaren sr':ti,ab,kw,de OR 'voltaren xr':ti,ab,kw,de OR 'voltaren-xr':ti,ab,kw,de OR 'voltarene':ti,ab,kw,de OR 'voltarenspe':ti,ab,kw,de OR 'voltarol':ti,ab,kw,de OR 'voltarol 50':ti,ab,kw,de OR 'voltarol one a day muscle pain relief':ti,ab,kw,de OR 'voltarol rapid':ti,ab,kw,de OR 'voltarol retard':ti,ab,kw,de OR 'voltine':ti,ab,kw,de OR 'voltral':ti,ab,kw,de OR 'voltrix':ti,ab,kw,de OR 'voren emulgel':ti,ab,kw,de OR 'votalen':ti,ab,kw,de OR 'votalen sr':ti,ab,kw,de OR 'voveran':ti,ab,kw,de OR 'voveran emulgel':ti,ab,kw,de OR 'vurdon':ti,ab,kw,de OR 'wergyl':ti,ab,kw,de OR 'xenid':ti,ab,kw,de OR 'yuren':ti,ab,kw,de OR 'zolterol':ti,ab,kw,de OR 'zolterol sr':ti,ab,kw,de OR 'zorvolex':ti,ab,kw,de OR 'dolostrip':ti,ab,kw,de OR 'voltarenplast':ti,ab,kw,de OR nsaids:ti,ab,kw,de OR paracetamol:ti,ab,kw,de OR ibuprofen:ti,ab,kw,de OR naproxen:ti,ab,kw,de OR 'algesalona (etofenamate)':ti,ab,kw,de OR 'algesalone':ti,ab,kw,de OR 'bay d 1107':ti,ab,kw,de OR 'bay d1107':ti,ab,kw,de OR 'bayro gel':ti,ab,kw,de OR 'bayrogel':ti,ab,kw,de OR 'bph 485':ti,ab,kw,de OR 'bph485':ti,ab,kw,de OR 'etophenamate':ti,ab,kw,de OR 'flufenamic acid 2 (2 hydroxyethyl) ethyl ester':ti,ab,kw,de OR 'reumagel':ti,ab,kw,de OR 'rheumon':ti,ab,kw,de OR 'rheumon gel':ti,ab,kw,de OR 'traumon':ti,ab,kw,de OR 'ty 485':ti,ab,kw,de OR 'ty485':ti,ab,kw,de OR 'etofenamate':ti,ab,kw,de OR '2 [2 (6, 6 dimethylbicyclo (3.1.1) hept 2 en 2 yl) ethoxy] triethylamine':ti,ab,kw,de OR 'algesal suractive':ti,ab,kw,de OR 'homomyrtenyloxytriethylamine':ti,ab,kw,de OR 'nopoxamine':ti,ab,kw,de OR 'myrtecaine':ti,ab,kw,de OR '1, 2 diphenyl 3, 5 dioxo 4 n butylpyrazolidine':ti,ab,kw,de OR '1, 2 diphenyl 3, 5 dioxo 4 normal butylpyrazolidine':ti,ab,kw,de OR '3, 5 dioxo 1, 2 diphenyl 4 n butylpyrazolidine':ti,ab,kw,de OR '4 butyl 1, 2 diphenyl 3, 5 dioxopyrazolidine':ti,ab,kw,de OR '4 butyl 1, 2 diphenyl 3, 5 pyrazolidinedione':ti,ab,kw,de OR 'alindor':ti,ab,kw,de OR 'alka butazolidin':ti,ab,kw,de OR 'alpha phenylbutazone':ti,ab,kw,de OR 'antadol':ti,ab,kw,de OR 'anuspiramin':ti,ab,kw,de OR 'anuspiranim':ti,ab,kw,de OR 'apo-phenylbutazone':ti,ab,kw,de OR 'artgrizone':ti,ab,kw,de OR 'arthril':ti,ab,kw,de OR 'arthrizin':ti,ab,kw,de OR 'arthrizon':ti,ab,kw,de OR 'arthryl (phenylbutazone)':ti,ab,kw,de OR 'artrizin':ti,ab,kw,de OR 'autadol':ti,ab,kw,de OR 'azolid':ti,ab,kw,de OR 'baliomel':ti,ab,kw,de OR 'basireuma':ti,ab,kw,de OR 'bumzolidina':ti,ab,kw,de OR 'busone':ti,ab,kw,de OR 'butacote':ti,ab,kw,de OR 'butadion':ti,ab,kw,de OR 'butadiona':ti,ab,kw,de OR 'butadione':ti,ab,kw,de OR 'butalidon':ti,ab,kw,de OR 'butapirazol':ti,ab,kw,de OR 'butapyrasol':ti,ab,kw,de OR 'butartril':ti,ab,kw,de OR 'butasan':ti,ab,kw,de OR 'butatab':ti,ab,kw,de OR 'butazina':ti,ab,kw,de OR 'butazine':ti,ab,kw,de OR 'butazolidin':ti,ab,kw,de OR 'butazolidin ointment':ti,ab,kw,de OR 'butazolidina':ti,ab,kw,de OR 'butazolidine':ti,ab,kw,de OR 'butazone-100':ti,ab,kw,de OR 'butidiona':ti,ab,kw,de OR 'butidione':ti,ab,kw,de OR 'butrex':ti,ab,kw,de OR 'butylpyridin':ti,ab,kw,de OR 'buzon':ti,ab,kw,de OR 'delta butazolidin':ti,ab,kw,de OR 'delta elmedal':ti,ab,kw,de OR 'diossidone':ti,ab,kw,de OR 'diozal':ti,ab,kw,de OR 'diphebuzol':ti,ab,kw,de OR | 1,327,903 |
|  | 'diphenbutazone':ti,ab,kw,de OR 'diphenylbutazone':ti,ab,kw,de OR 'ecobutazon':ti,ab,kw,de OR 'ecobutazone':ti,ab,kw,de OR 'elmedal':ti,ab,kw,de OR 'equipalazone':ti,ab,kw,de OR 'exrheudon n':ti,ab,kw,de OR 'febuzine':ti,ab,kw,de OR 'fenbutazona':ti,ab,kw,de OR 'fenibutazona':ti,ab,kw,de OR 'fenibutazone':ti,ab,kw,de OR 'fenibutol':ti,ab,kw,de OR 'fenilbutazona':ti,ab,kw,de OR 'fenilbutazona gen-far':ti,ab,kw,de OR 'fenilbutazona mckesson':ti,ab,kw,de OR 'fenilbutina':ti,ab,kw,de OR 'fenotone':ti,ab,kw,de OR 'fenyl butazon':ti,ab,kw,de OR 'fenylbutazone':ti,ab,kw,de OR 'g 13871':ti,ab,kw,de OR 'g13871':ti,ab,kw,de OR 'ia but':ti,ab,kw,de OR 'intrabutazone':ti,ab,kw,de OR 'intrazone':ti,ab,kw,de OR 'ircodin':ti,ab,kw,de OR 'irgapan':ti,ab,kw,de OR 'mcn r 495':ti,ab,kw,de OR 'nadozone':ti,ab,kw,de OR 'neuro elmedal':ti,ab,kw,de OR 'phebuzine':ti,ab,kw,de OR 'phenbutazole':ti,ab,kw,de OR 'phenbutazone':ti,ab,kw,de OR 'phenobutazone':ti,ab,kw,de OR 'phenyl butazone':ti,ab,kw,de OR 'phenylarthrite':ti,ab,kw,de OR 'phenylbutasone':ti,ab,kw,de OR 'phenylbutazon':ti,ab,kw,de OR 'phenylbutazone ointment':ti,ab,kw,de OR 'phenylbutazone sodium':ti,ab,kw,de OR 'phenylmobuzon':ti,ab,kw,de OR 'pirarreumol b':ti,ab,kw,de OR 'pirrareumol b':ti,ab,kw,de OR 'pyrabutol':ti,ab,kw,de OR 'pyrazolidin':ti,ab,kw,de OR 'pyrazone':ti,ab,kw,de OR 'r 3 zon':ti,ab,kw,de OR 'reudo':ti,ab,kw,de OR 'reudox':ti,ab,kw,de OR 'reumuzol':ti,ab,kw,de OR 'servizolidin':ti,ab,kw,de OR 'shigrodin':ti,ab,kw,de OR 'sodium phenylbutazone':ti,ab,kw,de OR 'spondyril':ti,ab,kw,de OR 'ticinil':ti,ab,kw,de OR 'zolandin':ti,ab,kw,de OR 'zolaphen':ti,ab,kw,de OR 'phenylbutazone':ti,ab,kw,de OR '12, 19 difluoro 11 hydroxy 8 (2 hydroxyacetyl) 6, 6, 9, 13 tetramethyl 5, 7 dioxapentacyclo [10.8.0.0 (2, 9) .0 (4, 8) .0 (13, 18)] icosa 14, 17 dien 16 one':ti,ab,kw,de OR '4b, 12 difluoro 5 hydroxy 6b (hydroxyacetyl) 4a, 6a, 8, 8 tetramethyl 4a, 4b, 5, 6, 6a, 6b, 9a, 10, 10a, 10b, 11, 12 dodecahydro 2h naphtho [2`, 1`:4, 5] indeno [1, 2 d] [1, 3] dioxol 2 one':ti,ab,kw,de OR '4b, 12 difluoro 6b glycoloyl 5 hydroxy 4a, 6a, 8, 8 tetramethyl 4a, 4b, 5, 6, 6a, 6b, 9a, 10, 10a, 10b, 11, 12 dodecahydro 2h naphtho [2`, 1`:4, 5] indeno [1, 2 d] [1, 3] dioxol 2 one':ti,ab,kw,de OR '6, 9 difluoro 11, 21 dihydroxy 16, 17 [ (1 methylethylidene) bis (oxy)] pregna 1, 4 diene 3, 20 dione':ti,ab,kw,de OR '6alpha, 9alpha difluoro 11beta, 16alpha, 17alpha, 21 tetrahydroxy 1, 4 pregnadiene 3, 20 dione 16, 17 acetonide':ti,ab,kw,de OR '6alpha, 9alpha difluoro 16alpha hydroxyprednisolone 16, 17 acetonide':ti,ab,kw,de OR 'adermina':ti,ab,kw,de OR 'alfabios':ti,ab,kw,de OR 'alvadermo fuerte':ti,ab,kw,de OR 'aplosyn':ti,ab,kw,de OR 'capex':ti,ab,kw,de OR 'cervicum':ti,ab,kw,de OR 'cinolon':ti,ab,kw,de OR 'clofeet':ti,ab,kw,de OR 'cortilona':ti,ab,kw,de OR 'cremisona':ti,ab,kw,de OR 'cynozet':ti,ab,kw,de OR 'derma-smooth/fs':ti,ab,kw,de OR 'derma-smoothe/fs':ti,ab,kw,de OR 'dermalar':ti,ab,kw,de OR 'dermoflam':ti,ab,kw,de OR 'dermoran':ti,ab,kw,de OR 'dermotic':ti,ab,kw,de OR 'df 277':ti,ab,kw,de OR 'df277':ti,ab,kw,de OR 'ekzem (drug)':ti,ab,kw,de OR 'esacinone':ti,ab,kw,de OR 'flac (fluocinolone acetonide)':ti,ab,kw,de OR 'flozet':ti,ab,kw,de OR 'fluciderm':ti,ab,kw,de OR 'flucinar':ti,ab,kw,de OR 'flucinolone acetonide':ti,ab,kw,de OR 'flulone':ti,ab,kw,de OR 'flunolone-v':ti,ab,kw,de OR 'fluocet':ti,ab,kw,de OR 'fluocid':ti,ab,kw,de OR 'fluocid forte':ti,ab,kw,de OR 'fluocinolon acetonid':ti,ab,kw,de OR 'fluocinolone 16, 17 acetonide':ti,ab,kw,de OR 'fluocinonide acetonide':ti,ab,kw,de OR 'fluoderm':ti,ab,kw,de OR 'fluolar':ti,ab,kw,de OR 'fluonid':ti,ab,kw,de OR 'fluonide':ti,ab,kw,de OR 'fluotrex':ti,ab,kw,de OR 'fluovitef':ti,ab,kw,de OR 'fluquinol':ti,ab,kw,de OR 'flurosyn':ti,ab,kw,de OR 'flusonlen':ti,ab,kw,de OR 'fluzon':ti,ab,kw,de OR 'fs shampoo':ti,ab,kw,de OR 'fusalar':ti,ab,kw,de OR 'gelargin':ti,ab,kw,de OR 'gelidina':ti,ab,kw,de OR 'iluvien':ti,ab,kw,de OR 'inoderm':ti,ab,kw,de OR 'jellin':ti,ab,kw,de OR 'lluvien':ti,ab,kw,de OR 'localyn':ti,ab,kw,de OR 'localyn glicole':ti,ab,kw,de OR 'luci':ti,ab,kw,de OR 'medidur':ti,ab,kw,de OR 'nasal fluocinolone acetonide':ti,ab,kw,de OR 'nasal synalar':ti,ab,kw,de OR 'neosynalar':ti,ab,kw,de OR 'nsc 92339':ti,ab,kw,de OR 'nsc92339':ti,ab,kw,de OR 'ot 401':ti,ab,kw,de OR 'ot401':ti,ab,kw,de OR 'otazem':ti,ab,kw,de OR 'otoflamm':ti,ab,kw,de OR 'otoken':ti,ab,kw,de OR 'psoranide':ti,ab,kw,de OR 'radiocin':ti,ab,kw,de OR 'retisert':ti,ab,kw,de OR 'rs 1401 at':ti,ab,kw,de OR 'rs 1401at':ti,ab,kw,de OR 'rs1401 at':ti,ab,kw,de OR 'rs1401at':ti,ab,kw,de OR 'sinaflana':ti,ab,kw,de OR 'supralan':ti,ab,kw,de OR 'synalar':ti,ab,kw,de OR 'synalar 25':ti,ab,kw,de OR 'synalar forte':ti,ab,kw,de OR 'synalar gamma':ti,ab,kw,de OR 'synalar hp':ti,ab,kw,de OR 'synalar nasal':ti,ab,kw,de OR 'synalar simple':ti,ab,kw,de OR 'synalar-hp':ti,ab,kw,de OR 'synandone':ti,ab,kw,de OR 'synemol':ti,ab,kw,de OR 'synotic':ti,ab,kw,de OR 'syntopic':ti,ab,kw,de OR 'trisyn':ti,ab,kw,de OR 'yutiq':ti,ab,kw,de OR 'fluocinolone acetonide':ti,ab,kw,de OR piroxicam:ti,ab,kw,de OR '1 (3 dimethylaminopropylidene) 2, 3:6, 7 dibenzo 4 suberene':ti,ab,kw,de OR '3 (5h dibenzo [a, d] cyclohepten 5 ylidene) n, n dimethylpropylamine':ti,ab,kw,de OR '5 (3 dimethylaminopropylidene) (5h) dibenzo (a, d) cycloheptene':ti,ab,kw,de OR '5 (3 dimethylaminopropylidene) dibenzo (a, e) cycloheptatriene':ti,ab,kw,de OR '5 (3 dimethylaminopropylidene) dibenzo [a, e] cycloheptatriene':ti,ab,kw,de OR 'amrix':ti,ab,kw,de OR 'cyben':ti,ab,kw,de OR 'cyclobenzaprine hydrochloride':ti,ab,kw,de OR 'flexeril':ti,ab,kw,de OR 'flexiban':ti,ab,kw,de OR 'mk 130':ti,ab,kw,de OR 'mk130':ti,ab,kw,de OR 'n, n dimethyl 5h debenzo [a, d] cycloheptene delta5, alpha propylamine':ti,ab,kw,de OR 'proheptatriene':ti,ab,kw,de OR 'rp 9715':ti,ab,kw,de OR 'rp9715':ti,ab,kw,de OR 'tensodox':ti,ab,kw,de OR 'yurelax':ti,ab,kw,de OR 'cyclobenzaprine':ti,ab,kw,de OR '10, 11 dihydro 5 (gamma dimethylaminopropylidene) 5h dibenzo [a, d] 1, 4 cycloheptene':ti,ab,kw,de OR '10, 11 dihydro n, n dimethyl 5h dibenzo [a, d] cycloheptene delta5, gamma propylamine':ti,ab,kw,de OR '3 (10, 11 dihydro 5h dibenzo [a, d] cycloheptene 5 ylidene) n, n dimethylpropylamine':ti,ab,kw,de OR '5 (3 dimethylaminopropylidene) 10, 11 dihydro 5h dibenzo [a, d] cycloheptene':ti,ab,kw,de OR '5 (3 dimethylaminopropylidene) dibenzo [a, d] [1, 4] cycloheptadiene':ti,ab,kw,de OR '5 (gamma dimethylaminopropylidene) 5h dibenzo [a, d] 10, 11 dihydrocycloheptene':ti,ab,kw,de OR 'adepress':ti,ab,kw,de OR 'adepril':ti,ab,kw,de OR 'ambivalon':ti,ab,kw,de OR 'amilit':ti,ab,kw,de OR 'amineurin':ti,ab,kw,de OR 'amineurin retard':ti,ab,kw,de OR 'amiplin':ti,ab,kw,de OR 'amiprin':ti,ab,kw,de OR 'amirol':ti,ab,kw,de OR 'amirol 10 fc':ti,ab,kw,de OR 'amirol 25 fc':ti,ab,kw,de OR 'amitid':ti,ab,kw,de OR 'amitril':ti,ab,kw,de OR 'amitrip':ti,ab,kw,de OR 'amitriptylene':ti,ab,kw,de OR 'amitriptylene hydrochloride':ti,ab,kw,de OR 'amitriptylin':ti,ab,kw,de OR 'amitriptylin-ct':ti,ab,kw,de OR 'amitriptyline hydrochloride':ti,ab,kw,de OR 'amitriptylinumhydrochloride':ti,ab,kw,de OR 'amitryptiline':ti,ab,kw,de OR 'amitryptiline hydrochloride':ti,ab,kw,de OR 'amitryptilline':ti,ab,kw,de OR 'amitryptine':ti,ab,kw,de OR 'amitryptyline':ti,ab,kw,de OR 'amyline':ti,ab,kw,de OR 'amytril':ti,ab,kw,de OR 'amytriptiline':ti,ab,kw,de OR 'amytriptyline':ti,ab,kw,de OR 'amytryptiline':ti,ab,kw,de OR 'amyzol':ti,ab,kw,de OR 'anapsique':ti,ab,kw,de OR 'anp 3548':ti,ab,kw,de OR 'anp3548':ti,ab,kw,de OR 'antalin':ti,ab,kw,de OR 'antitriptyline':ti,ab,kw,de OR 'apo-amitriptyline':ti,ab,kw,de OR 'astilin':ti,ab,kw,de OR 'atx 01 (aliphatic amine)':ti,ab,kw,de OR 'atx01 (aliphatic amine)':ti,ab,kw,de OR 'damilen':ti,ab,kw,de OR 'damilene':ti,ab,kw,de OR 'damitriptyline':ti,ab,kw,de OR 'damylene':ti,ab,kw,de OR 'deprelio':ti,ab,kw,de OR 'domical':ti,ab,kw,de OR 'elatrol':ti,ab,kw,de OR 'elatrolet':ti,ab,kw,de OR 'elavil':ti,ab,kw,de OR 'enafon':ti,ab,kw,de OR 'endep':ti,ab,kw,de OR 'enovil':ti,ab,kw,de OR 'etafon':ti,ab,kw,de OR 'etafron':ti,ab,kw,de OR 'euplit':ti,ab,kw,de OR 'lantron':ti,ab,kw,de OR 'laroxal':ti,ab,kw,de OR 'laroxyl':ti,ab,kw,de OR 'lentizol':ti,ab,kw,de OR 'miketorin':ti,ab,kw,de OR 'mk 230':ti,ab,kw,de OR 'mk230':ti,ab,kw,de OR 'n 750':ti,ab,kw,de OR 'n, n dimethyl 3 (2 tricyclo [9.4.0.0 (3, 8)] pentadeca 1 (15), 3, 5, 7, 11, 13 hexaenylidene) propan 1 amine':ti,ab,kw,de OR 'n, n dimethyl 3 (dibenzo [a.d] 1, 4 cycloheptadien 5 yliden) propylamine hydrochloride':ti,ab,kw,de OR 'n750':ti,ab,kw,de OR 'novoprotect':ti,ab,kw,de OR 'ormal':ti,ab,kw,de OR 'pinsaun':ti,ab,kw,de OR 'proheptadien':ti,ab,kw,de OR 'qualitriptine':ti,ab,kw,de OR 'redomex':ti,ab,kw,de OR 'redomex diffucaps':ti,ab,kw,de OR 'ro 4 1575':ti,ab,kw,de OR 'ro4 1575':ti,ab,kw,de OR 'ro41575':ti,ab,kw,de OR 'sarboten retard 75':ti,ab,kw,de OR 'sarotard':ti,ab,kw,de OR 'saroten':ti,ab,kw,de OR 'saroten retard':ti,ab,kw,de OR 'sarotena':ti,ab,kw,de OR 'sarotex':ti,ab,kw,de OR 'sarotex retard':ti,ab,kw,de OR 'stelminal':ti,ab,kw,de OR 'sylvemid':ti,ab,kw,de OR 'syneudon':ti,ab,kw,de OR 'syneydon':ti,ab,kw,de OR 'teperin':ti,ab,kw,de OR 'terepin':ti,ab,kw,de OR 'trepiline':ti,ab,kw,de OR 'tridep':ti,ab,kw,de OR 'tripta':ti,ab,kw,de OR 'triptanol':ti,ab,kw,de OR 'triptizol':ti,ab,kw,de OR 'triptyl':ti,ab,kw,de OR 'triptyline':ti,ab,kw,de OR 'trynol':ti,ab,kw,de OR 'tryptanol':ti,ab,kw,de OR 'tryptizol':ti,ab,kw,de OR 'trytomer':ti,ab,kw,de OR 'uxen':ti,ab,kw,de OR 'vanatrip':ti,ab,kw,de OR 'amitriptyline':ti,ab,kw,de OR '2 (2 chlorophenyl) 2 (methylamino) cyclohexanone':ti,ab,kw,de OR '2 (2 chlorophenyl) 2 (methylamino) cyclohexanone hydrochloride':ti,ab,kw,de OR '2 (2 chlorophenyl) 2 methylaminocyclohexanone':ti,ab,kw,de OR '2 (methylamino) 2 (2 chlorophenyl) cyclohexanone':ti,ab,kw,de OR '2 (ortho chlorophenyl) 2 (methylamino) cyclohexanone':ti,ab,kw,de OR '2 (ortho chlorophenyl) 2 methylaminocyclohexanone':ti,ab,kw,de OR '2 (ortho chlorophenyl) 2 methylaminocyclohexanone hydrochloride':ti,ab,kw,de OR '2 methylamino 2 (2 chlorophenyl) cyclohexanone':ti,ab,kw,de OR '2 ortho chlorophenyl 2 methylaminocyclohexanone':ti,ab,kw,de OR 'anesject':ti,ab,kw,de OR 'calipsol':ti,ab,kw,de OR 'calypsol':ti,ab,kw,de OR 'ci 581':ti,ab,kw,de OR 'ci581':ti,ab,kw,de OR 'cl 369':ti,ab,kw,de OR 'cl369':ti,ab,kw,de OR 'cn 52, 372 2':ti,ab,kw,de OR 'cn 52372 2':ti,ab,kw,de OR 'cn 523722':ti,ab,kw,de OR 'cn52, 372 2':ti,ab,kw,de OR 'cn52372 2':ti,ab,kw,de OR 'cn523722':ti,ab,kw,de OR 'ereska':ti,ab,kw,de OR 'imalgene':ti,ab,kw,de OR 'kalipsol':ti,ab,kw,de OR 'katamine':ti,ab,kw,de OR 'keta-hameln':ti,ab,kw,de OR 'ketaject':ti,ab,kw,de OR 'ketalar':ti,ab,kw,de OR 'ketalin':ti,ab,kw,de OR 'ketamax':ti,ab,kw,de OR 'ketamine hcl':ti,ab,kw,de OR 'ketamine hydrochloride':ti,ab,kw,de OR 'ketaminol vet':ti,ab,kw,de OR 'ketanest':ti,ab,kw,de OR 'ketased':ti,ab,kw,de OR 'ketaset':ti,ab,kw,de OR 'ketaved':ti,ab,kw,de OR 'ketavet':ti,ab,kw,de OR 'ketmin':ti,ab,kw,de OR 'ketoject':ti,ab,kw,de OR 'ketolar':ti,ab,kw,de OR 'narkamon':ti,ab,kw,de OR 'narketan':ti,ab,kw,de OR 'pmi 100':ti,ab,kw,de OR 'pmi 150':ti,ab,kw,de OR 'pmi100':ti,ab,kw,de OR 'pmi150':ti,ab,kw,de OR 'sls 002':ti,ab,kw,de OR 'sls002':ti,ab,kw,de OR 'soon-soon':ti,ab,kw,de OR 'tekam':ti,ab,kw,de OR 'tur 002':ti,ab,kw,de OR 'tur002':ti,ab,kw,de OR 'velonarcon':ti,ab,kw,de OR 'vetalar':ti,ab,kw,de OR 'ketamine':ti,ab,kw,de OR '3 (4 chlorophenyl) 4 aminobutanoic acid':ti,ab,kw,de OR '3 (4 chlorophenyl) 4 aminobutyric acid':ti,ab,kw,de OR '3 (para chlorophenyl) 4 aminobutanoic acid':ti,ab,kw,de OR '3 (para chlorophenyl) 4 aminobutyric acid':ti,ab,kw,de OR '4 amino 3 (4 chlorophenyl) butanoic acid':ti,ab,kw,de OR '4 amino 3 (4 chlorophenyl) butyric acid':ti,ab,kw,de OR 'all 1401':ti,ab,kw,de OR 'all1401':ti,ab,kw,de OR 'alpha-baclofen':ti,ab,kw,de OR 'ba 34 647':ti,ab,kw,de OR 'ba 34647':ti,ab,kw,de OR 'ba34 647':ti,ab,kw,de OR 'ba34647':ti,ab,kw,de OR 'backen':ti,ab,kw,de OR 'baclan':ti,ab,kw,de OR 'baclapone':ti,ab,kw,de OR 'baclaxiro':ti,ab,kw,de OR 'baclo':ti,ab,kw,de OR 'baclocur':ti,ab,kw,de OR 'baclofene':ti,ab,kw,de OR 'baclon':ti,ab,kw,de OR 'baclopar':ti,ab,kw,de OR 'baclophen':ti,ab,kw,de OR 'baclosal':ti,ab,kw,de OR 'bacofen':ti,ab,kw,de OR 'bacron':ti,ab,kw,de OR 'bafen':ti,ab,kw,de OR 'baklofen':ti,ab,kw,de OR 'baropan':ti,ab,kw,de OR 'beta (4 chlorophenyl) 4 aminobutyric acid':ti,ab,kw,de OR 'beta (4 chlorophenyl) gamma aminobutyric acid':ti,ab,kw,de OR 'beta (aminomethyl) 4 chlorobenzenepropanoic acid':ti,ab,kw,de OR 'beta (aminomethyl) 4 chlorocinnamic acid':ti,ab,kw,de OR 'beta (aminomethyl) 4 chlorohydrocinnamic acid':ti,ab,kw,de OR 'beta (aminomethyl) para chlorocinnamic acid':ti,ab,kw,de OR 'beta (para chlorophenyl) 4 aminobutyric acid':ti,ab,kw,de OR 'beta 4 chlorophenyl 4 aminobutanoic acid':ti,ab,kw,de OR 'beta 4 chlorophenyl gamma aminobutyric acid':ti,ab,kw,de OR 'beta amino methyl chlorohydrocinnamic acid':ti,ab,kw,de OR 'beta aminomethyl para chlorohydrocinnamic acid':ti,ab,kw,de OR 'beta para chlorophenyl gamma aminobutyric acid':ti,ab,kw,de OR 'bigafen':ti,ab,kw,de OR 'ciba 34647 ba':ti,ab,kw,de OR 'ciba 34647ba':ti,ab,kw,de OR 'ciba34647ba':ti,ab,kw,de OR 'colmifen':ti,ab,kw,de OR 'curofen':ti,ab,kw,de OR 'dextro baclofen':ti,ab,kw,de OR 'espast':ti,ab,kw,de OR 'fleqsuvy':ti,ab,kw,de OR 'gabalon':ti,ab,kw,de OR 'gablofen':ti,ab,kw,de OR 'intralcal':ti,ab,kw,de OR 'ipx 056':ti,ab,kw,de OR 'ipx056':ti,ab,kw,de OR 'kemstro':ti,ab,kw,de OR 'lebic':ti,ab,kw,de OR 'levo baclofen':ti,ab,kw,de OR 'liofen':ti,ab,kw,de OR 'lionova':ti,ab,kw,de OR 'lioresal':ti,ab,kw,de OR 'lioresal intrathecal':ti,ab,kw,de OR 'lioresyl':ti,ab,kw,de OR 'liotec':ti,ab,kw,de OR 'lyflex':ti,ab,kw,de OR 'lyvispah':ti,ab,kw,de OR 'miorel (baclofen)':ti,ab,kw,de OR 'neteka':ti,ab,kw,de OR 'onelaxant-r':ti,ab,kw,de OR 'ozobax':ti,ab,kw,de OR 'ozobax ds':ti,ab,kw,de OR 'pacifen':ti,ab,kw,de OR 'spacyr':ti,ab,kw,de OR 'spinax':ti,ab,kw,de OR 'stelax':ti,ab,kw,de OR 'vioridon':ti,ab,kw,de OR 'baclofen':ti,ab,kw,de OR '19583 rp':ti,ab,kw,de OR '2 (3 benzoylphenyl) propionic acid':ti,ab,kw,de OR '2 [3 benzoyl (phenyl)] propionic acid':ti,ab,kw,de OR 'actron (ketoprofen)':ti,ab,kw,de OR 'alreumat':ti,ab,kw,de OR 'alrhemun':ti,ab,kw,de OR 'alrheumat':ti,ab,kw,de OR 'alrheumin':ti,ab,kw,de OR 'alrheumun':ti,ab,kw,de OR 'alrhumat':ti,ab,kw,de OR 'aneol':ti,ab,kw,de OR 'anzema':ti,ab,kw,de OR 'apo-keto':ti,ab,kw,de OR 'arcental':ti,ab,kw,de OR 'begsan':ti,ab,kw,de OR 'bi-profenid':ti,ab,kw,de OR 'bi-profenid lp':ti,ab,kw,de OR 'bi-rofenid':ti,ab,kw,de OR 'biprofenid':ti,ab,kw,de OR 'birofenid':ti,ab,kw,de OR 'capisten':ti,ab,kw,de OR 'cetoprofen':ti,ab,kw,de OR 'diractin':ti,ab,kw,de OR 'dolofar':ti,ab,kw,de OR 'drastirel':ti,ab,kw,de OR 'efiken':ti,ab,kw,de OR 'els m11':ti,ab,kw,de OR 'elsm11':ti,ab,kw,de OR 'epatec':ti,ab,kw,de OR 'extraplus (drug)':ti,ab,kw,de OR 'fastum':ti,ab,kw,de OR 'fetik':ti,ab,kw,de OR 'flexen (ketoprofen)':ti,ab,kw,de OR 'floramil':ti,ab,kw,de OR 'gabrilen':ti,ab,kw,de OR 'gabrilen retard':ti,ab,kw,de OR 'gesicpad k':ti,ab,kw,de OR 'helenil':ti,ab,kw,de OR 'ibifen':ti,ab,kw,de OR 'idea 033':ti,ab,kw,de OR 'idea033':ti,ab,kw,de OR 'impracor':ti,ab,kw,de OR 'ipi 110':ti,ab,kw,de OR 'ipi110':ti,ab,kw,de OR 'iso k (drug)':ti,ab,kw,de OR 'jomezal':ti,ab,kw,de OR 'kaltrofen':ti,ab,kw,de OR 'kebanon':ti,ab,kw,de OR 'keduril':ti,ab,kw,de OR 'kefen':ti,ab,kw,de OR 'kehancer':ti,ab,kw,de OR 'kenhancer':ti,ab,kw,de OR 'kenofen gel':ti,ab,kw,de OR 'keotsan':ti,ab,kw,de OR 'keplat':ti,ab,kw,de OR 'keprofen':ti,ab,kw,de OR 'ketadom':ti,ab,kw,de OR 'ketin':ti,ab,kw,de OR 'keto film':ti,ab,kw,de OR 'ketofarm':ti,ab,kw,de OR 'ketofen':ti,ab,kw,de OR 'ketoflam':ti,ab,kw,de OR 'ketolgin':ti,ab,kw,de OR 'ketolgin gel':ti,ab,kw,de OR 'ketolgin sr':ti,ab,kw,de OR 'ketomex':ti,ab,kw,de OR 'ketonal':ti,ab,kw,de OR 'ketoprofen sodium':ti,ab,kw,de OR 'ketoprofene':ti,ab,kw,de OR 'ketores':ti,ab,kw,de OR 'ketorin':ti,ab,kw,de OR 'ketosolan':ti,ab,kw,de OR 'ketospray':ti,ab,kw,de OR 'ketotransdel':ti,ab,kw,de OR 'ketum':ti,ab,kw,de OR 'knavon':ti,ab,kw,de OR 'kpl 202':ti,ab,kw,de OR 'liotondol':ti,ab,kw,de OR 'lisactiv gola':ti,ab,kw,de OR 'mohrus':ti,ab,kw,de OR 'naxal':ti,ab,kw,de OR 'nexcede':ti,ab,kw,de OR 'novo-keto-ec':ti,ab,kw,de OR 'opokan keto':ti,ab,kw,de OR 'oralcare (drug)':ti,ab,kw,de OR 'orochet':ti,ab,kw,de OR 'orucote':ti,ab,kw,de OR 'orudis':ti,ab,kw,de OR 'orudis e-100':ti,ab,kw,de OR 'orudis ec':ti,ab,kw,de OR 'orudis kt':ti,ab,kw,de OR 'orudis r':ti,ab,kw,de OR 'orudis r-pr':ti,ab,kw,de OR 'orudis retard':ti,ab,kw,de OR 'orudis sr':ti,ab,kw,de OR 'oruvail':ti,ab,kw,de OR 'oruvail ec':ti,ab,kw,de OR 'oruvail sr':ti,ab,kw,de OR 'oscorel':ti,ab,kw,de OR 'ovurila':ti,ab,kw,de OR 'ovurila e':ti,ab,kw,de OR 'oxoprofene':ti,ab,kw,de OR 'phardol':ti,ab,kw,de OR 'profecom':ti,ab,kw,de OR 'profemigr':ti,ab,kw,de OR 'profenid':ti,ab,kw,de OR 'profenid 50':ti,ab,kw,de OR 'profenid lp':ti,ab,kw,de OR 'profika':ti,ab,kw,de OR 'prontoflex':ti,ab,kw,de OR 'protofen':ti,ab,kw,de OR 'provail cr':ti,ab,kw,de OR 'rematof':ti,ab,kw,de OR 'rhetoflam':ti,ab,kw,de OR 'rheuna pap':ti,ab,kw,de OR 'rofenid':ti,ab,kw,de OR 'rofixdol':ti,ab,kw,de OR 'rp 19, 583':ti,ab,kw,de OR 'rp 19583':ti,ab,kw,de OR 'siduro':ti,ab,kw,de OR 'sodium ketoprofen':ti,ab,kw,de OR 'spondylon':ti,ab,kw,de OR 'tdlp 110':ti,ab,kw,de OR 'tdlp110':ti,ab,kw,de OR 'tiloket':ti,ab,kw,de OR 'topofen':ti,ab,kw,de OR 'toprec':ti,ab,kw,de OR 'treosin':ti,ab,kw,de OR 'valket':ti,ab,kw,de OR 'yerasel':ti,ab,kw,de OR 'ketoprofen':ti,ab,kw,de OR '(2, 6 dichlorophenylamine) 2 imidazoline hydrochloride':ti,ab,kw,de OR '2 (2, 6 dichloranilino) 2 imidazoline':ti,ab,kw,de OR '2 (2, 6 dichloroanilino) 2 imidazoline':ti,ab,kw,de OR '2 (2, 6 dichloroanilino) 2 imidazoline hydrochloride':ti,ab,kw,de OR '2 (2, 6 dichloroanilino) imidazoline':ti,ab,kw,de OR '2 (2, 6 dichlorophenyl 1 amino) 2 imidazoline':ti,ab,kw,de OR '2 (2, 6 dichlorophenylamino) 2 imidazoline':ti,ab,kw,de OR '2 (2, 6 dichlorophenylamino) 2 imidazoline hydrochloride':ti,ab,kw,de OR '2 (2, 6 dichlorophenylamino) imidazoline':ti,ab,kw,de OR '2 [ (2, 6 dichlorophenyl) imino] imidazoline':ti,ab,kw,de OR '2 [2, 6 dichlorphenylamino] 2 imidazoline hydrochloride':ti,ab,kw,de OR '2, 6 dichloro n 2 imidazolidinylidenebenzenamine':ti,ab,kw,de OR '2, 6 dichloro n imidazolidin 2 ylideneaniline':ti,ab,kw,de OR '2, 6 dichlorophenylamine 2 imidazoline hydrochloride':ti,ab,kw,de OR 'adesipress tts 2':ti,ab,kw,de OR 'adesipress tts2':ti,ab,kw,de OR 'arkamin':ti,ab,kw,de OR 'atensina':ti,ab,kw,de OR 'calotep':ti,ab,kw,de OR 'caprysin':ti,ab,kw,de OR 'catapres':ti,ab,kw,de OR 'catapres tts':ti,ab,kw,de OR 'catapres-tts-1':ti,ab,kw,de OR 'catapres-tts-2':ti,ab,kw,de OR 'catapres-tts-3':ti,ab,kw,de OR 'catapresan':ti,ab,kw,de OR 'catapresan 100':ti,ab,kw,de OR 'catapresan depot':ti,ab,kw,de OR 'catapresan tts':ti,ab,kw,de OR 'catapressan':ti,ab,kw,de OR 'catapressan perlonguettes':ti,ab,kw,de OR 'catapressant':ti,ab,kw,de OR 'catasan':ti,ab,kw,de OR 'chlofazolin':ti,ab,kw,de OR 'chlophazolin':ti,ab,kw,de OR 'chlophelin':ti,ab,kw,de OR 'clinidine':ti,ab,kw,de OR 'clofelin':ti,ab,kw,de OR 'clofeline':ti,ab,kw,de OR 'clomidine':ti,ab,kw,de OR 'clondine':ti,ab,kw,de OR 'clonicel':ti,ab,kw,de OR 'clonidin':ti,ab,kw,de OR 'clonidine chlorhydrate':ti,ab,kw,de OR 'clonidine hydrochloride':ti,ab,kw,de OR 'clonidine tts-1':ti,ab,kw,de OR 'clonidine tts-2':ti,ab,kw,de OR 'clonidine tts-3':ti,ab,kw,de OR 'clonipresan':ti,ab,kw,de OR 'clonistada':ti,ab,kw,de OR 'clonistada retard':ti,ab,kw,de OR 'clonnirit':ti,ab,kw,de OR 'clophelin':ti,ab,kw,de OR 'clopheline':ti,ab,kw,de OR 'daipres':ti,ab,kw,de OR 'dcai':ti,ab,kw,de OR 'dichlorophenylaminoimidazoline':ti,ab,kw,de OR 'dichlorophenylaminoimidazoline hydrochloride':ti,ab,kw,de OR 'dixarit':ti,ab,kw,de OR 'duraclon':ti,ab,kw,de OR 'haemiton':ti,ab,kw,de OR 'hemiton':ti,ab,kw,de OR 'huma-clonidine':ti,ab,kw,de OR 'hypodine':ti,ab,kw,de OR 'isoglaucon':ti,ab,kw,de OR 'jenloga':ti,ab,kw,de OR 'kapvay':ti,ab,kw,de OR 'm 5041t':ti,ab,kw,de OR 'm5041t':ti,ab,kw,de OR 'melzin':ti,ab,kw,de OR 'n (2, 6 dichlorophenyl) 4, 5 dihydro 1h imidazol 2 amine':ti,ab,kw,de OR 'n (2, 6 dichlorophenyl) 4, 5 dihydro 2 imidazolamine':ti,ab,kw,de OR 'n (2, 6 dichlorophenyl) 4, 5 dihydroimidazol 2 amine':ti,ab,kw,de OR 'nexiclon':ti,ab,kw,de OR 'nexiclon xr':ti,ab,kw,de OR 'normopresan':ti,ab,kw,de OR 'normopresin':ti,ab,kw,de OR 'onyda':ti,ab,kw,de OR 'onyda xr':ti,ab,kw,de OR 'paracefan':ti,ab,kw,de OR 'st 155':ti,ab,kw,de OR 'st155':ti,ab,kw,de OR 'sulmidine':ti,ab,kw,de OR 'taitecin':ti,ab,kw,de OR 'tenso timelets':ti,ab,kw,de OR 'clonidine':ti,ab,kw,de OR '1 (aminomethyl) cyclohexaneacetic acid':ti,ab,kw,de OR '2 [1 (aminomethyl) cyclohexyl] acetic acid':ti,ab,kw,de OR 'ci 945':ti,ab,kw,de OR 'ci945':ti,ab,kw,de OR 'dineurin':ti,ab,kw,de OR 'dm 1796':ti,ab,kw,de OR 'dm 5689':ti,ab,kw,de OR 'dm1796':ti,ab,kw,de OR 'dm5689':ti,ab,kw,de OR 'gabalept':ti,ab,kw,de OR 'gabaliquid geriasan':ti,ab,kw,de OR 'gabapen':ti,ab,kw,de OR 'gabatin':ti,ab,kw,de OR 'gantin':ti,ab,kw,de OR 'go 3450':ti,ab,kw,de OR 'go3450':ti,ab,kw,de OR 'goe 3450':ti,ab,kw,de OR 'goe3450':ti,ab,kw,de OR 'gralise':ti,ab,kw,de OR 'kaptin':ti,ab,kw,de OR 'keneil':ti,ab,kw,de OR 'neurontin':ti,ab,kw,de OR 'neurotonin':ti,ab,kw,de OR 'nupentin':ti,ab,kw,de OR 'sefelsa':ti,ab,kw,de OR 'serada':ti,ab,kw,de OR 'gabapentin':ti,ab,kw,de OR corticosteroid*:ti,ab,kw,de OR menthol:ti,ab,kw,de OR levomenthol:ti,ab,kw,de OR 'peppermint oil':ti,ab,kw,de OR 'clove oil':ti,ab,kw,de OR camphor:ti,ab,kw,de OR capsaicin:ti,ab,kw,de OR 'cajuput oil':ti,ab,kw,de OR cineole:ti,ab,kw,de OR eucalyptol:ti,ab,kw,de OR 'ben gay':ti,ab,kw,de OR bengay:ti,ab,kw,de OR 'icy hot':ti,ab,kw,de OR icyhot:ti,ab,kw,de |  |
| #9 | paracetamol'/exp OR 'nonsteroid antiinflammatory agent'/exp OR 'prostaglandin synthase inhibi-tor' OR 'cyclooxygenase 2 inhibitor'/exp OR 'local anesthetic agent'/exp OR 'corticosteroid'/exp OR 'gabapentin'/exp OR 'pregabalin'/exp OR 'ketamine'/exp OR 'alpha 2 adrenergic receptor stimulating agent'/exp OR 'clonidine'/exp OR 'dexmedetomidine'/exp OR 'lidocaine'/exp OR 'muscle relaxant agent'/exp OR 'diclofenac'/exp | 3235006 |
| #10 | 'analgesic agent'/exp/dd_tp,dd_td | 14115 |
| #11 | topical drug administration'/de OR 'cutaneous drug administration'/de OR 'transdermal drug administration'/de OR 'ointment'/exp OR 'cream'/exp OR 'gel'/exp OR 'liniment'/exp OR 'lotion'/exp OR 'salve'/exp OR 'transdermal patch'/exp OR topical*:ti,ab,kw OR transdermal*:ti,ab,kw OR 'trans dermal*':ti,ab,kw OR ointment*:ti,ab,kw OR cream*:ti,ab,kw OR 'gel':ti,ab,kw OR 'gels':ti,ab,kw OR liniment*:ti,ab,kw | 794961 |
| #12 | topical analgesi*' OR 'topical pain relie*' OR 'transdermal analgesi*' OR 'transdermal pain relie*' OR 'trans dermal analgesi*' OR 'trans dermal pain relie*' OR 'transcutaneous analgesi*' OR 'transcutaneous pain relie*' OR 'trans cutaneous analgesi*' OR 'trans cutaneous pain relie*' | 995 |
| #13 | #1 OR #2 OR #3 | 398989 |
| #14 | #5 OR #6 | 972249 |
| #15 | #13 AND #14 | 50348 |
| #16 | #4 OR #15 | 108374 |
| #17 | #7 OR #8 OR #9 | 3386561 |
| #18 | #10 OR #11 OR #12 | 802160 |
| #19 | #17 AND #18 | 141829 |
| #20 | #16 AND #19 | 755 |
| #21 | #20 NOT ([animals]/lim NOT [humans]/lim) | 721 |
| #22 | #20 NOT ([animals]/lim NOT [humans]/lim) AND ([cochrane review]/lim OR [systematic review]/lim OR [meta analysis]/lim) | 43 |
| #23 | #21 NOT ('conference review'/it OR 'editorial'/it OR 'letter'/it OR 'note'/it OR 'review'/it OR 'short survey'/it OR 'tombstone'/it OR 'case report'/de OR 'meta analysis'/de OR 'meta analysis topic'/de OR 'systematic review'/de OR 'systematic review topic'/de) | 423 |

MEDLINE (Ovid MEDLINE(R) ALL <1946 to May 29, 2025>)

| No. | MEDLINE Query | Results |
| --- | --- | --- |
| 1 | Sports Medicine/ or "Physical and Rehabilitation Medicine"/ or "Rehabilitation"/ or 'sport medicine'.mp. or 'sport physiology'.mp. or 'sports physiology'.mp. or 'sports medicine'.mp. or 'rehabilitation science'.mp. or 'rehabilitation medicine'.mp. [mp=title, book title, abstract, original title, name of substance word, subject heading word, floating sub-heading word, keyword heading word, organism supplementary concept word, protocol supplementary concept word, rare disease supplementary concept word, unique identifier, synonyms, population supplementary concept word, anatomy supplementary concept word] | 44881 |
| 2 | exp "Athletic Injuries"/ or exp "Return to Sport"/ or 'athlete injury'.mp. or 'athlete trauma'.mp. or 'athletic injuries'.mp. or 'athletic injury'.mp. or 'athletic trauma'.mp. or 'injury, athletic'.mp. or 'sport accident'.mp. or 'sport related injury'.mp. or 'sport trauma'.mp. or 'sportrelated injury'.mp. or 'sports injury'.mp. or 'sports related injury'.mp. or 'sports trauma'.mp. or 'sportsrelated injury'.mp. or 'sport injury'.mp. or 're-sumption of sport'.mp. or 'resumption of sporting activities'.mp. or 'return to play'.mp. or 'return to playing sport'.mp. or 'return to sporting activities'.mp. or 'return to sport'.mp. [mp=title, book title, abstract, original title, name of substance word, subject heading word, floating sub-heading word, keyword heading word, organism supplementary concept word, protocol supplementary concept word, rare disease supplementary concept word, unique identifier, synonyms, population supplementary concept word, anatomy supplementary concept word] | 41567 |
| 3 | exp "Athletes"/ or "Sports"/ or "Baseball"/ or "Basketball"/ or "Bicycling"/ or "Boxing"/ or "Cricket Sport"/ or "Football"/ or "Gaelic Football"/ or "Golf"/ or "Gymnastics"/ or "Hockey"/ or "Martial Arts"/ or "Mountaineering"/ or "Racquet Sports"/ or "Tennis"/ or "Rugby"/ or exp "Running"/ or "Skating"/ or exp "Snow Sports"/ or "Soccer"/ or exp "Team Sports"/ or "Track and Field"/ or "Volleyball"/ or "Nordic Walking"/ or exp "Water Sports"/ or "Weight Lifting"/ or "Wrestling"/ or "Youth Sports"/ or (Sport* or athlet*).ti,ab. | 258178 |
| 4 | exp "Tennis Elbow"/ or exp "Elbow Tendinopathy"/ or (exp "Achilles Tendon"/ and exp "Tendinopathy"/) or exp "Anterior Cruciate Ligament Injuries"/ or exp "Patellofemoral Pain Syndrome"/ or exp "Bursitis"/ or exp "Rotator Cuff Injuries"/ or (exp "Collateral Ligament, Ulnar"/ and "Sprains OR Strains"/) or exp "Tibial Meniscus Injuries"/ or "Ankle Injuries"/ or (exp "Lateral Ligament, Ankle"/ and "Sprains OR Strains"/) or (exp "Hamstring Muscles"/ and "Sprains OR Strains"/) or (exp "Hamstring Tendons"/ and exp "Tendinopathy"/) or 'bursitis, radiohumeral'.mp. or 'elbow, tennis'.mp. or 'epicondylatgia humeri'.mp. or 'epicondylitis humeri'.mp. or 'epicondylitis lateralis'.mp. or 'epicondylitis, external humeral'.mp. or 'epicondylitis, radiohumeral'.mp. or 'epycondylitis humeri'.mp. or 'external humeral epicondylitis'.mp. or 'humeral epicondylitis'.mp. or 'humerus epicondylitis'.mp. or 'lateral elbow tendinopathy'.mp. or 'lateral elbow tendinosis'.mp. or 'lateral elbow tendonopathy'.mp. or 'lateral epicondylitis'.mp. or 'lateral humeral epicondylitis'.mp. or 'radiohumeral bursitis'.mp. or 'radiohumeral epicondylitis'.mp. or 'tennis arm'.mp. or 'tennis elbow'.mp. or 'epicondylitis medialis'.mp. or 'golfer arm'.mp. or 'golfer elbow'.mp. or 'medial elbow tendinopathy'.mp. or 'medial elbow tendinosis'.mp. or 'medial humeral epicondylitis'.mp. or 'medial epicondylitis'.mp. or 'anterior cruciate ligament injuries'.mp. or 'anterior cruciate ligament injury'.mp. or 'anterior cruciate knee ligament rupture'.mp. or 'rupture, anterior crucial ligament'.mp. or 'anterior cruciate ligament rupture'.mp. or 'achilles tendinopathy'.mp. or 'achilles tendinosis'.mp. or 'achilles tendonopathy'.mp. or 'achilles tendinitis'.mp. or 'patella femoral pain syndrome'.mp. or 'patellar femoral pain syndrome'.mp. or 'patello-femoral pain syndrome'.mp. or 'patello-femoral stress syndrome'.mp. or 'patello-femoral syndrome'.mp. or 'patellofemoral stress syndrome'.mp. or 'patellofemoral syndrome'.mp. or 'patellofemoral pain syndrome'.mp. or 'bursitis'.mp. or 'calcaneal bursitis'.mp. or 'capped hock'.mp. or 'heel bursitis'.mp. or 'retrocalcaneal bursitis'.mp. or 'infection in the bursa'.mp. or 'infectious bursitis'.mp. or 'infective bursitis'.mp. or 'septic bursitis'.mp. or 'shoulder bursitis'.mp. or 'shoulders bursitis'.mp. or 'subacromial-subdeltoid bursitis'.mp. or 'subdeltoid bursitis'.mp. or 'subacromial bursitis'.mp. or 'rotator cuff impingement'.mp. or 'rotator cuff syndrome'.mp. or 'rotator cuff tendinitis'.mp. or 'rotator cuff tendonitis'.mp. or 'shoulder cuff syndrome'.mp. or 'shoulder impingement'.mp. or 'subacromial impingement'.mp. or 'subacromial impingement syndrome'.mp. or 'syndrome, shoulder impingement'.mp. or 'shoulder impingement syndrome'.mp. or 'cuff injury'.mp. or 'cuff lesion'.mp. or 'rotator cuff disease'.mp. or 'rotator cuff disorder'.mp. or 'rotator cuff disorders'.mp. or 'rotator cuff injuries'.mp. or 'rotator cuff lesion'.mp. or 'rotator cuff lesions'.mp. or 'rotator cuff pathology'.mp. or 'rotator cuff tendinopathies'.mp. or 'rotator cuff tendinopathy'.mp. or 'shoulder rotator cuff degeneration'.mp. or 'shoulder rotator cuff lesion'.mp. or 'rotator cuff injury'.mp. or 'quadrangular space syndrome'.mp. or 'quadrilateral space syndrome'.mp. or 'rotator cuff ruptures'.mp. or 'rotator cuff tear'.mp. or 'rotator cuff tears'.mp. or 'rotatory cuff rupture'.mp. or 'shoulder rotator cuff rupture'.mp. or 'shoulder rotator cuff tear'.mp. or 'rotator cuff rupture'.mp. or 'apatite-associated destructive arthritis'.mp. or 'cuff tear arthropathy'.mp. or 'Milwaukee shoulder'.mp. or 'Milwaukee shoulder syndrome'.mp. or 'rotator cuff tear arthropathy'.mp. or (('collateral ligament, ulnar' or 'ligamentum collaterale ulnare' or 'ulna collateral ligament' or 'ulnar collateral ligament') and ('ligament injuries' or 'ligament trauma' or 'ligament injury' or 'ligament rupture')).mp. or 'fracture, meniscus'.mp. or 'knee meniscal rupture'.mp. or 'knee meniscus injury'.mp. or 'knee meniscus lesion'.mp. or 'knee meniscus trauma'.mp. or 'lesion, meniscus'.mp. or 'meniscal fracture'.mp. or 'meniscal lesion'.mp. or 'meniscal lesions'.mp. or 'meniscal rupture, knee'.mp. or 'meniscopathy'.mp. or 'meniscus damage'.mp. or 'meniscus fracture'.mp. or 'meniscus injury'.mp. or 'meniscus lesion'.mp. or 'meniscus rupture, knee'.mp. or 'meniscus tear'.mp. or 'meniscus trauma'.mp. or 'rupture, meniscus'.mp. or 'Tibial Meniscus Injuries'.mp. or 'torn meniscus'.mp. or 'trauma, meniscus'.mp. or 'knee meniscus rupture'.mp. or 'ankle joint sprain'.mp. or 'ankle ligament sprain'.mp. or 'sprain, ankle'.mp. or 'sprained ankle'.mp. or 'ankle sprain'.mp. or (('ankle ligament, lateral' or 'lateral ligament, ankle' or 'ligament, ankle, lateral' or 'ankle lateral ligament') and ('ligament injuries' or 'ligament trauma' or 'ligament injury' or 'ligament rupture')).mp. or (Hip-flexor* and (strain* or trauma* or injur*)).mp. or Groin-pull*.mp. or pulled-groin*.mp. or groin-injur*.mp. or groin-strain*.mp. or hamstring-pull*.mp. or pulled-hamstring*.mp. or hamstring-injur*.mp. or hamstring-strain*.mp. [mp=title, book title, abstract, original title, name of substance word, subject heading word, floating sub-heading word, keyword heading word, organism supplementary concept word, protocol supplementary concept word, rare disease supplementary concept word, unique identifier, synonyms, population supplementary concept word, anatomy supplementary concept word] | 66359 |
| 5 | Arthralgia/ or "Metatarsalgia"/ or "Musculoskeletal Pain"/ or "Myalgia"/ or exp "Elbow Injuries"/ or "Medial Tibial Stress Syndrome"/ or exp "Foot Injuries"/ or exp "Knee Injuries"/ or exp "Tendon Injuries"/ or "Sprains and Strains"/ or exp "Ankle Injuries"/ or 'arthrodynia'.mp. or 'joint ache'.mp. or 'joint pain'.mp. or 'arthralgia'.mp. or myalgia.mp. or muscle-pain*.mp. or muscle-soreness*.mp. or Joint-injur*.mp. or Ligament-injur*.mp. or tendon-injur*.mp. or shin-splint*.mp. or sprain*.mp. or muscle-strain*.mp. or muscle-injur*.mp. or muscular-pain*.mp. or muscular-sore*.mp. or muscular-injur*.mp. or strained-muscle*.mp. or injured-muscle*.mp. or sore-muscle*.mp. [mp=title, book title, abstract, original title, name of substance word, subject heading word, floating sub-heading word, keyword heading word, organism supplementary concept word, protocol supplementary concept word, rare disease supplementary concept word, unique identifier, synonyms, population supplementary concept word, anatomy supplementary concept word] | 154440 |
| 6 | exp "Analgesia"/ or analgesi*.mp. or (pain* adj4 (relie* or mitigat* or reduc* or manag* or control*)).mp. | 404927 |
| 7 | ('lidocaine' or 'betacaine' or 'xylocaine' or 'salicylic acid methyl ester' or 'betula oil' or 'gaultheria oil' or 'methyl salicylate' or 'methylsalicylate' or 'oil of wintergreen' or 'sweet birch oil' or 'wintergreen oil' or 'Hydroxyethyl-salicylate' or 'ethyl glycol salicylate' or 'ethylene glycol salicylate' or Ethyl-salicylate or Tetrahydrofurfuryl-salicylate or Diethylamine-salicylate or Acetylsalicylic-acid or Salicylic-acid or 'methyl nicotinate' or 'methylnicotinate' or 'midalgan' or 'nicotinic acid methyl ester' or 'hexyl nicotinate' or 'hexylnicotinate' or 'nicotinic acid hexyl ester' or 'benzylnicotinate' or 'nicotinic acid benzyl ester' or 'rubriment' or 'benzyl nicotinate' or 'nicoboxil' or 'diclofenac' or 'almiral gel' or 'berafen gel' or 'berifen gel' or 'ddl plaster' or 'depain plaster' or 'difenol gel' or 'dolaut gel' or 'dolotren gel' or 'inac gel' or 'jonac gel' or 'leviogel' or 'olfen gel' or 'olfen roll-on' or 'relaxyl gel' or 'remethan gel' or 'renvol emulgel' or 'solaraze' or 'vartelon gel' or 'voldic emulgel' or 'voltaren' or Ibuprofen or Naproxen or 'etofenamate' or 'phenylbutazone' or 'fluocinolone acetonide' or Piroxicam or 'amitriptyline' or 'ketoprofen' or 'clonidine' or 'gabapentin' or Corticosteroid* or Menthol or Levomenthol or Peppermint-oil or Clove-oil or camphor or capsaicin or Cajuput-oil or Cineole or eucalyptol or ben-gay or bengay or icy-hot or icyhot).mp. [mp=title, book title, abstract, original title, name of substance word, subject heading word, floating sub-heading word, keyword heading word, organism supplementary concept word, protocol supplementary concept word, rare disease supplementary concept word, unique identifier, synonyms, population supplementary concept word, anatomy supplementary concept word] | 319535 |
| 8 | exp "Acetaminophen"/ or exp "Anti-Inflammatory Agents, Non-Steroidal"/ or exp "Anesthetics, Local"/ or exp "Adrenal Cortex Hormones"/ or exp "Gabapentin"/ or exp "Pregabalin"/ or exp "Ketamine"/ or exp "Adrenergic alpha-2 Receptor Agonists"/ or exp "Clonidine"/ or exp "Dexmedetomidine"/ or exp "Lidocaine"/ or exp "Neuromuscular Blocking Agents"/ or exp "Diclofenac"/ | 843667 |
| 9 | Administration, Topical/ or exp "Administration, Cutaneous"/ or exp "Transdermal Patch"/ or exp "Skin Cream"/ or exp "Gels"/ or exp "Ointments"/ or exp "Liniments"/ or (Topical* or transdermal* or trans-dermal* or patch* or ointment* or cream* or "gel" or "gels" or Liniment*).mp. | 920965 |
| 10 | (topical-analgesi* or topical-pain-relie* or transdermal-analgesi* or transdermal-pain-relie* or trans-dermal-analgesi* or trans-dermal-pain-relie* or transcutaneous-analgesi* or transcutaneous-pain-relie* or trans-cutaneous-analgesi* or trans-cutaneous-pain-relie*).mp. | 594 |
| 11 | 1 or 2 or 3 | 293036 |
| 12 | 5 or 6 | 547755 |
| 13 | 11 and 12 | 29527 |
| 14 | 4 or 13 | 83476 |
| 15 | 7 or 8 | 998505 |
| 16 | 9 or 10 | 920967 |
| 17 | 15 and 16 | 62528 |
| 18 | 14 and 17 | 245 |
| 19 | 18 not (exp "animals"/ not exp "humans"/) | 236 |
| 20 | 19 not ("case reports" or "comment" or "editorial" or "guideline" or "introductory journal article" or "meta analysis" or "news" or "retracted publication" or "review" or "systematic review").pt. | 149 |

Cochrane Central Register of Controlled Trials (CochraneLibrary.com platform, Wiley, Issue 4 of 12, April 2025)

| No. | Cochrane CENTRAL Query | Results |
| --- | --- | --- |
| #1 | [mh ^"Sports Medicine"] or [mh ^"Physical and Rehabilitation Medicine"] or [mh ^"Rehabilitation"] or ('sport medicine' or 'sport physiology' or 'sports physiology' or 'sports medicine' or 'rehabilitation science' or 'rehabilitation medicine') | 46879 |
| #2 | [mh "Athletic Injuries"] or [mh "Return to Sport"] or (athlete injury' or 'athlete trauma' or 'athletic injuries' or 'athletic injury' or 'athletic trauma' or 'injury, athletic' or 'sport accident' or 'sport related injury' or 'sport trauma' or 'sportrelated injury' or 'sports injury' or 'sports related injury' or 'sports trauma' or 'sportsrelated injury' or 'sport injury' or 're-sumption of sport' or 'resumption of sporting activities' or 'return to play' or 'return to playing sport' or 'return to sporting activities' or 'return to sport') | 7770 |
| #3 | [mh "Athletes"] or [mh ^"Sports"] or [mh ^"Baseball"] or [mh ^"Basketball"] or [mh ^"Bicycling"] or [mh ^"Boxing"] or [mh ^"Cricket Sport"] or [mh ^"Football"] or [mh ^"Gaelic Football"] or [mh ^"Golf"] or [mh ^"Gymnastics"] or [mh ^"Hockey"] or [mh ^"Martial Arts"] or [mh ^"Mountaineering"] or [mh ^"Racquet Sports"] or [mh ^"Tennis"] or [mh ^"Rugby"] or [mh "Running"] or [mh ^"Skating"] or [mh "Snow Sports"] or [mh ^"Soccer"] or [mh "Team Sports"] or [mh ^"Track and Field"] or [mh ^"Volleyball"] or [mh ^"Nordic Walking"] or [mh "Water Sports"] or [mh ^"Weight Lifting"] or [mh ^"Wrestling"] or [mh ^"Youth Sports"] or (Sport* or athlet*) | 47217 |
| #4 | [mh "Tennis Elbow"] or [mh "Elbow Tendinopathy"] or ([mh "Achilles Tendon"] and [mh "Tendinopathy"]) or [mh "Anterior Cruciate Ligament Injuries"] or [mh "Patellofemoral Pain Syndrome"] or [mh "Bursitis"] or [mh "Rotator Cuff Injuries"] or ([mh "Collateral Ligament, Ulnar"] and [mh ^"Sprains OR Strains"]) or [mh "Tibial Meniscus Injuries"] or [mh ^"Ankle Injuries"] or ([mh "Lateral Ligament, Ankle"] and [mh ^"Sprains OR Strains"]) or ([mh "Hamstring Muscles"] and [mh ^"Sprains OR Strains"]) or ([mh "Hamstring Tendons"] and [mh "Tendinopathy"]) or (epicondylatgia-humeri* OR epicondylitis-humeri* OR epicondylitis-lateralis* OR humeral-epicondylitis* OR humerus-epicondylitis* OR lateral-elbow-tendinopathy* OR lateral-elbow-tendinosis* OR lateral-elbow-tendonopathy* OR lateral-epicondylitis* OR lateral-humeral-epicondylitis* OR radiohumeral-bursitis* OR radiohumeral-epicondylitis* OR tennis-arm* OR tennis-elbow* OR epicondylitis-medialis* OR golfer-arm* OR golfer-elbow* OR medial-elbow-tendinopathy* OR medial-elbow-tendinosis* OR medial-humeral-epicondylitis* OR medial-epicondylitis* OR anterior-cruciate-ligament-injuries* OR anterior-cruciate-ligament-injury* OR anterior-cruciate-knee-ligament-rupture* OR anterior-cruciate-ligament-rupture* OR achilles-tendinopathy* OR achilles-tendinosis* OR achilles-tendonopathy* OR achilles-tendinitis* OR patella-femoral-pain-syndrome* OR patellar-femoral-pain-syndrome* OR patello-femoral-pain-syndrome* OR patello-femoral-stress-syndrome* OR patello-femoral-syndrome* OR patellofemoral-stress-syndrome* OR patellofemoral-syndrome* OR patellofemoral-pain-syndrome* OR bursitis* OR capped-hock* OR shoulder-bursitis* OR subdeltoid-bursitis* OR subacromial-bursitis* OR rotator-cuff-impingement* OR rotator-cuff-syndrome* OR rotator-cuff-tendinitis* OR rotator-cuff-tendonitis* OR shoulder-cuff-syndrome* OR shoulder-impingement* OR subacromial-impingement* OR subacromial-impingement-syndrome* OR shoulder-impingement-syndrome* OR rotator-cuff-disease* OR rotator-cuff-disorder* OR rotator-cuff-disorders* OR rotator-cuff-injuries* OR rotator-cuff-lesion* OR rotator-cuff-lesions* OR rotator-cuff-pathology* OR rotator-cuff-tendinopathies* OR rotator-cuff-tendinopathy* OR rotator-cuff-injury* OR quadrangular-space-syndrome* OR quadrilateral-space-syndrome* OR rotator-cuff-ruptures* OR rotator-cuff-tear* OR rotator-cuff-tears* OR rotatory-cuff-rupture* OR rotator-cuff-rupture* OR cuff-tear-arthropathy* OR Milwaukee-shoulder* OR Milwaukee-shoulder-syndrome* OR rotator-cuff-tear-arthropathy OR ((collateral-ligament-ulnar* OR ligamentum-collaterale-ulnare* OR ulna-collateral-ligament* OR ulnar-collateral-ligament) AND (ligament-injuries* OR ligament-trauma* OR ligament-injury* OR ligament-rupture)) OR knee-meniscal-rupture* OR knee-meniscus-injury* OR knee-meniscus-lesion* OR knee-meniscus-trauma* OR meniscal-fracture* OR meniscal-lesion* OR meniscal-lesions* OR meniscopathy* OR meniscus-damage* OR meniscus-fracture* OR meniscus-injury* OR meniscus-lesion* OR meniscus-tear* OR meniscus-trauma* OR Tibial-Meniscus-Injuries* OR torn-meniscus* OR knee-meniscus-rupture* OR ankle-joint-sprain* OR ankle-ligament-sprain* OR sprained-ankle* OR ankle-sprain* OR ((ankle-ligament-lateral* OR lateral-ligament-ankle* OR ligament-ankle-lateral* OR ankle-lateral-ligament) AND (ligament-injuries* OR ligament-trauma* OR ligament-injury* OR ligament-rupture)) OR (Hip-flexor* AND (strain* OR trauma* OR injur*)) OR Groin-pull* OR pulled-groin* OR groin-injur* OR groin-strain* OR hamstring-pull* OR pulled-hamstring* OR hamstring-injur* OR hamstring-strain*) | 11218 |
| #5 | [mh ^"Arthralgia"] or [mh ^"Metatarsalgia"] or [mh ^"Musculoskeletal Pain"] or [mh ^"Myalgia"] or [mh "Elbow Injuries"] or [mh ^"Medial Tibial Stress Syndrome"] or [mh "Foot Injuries"] or [mh "Knee Injuries"] or [mh "Tendon Injuries"] or [mh ^"Sprains and Strains"] or [mh "Ankle Injuries"] or ('arthrodynia' or 'joint ache' or 'joint pain' or 'arthralgia' or myalgia or muscle-pain* or muscle-soreness* or Joint-injur* or Ligament-injur* or tendon-injur* or shin-splint* or sprain* or muscle-strain* or muscle-injur* or muscular-pain* or muscular-sore* or muscular-injur* or strained-muscle* or injured-muscle* or sore-muscle*) | 46324 |
| #6 | [mh "Analgesia"] or analgesi* or (pain* NEAR/3 (relie* or mitigat* or reduc* or manag* or control*)) | 151034 |
| #7 | ('lidocaine' or 'betacaine' or 'xylocaine' or 'salicylic acid methyl ester' or 'betula oil' or 'gaultheria oil' or 'methyl salicylate' or 'methylsalicylate' or 'oil of wintergreen' or 'sweet birch oil' or 'wintergreen oil' or 'Hydroxyethyl-salicylate' or 'ethyl glycol salicylate' or 'ethylene glycol salicylate' or Ethyl-salicylate or Tetrahydrofurfuryl-salicylate or Diethylamine-salicylate or Acetylsalicylic-acid or Salicylic-acid or 'methyl nicotinate' or 'methylnicotinate' or 'midalgan' or 'nicotinic acid methyl ester' or 'hexyl nicotinate' or 'hexylnicotinate' or 'nicotinic acid hexyl ester' or 'benzylnicotinate' or 'nicotinic acid benzyl ester' or 'rubriment' or 'benzyl nicotinate' or 'nicoboxil' or 'diclofenac' or 'almiral gel' or 'berafen gel' or 'berifen gel' or 'ddl plaster' or 'depain plaster' or 'difenol gel' or 'dolaut gel' or 'dolotren gel' or 'inac gel' or 'jonac gel' or 'leviogel' or 'olfen gel' or 'olfen roll-on' or 'relaxyl gel' or 'remethan gel' or 'renvol emulgel' or 'solaraze' or 'vartelon gel' or 'voldic emulgel' or 'voltaren' or Ibuprofen or Naproxen or 'etofenamate' or 'phenylbutazone' or 'fluocinolone acetonide' or Piroxicam or 'amitriptyline' or 'ketoprofen' or 'clonidine' or 'gabapentin' or Corticosteroid* or Menthol or Levomenthol or Peppermint-oil or Clove-oil or camphor or capsaicin or Cajuput-oil or Cineole or eucalyptol or ben-gay or bengay or icy-hot or icyhot) | 79549 |
| #8 | [mh "Acetaminophen"] or [mh "Anti-Inflammatory Agents, Non-Steroidal"] or [mh "Anesthetics, Local"] or [mh "Adrenal Cortex Hormones"] or [mh "Gabapentin"] or [mh "Pregabalin"] or [mh "Ketamine"] or [mh "Adrenergic alpha-2 Receptor Agonists"] or [mh "Clonidine"] or [mh "Dexmedetomidine"] or [mh "Lidocaine"] or [mh "Neuromuscular Blocking Agents"] or [mh "Diclofenac"] | 56569 |
| #9 | [mh ^"Administration, Topical"] or [mh "Administration, Cutaneous"] or [mh "Transdermal Patch"] or [mh "Skin Cream"] or [mh "Gels"] or [mh "Ointments"] or [mh "Liniments"] or Topical* or transdermal* or trans-dermal* or patch* or ointment* or cream* or "gel" or "gels" or Liniment* | 78879 |
| #10 | (topical-analgesi* or topical-pain-relie* or transdermal-analgesi* or transdermal-pain-relie* or trans-dermal-analgesi* or trans-dermal-pain-relie* or transcutaneous-analgesi* or transcutaneous-pain-relie* or trans-cutaneous-analgesi* or trans-cutaneous-pain-relie*) | 490 |
| #11 | #1 or #2 or #3 | 71275 |
| #12 | #5 or #6 | 183687 |
| #13 | #11 and #12 | 12773 |
| #14 | #4 or #13 | 20631 |
| #15 | #7 or #8 | 113368 |
| #16 | #9 or #10 | 78879 |
| #17 | #15 and #16 | 13974 |
| #18 | #14 and #17 | 405 |
| #19 | #18 in Trials | 287 |

Web of Science Core Collection (Web of Science Platform, Clarivate, Editions = Arts & Humanities Citation Index, Emerging Sources Citation Index [previous 5 years], Conference Proceedings Citation Index, Science Citation Index-EXPANDED , and Social Science Citation Index)

| No. | Web of Science Query | Results |
| --- | --- | --- |
| 1 | TI=('sport medicine' OR 'sport physiology' OR 'sports physiology' OR 'sports medicine' OR 'rehabilitation science' OR 'rehabilitation medicine' OR Sport* OR athlet* ) OR AB=('sport medicine' OR 'sport physiology' OR 'sports physiology' OR 'sports medicine' OR 'rehabilitation sci-ence' OR 'rehabilitation medicine' OR 'athlete injury' OR 'athlete trauma' OR 'athletic injuries' OR 'athletic injury' OR 'athletic trauma' OR 'injury, athletic' OR 'sport accident' OR 'sport related injury' OR 'sport trauma' OR 'sportrelated injury' OR 'sports injury' OR 'sports related injury' OR 'sports trauma' OR 'sportsrelated injury' OR 'sport injury' OR 're-sumption of sport' OR 'resumption of sporting activities' OR 'return to play (sport)' OR 'return to playing sport' OR 'return to sporting activities' OR 'return to sport' OR Sport* OR athlet*) OR AK=('sport medicine' OR 'sport physiology' OR 'sports physiology' OR 'sports medicine' OR 'rehabilitation sci-ence' OR 'rehabilitation medicine' OR 'athlete injury' OR 'athlete trauma' OR 'athletic injuries' OR 'athletic injury' OR 'athletic trauma' OR 'injury, athletic' OR 'sport accident' OR 'sport related injury' OR 'sport trauma' OR 'sportrelated injury' OR 'sports injury' OR 'sports related injury' OR 'sports trauma' OR 'sportsrelated injury' OR 'sport injury' OR 're-sumption of sport' OR 'resumption of sporting activities' OR 'return to play (sport)' OR 'return to playing sport' OR 'return to sporting activities' OR 'return to sport' OR Sport* OR athlet*) | 304576 |
| 2 | TI=('arthrodynia' OR 'joint ache' OR 'joint pain' OR 'arthralgia' OR myalgia OR muscle-pain* OR muscle-soreness* OR Joint-injur* OR Ligament-injur* OR tendon-injur* OR shin-splint* OR sprain* OR muscle-strain* OR muscle-injur* OR muscular-pain* OR muscular-sore* OR muscular-injur* OR strained-muscle* OR injured-muscle* OR sore-muscle* OR (Pain* NEAR/3 (relie* OR manag* OR control* OR mitigat* OR reduc*)) OR analgesi*) OR AB=('arthrodynia' OR 'joint ache' OR 'joint pain' OR 'arthralgia' OR myalgia OR muscle-pain* OR muscle-soreness* OR Joint-injur* OR Ligament-injur* OR tendon-injur* OR shin-splint* OR sprain* OR muscle-strain* OR muscle-injur* OR muscular-pain* OR muscular-sore* OR muscular-injur* OR strained-muscle* OR injured-muscle* OR sore-muscle*OR (Pain* NEAR/3 (relie* OR manag* OR control* OR mitigat* OR reduc*)) OR analgesi* ) OR AK=('arthrodynia' OR 'joint ache' OR 'joint pain' OR 'arthralgia' OR myalgia OR muscle-pain* OR muscle-soreness* OR Joint-injur* OR Ligament-injur* OR tendon-injur* OR shin-splint* OR sprain* OR muscle-strain* OR muscle-injur* OR muscular-pain* OR muscular-sore* OR muscular-injur* OR strained-muscle* OR injured-muscle* OR sore-muscle* OR (Pain* NEAR/3 (relie* OR manag* OR control* OR mitigat* OR reduc*)) OR analgesi*) | 292682 |
| 3 | TI=('bursitis, radiohumeral' OR 'elbow, tennis' OR 'epicondylatgia humeri' OR 'epicondylitis humeri' OR 'epicondylitis lateralis' OR 'epicondylitis, external humeral' OR 'epicondylitis, radiohumeral' OR 'epycondylitis humeri' OR 'external humeral epicondylitis' OR 'humeral epicondylitis' OR 'humerus epicondylitis' OR 'lateral elbow tendinopathy' OR 'lateral elbow tendinosis' OR 'lateral elbow tendonopathy' OR 'lateral epicondylitis' OR 'lateral humeral epicondylitis' OR 'radiohumeral bursitis' OR 'radiohumeral epicondylitis' OR 'tennis arm' OR 'tennis elbow' OR 'epicondylitis medialis' OR 'golfer arm' OR 'golfer elbow' OR 'medial elbow tendinopathy' OR 'medial elbow tendinosis' OR 'medial humeral epicondylitis' OR 'medial epicondylitis' OR 'anterior cruciate ligament injuries' OR 'anterior cruciate ligament injury' OR 'anterior cruciate knee ligament rupture' OR 'rupture, anterior crucial ligament' OR 'anterior cruciate ligament rupture' OR 'achilles tendinopathy' OR 'achilles tendinosis' OR 'achilles tendonopathy' OR 'achilles tendinitis' OR 'patella femoral pain syndrome' OR 'patellar femoral pain syndrome' OR 'patello-femoral pain syndrome' OR 'patello-femoral stress syndrome' OR 'patello-femoral syndrome' OR 'patellofemoral stress syndrome' OR 'patellofemoral syndrome' OR 'patellofemoral pain syndrome' OR 'bursitis' OR 'calcaneal bursitis' OR 'capped hock' OR 'heel bursitis' OR 'retrocalcaneal bursitis' OR 'infection in the bursa' OR 'infectious bursitis' OR 'infective bursitis' OR 'septic bursitis' OR 'shoulder bursitis' OR 'shoulder`s bursitis' OR 'subacromial-subdeltoid bursitis' OR 'subacromial/subdeltoid bursitis' OR 'subdeltoid bursitis' OR 'subacromial bursitis' OR 'rotator cuff impingement' OR 'rotator cuff syndrome' OR 'rotator cuff tendinitis' OR 'rotator cuff tendonitis' OR 'shoulder cuff syndrome' OR 'shoulder impingement' OR 'subacromial impingement' OR 'subacromial impingement syndrome' OR 'syndrome, shoulder impingement' OR 'shoulder impingement syndrome' OR 'cuff injury' OR 'cuff lesion' OR 'rotator cuff disease' OR 'rotator cuff disorder' OR 'rotator cuff disorders' OR 'rotator cuff injuries' OR 'rotator cuff lesion' OR 'rotator cuff lesions' OR 'rotator cuff pathology' OR 'rotator cuff tendinopathies' OR 'rotator cuff tendinopathy' OR 'shoulder rotator cuff degeneration' OR 'shoulder rotator cuff lesion' OR 'rotator cuff injury' OR 'quadrangular space syndrome' OR 'quadrilateral space syndrome' OR 'rotator cuff ruptures' OR 'rotator cuff tear' OR 'rotator cuff tears' OR 'rotatory cuff rupture' OR 'shoulder rotator cuff rupture' OR 'shoulder rotator cuff tear' OR 'rotator cuff rupture' OR 'apatite-associated destructive arthritis' OR 'cuff tear arthropathy' OR 'Milwaukee shoulder' OR 'Milwaukee shoulder syndrome' OR 'rotator cuff tear arthropathy' OR (('collateral ligament, ulnar' OR 'ligamentum collaterale ulnare' OR 'ulna collateral ligament' OR 'ulnar collateral ligament') AND ('ligament injuries' OR 'ligament trauma' OR 'ligament injury' OR 'ligament rupture')) OR 'fracture, meniscus' OR 'knee meniscal rupture' OR 'knee meniscus injury' OR 'knee meniscus lesion' OR 'knee meniscus trauma' OR 'lesion, meniscus' OR 'meniscal fracture' OR 'meniscal lesion' OR 'meniscal lesions' OR 'meniscal rupture, knee' OR 'meniscopathy' OR 'meniscus damage' OR 'meniscus fracture' OR 'meniscus injury' OR 'meniscus lesion' OR 'meniscus rupture, knee' OR 'meniscus tear' OR 'meniscus trauma' OR 'rupture, meniscus' OR 'Tibial Meniscus Injuries' OR 'torn meniscus' OR 'trauma, meniscus' OR 'knee meniscus rupture' OR 'ankle joint sprain' OR 'ankle ligament sprain' OR 'sprain, ankle' OR 'sprained ankle' OR 'ankle sprain' OR (('ankle ligament, lateral' OR 'lateral ligament, ankle' OR 'ligament, ankle, lateral' OR 'ankle lateral ligament') AND ('ligament injuries' OR 'ligament trauma' OR 'ligament injury' OR 'ligament rupture')) OR (Hip-flexor* AND (strain* OR trauma* OR injur*)) OR Groin-pull* OR pulled-groin* OR groin-injur* OR groin-strain* OR hamstring-pull* OR pulled-hamstring* OR hamstring-injur* OR hamstring-strain*) OR AB=('bursitis, radiohumeral' OR 'elbow, tennis' OR 'epicondylatgia humeri' OR 'epicondylitis humeri' OR 'epicondylitis lateralis' OR 'epicondylitis, external humeral' OR 'epicondylitis, radiohumeral' OR 'epycondylitis humeri' OR 'external humeral epicondylitis' OR 'humeral epicondylitis' OR 'humerus epicondylitis' OR 'lateral elbow tendinopathy' OR 'lateral elbow tendinosis' OR 'lateral elbow tendonopathy' OR 'lateral epicondylitis' OR 'lateral humeral epicondylitis' OR 'radiohumeral bursitis' OR 'radiohumeral epicondylitis' OR 'tennis arm' OR 'tennis elbow' OR 'epicondylitis medialis' OR 'golfer arm' OR 'golfer elbow' OR 'medial elbow tendinopathy' OR 'medial elbow tendinosis' OR 'medial humeral epicondylitis' OR 'medial epicondylitis' OR 'anterior cruciate ligament injuries' OR 'anterior cruciate ligament injury' OR 'anterior cruciate knee ligament rupture' OR 'rupture, anterior crucial ligament' OR 'anterior cruciate ligament rupture' OR 'achilles tendinopathy' OR 'achilles tendinosis' OR 'achilles tendonopathy' OR 'achilles tendinitis' OR 'patella femoral pain syndrome' OR 'patellar femoral pain syndrome' OR 'patello-femoral pain syndrome' OR 'patello-femoral stress syndrome' OR 'patello-femoral syndrome' OR 'patellofemoral stress syndrome' OR 'patellofemoral syndrome' OR 'patellofemoral pain syndrome' OR 'bursitis' OR 'calcaneal bursitis' OR 'capped hock' OR 'heel bursitis' OR 'retrocalcaneal bursitis' OR 'infection in the bursa' OR 'infectious bursitis' OR 'infective bursitis' OR 'septic bursitis' OR 'shoulder bursitis' OR 'shoulder`s bursitis' OR 'subacromial-subdeltoid bursitis' OR 'subacromial/subdeltoid bursitis' OR 'subdeltoid bursitis' OR 'subacromial bursitis' OR 'rotator cuff impingement' OR 'rotator cuff syndrome' OR 'rotator cuff tendinitis' OR 'rotator cuff tendonitis' OR 'shoulder cuff syndrome' OR 'shoulder impingement' OR 'subacromial impingement' OR 'subacromial impingement syndrome' OR 'syndrome, shoulder impingement' OR 'shoulder impingement syndrome' OR 'cuff injury' OR 'cuff lesion' OR 'rotator cuff disease' OR 'rotator cuff disorder' OR 'rotator cuff disorders' OR 'rotator cuff injuries' OR 'rotator cuff lesion' OR 'rotator cuff lesions' OR 'rotator cuff pathology' OR 'rotator cuff tendinopathies' OR 'rotator cuff tendinopathy' OR 'shoulder rotator cuff degeneration' OR 'shoulder rotator cuff lesion' OR 'rotator cuff injury' OR 'quadrangular space syndrome' OR 'quadrilateral space syndrome' OR 'rotator cuff ruptures' OR 'rotator cuff tear' OR 'rotator cuff tears' OR 'rotatory cuff rupture' OR 'shoulder rotator cuff rupture' OR 'shoulder rotator cuff tear' OR 'rotator cuff rupture' OR 'apatite-associated destructive arthritis' OR 'cuff tear arthropathy' OR 'Milwaukee shoulder' OR 'Milwaukee shoulder syndrome' OR 'rotator cuff tear arthropathy' OR (('collateral ligament, ulnar' OR 'ligamentum collaterale ulnare' OR 'ulna collateral ligament' OR 'ulnar collateral ligament') AND ('ligament injuries' OR 'ligament trauma' OR 'ligament injury' OR 'ligament rupture')) OR 'fracture, meniscus' OR 'knee meniscal rupture' OR 'knee meniscus injury' OR 'knee meniscus lesion' OR 'knee meniscus trauma' OR 'lesion, meniscus' OR 'meniscal fracture' OR 'meniscal lesion' OR 'meniscal lesions' OR 'meniscal rupture, knee' OR 'meniscopathy' OR 'meniscus damage' OR 'meniscus fracture' OR 'meniscus injury' OR 'meniscus lesion' OR 'meniscus rupture, knee' OR 'meniscus tear' OR 'meniscus trauma' OR 'rupture, meniscus' OR 'Tibial Meniscus Injuries' OR 'torn meniscus' OR 'trauma, meniscus' OR 'knee meniscus rupture' OR 'ankle joint sprain' OR 'ankle ligament sprain' OR 'sprain, ankle' OR 'sprained ankle' OR 'ankle sprain' OR (('ankle ligament, lateral' OR 'lateral ligament, ankle' OR 'ligament, ankle, lateral' OR 'ankle lateral ligament') AND ('ligament injuries' OR 'ligament trauma' OR 'ligament injury' OR 'ligament rupture')) OR (Hip-flexor* AND (strain* OR trauma* OR injur*)) OR Groin-pull* OR pulled-groin* OR groin-injur* OR groin-strain* OR hamstring-pull* OR pulled-hamstring* OR hamstring-injur* OR hamstring-strain*) OR AK=('bursitis, radiohumeral' OR 'elbow, tennis' OR 'epicondylatgia humeri' OR 'epicondylitis humeri' OR 'epicondylitis lateralis' OR 'epicondylitis, external humeral' OR 'epicondylitis, radiohumeral' OR 'epycondylitis humeri' OR 'external humeral epicondylitis' OR 'humeral epicondylitis' OR 'humerus epicondylitis' OR 'lateral elbow tendinopathy' OR 'lateral elbow tendinosis' OR 'lateral elbow tendonopathy' OR 'lateral epicondylitis' OR 'lateral humeral epicondylitis' OR 'radiohumeral bursitis' OR 'radiohumeral epicondylitis' OR 'tennis arm' OR 'tennis elbow' OR 'epicondylitis medialis' OR 'golfer arm' OR 'golfer elbow' OR 'medial elbow tendinopathy' OR 'medial elbow tendinosis' OR 'medial humeral epicondylitis' OR 'medial epicondylitis' OR 'anterior cruciate ligament injuries' OR 'anterior cruciate ligament injury' OR 'anterior cruciate knee ligament rupture' OR 'rupture, anterior crucial ligament' OR 'anterior cruciate ligament rupture' OR 'achilles tendinopathy' OR 'achilles tendinosis' OR 'achilles tendonopathy' OR 'achilles tendinitis' OR 'patella femoral pain syndrome' OR 'patellar femoral pain syndrome' OR 'patello-femoral pain syndrome' OR 'patello-femoral stress syndrome' OR 'patello-femoral syndrome' OR 'patellofemoral stress syndrome' OR 'patellofemoral syndrome' OR 'patellofemoral pain syndrome' OR 'bursitis' OR 'calcaneal bursitis' OR 'capped hock' OR 'heel bursitis' OR 'retrocalcaneal bursitis' OR 'infection in the bursa' OR 'infectious bursitis' OR 'infective bursitis' OR 'septic bursitis' OR 'shoulder bursitis' OR 'shoulder`s bursitis' OR 'subacromial-subdeltoid bursitis' OR 'subacromial/subdeltoid bursitis' OR 'subdeltoid bursitis' OR 'subacromial bursitis' OR 'rotator cuff impingement' OR 'rotator cuff syndrome' OR 'rotator cuff tendinitis' OR 'rotator cuff tendonitis' OR 'shoulder cuff syndrome' OR 'shoulder impingement' OR 'subacromial impingement' OR 'subacromial impingement syndrome' OR 'syndrome, shoulder impingement' OR 'shoulder impingement syndrome' OR 'cuff injury' OR 'cuff lesion' OR 'rotator cuff disease' OR 'rotator cuff disorder' OR 'rotator cuff disorders' OR 'rotator cuff injuries' OR 'rotator cuff lesion' OR 'rotator cuff lesions' OR 'rotator cuff pathology' OR 'rotator cuff tendinopathies' OR 'rotator cuff tendinopathy' OR 'shoulder rotator cuff degeneration' OR 'shoulder rotator cuff lesion' OR 'rotator cuff injury' OR 'quadrangular space syndrome' OR 'quadrilateral space syndrome' OR 'rotator cuff ruptures' OR 'rotator cuff tear' OR 'rotator cuff tears' OR 'rotatory cuff rupture' OR 'shoulder rotator cuff rupture' OR 'shoulder rotator cuff tear' OR 'rotator cuff rupture' OR 'apatite-associated destructive arthritis' OR 'cuff tear arthropathy' OR 'Milwaukee shoulder' OR 'Milwaukee shoulder syndrome' OR 'rotator cuff tear arthropathy' OR (('collateral ligament, ulnar' OR 'ligamentum collaterale ulnare' OR 'ulna collateral ligament' OR 'ulnar collateral ligament') AND ('ligament injuries' OR 'ligament trauma' OR 'ligament injury' OR 'ligament rupture')) OR 'fracture, meniscus' OR 'knee meniscal rupture' OR 'knee meniscus injury' OR 'knee meniscus lesion' OR 'knee meniscus trauma' OR 'lesion, meniscus' OR 'meniscal fracture' OR 'meniscal lesion' OR 'meniscal lesions' OR 'meniscal rupture, knee' OR 'meniscopathy' OR 'meniscus damage' OR 'meniscus fracture' OR 'meniscus injury' OR 'meniscus lesion' OR 'meniscus rupture, knee' OR 'meniscus tear' OR 'meniscus trauma' OR 'rupture, meniscus' OR 'Tibial Meniscus Injuries' OR 'torn meniscus' OR 'trauma, meniscus' OR 'knee meniscus rupture' OR 'ankle joint sprain' OR 'ankle ligament sprain' OR 'sprain, ankle' OR 'sprained ankle' OR 'ankle sprain' OR (('ankle ligament, lateral' OR 'lateral ligament, ankle' OR 'ligament, ankle, lateral' OR 'ankle lateral ligament') AND ('ligament injuries' OR 'ligament trauma' OR 'ligament injury' OR 'ligament rupture')) OR (Hip-flexor* AND (strain* OR trauma* OR injur*)) OR Groin-pull* OR pulled-groin* OR groin-injur* OR groin-strain* OR hamstring-pull* OR pulled-hamstring* OR hamstring-injur* OR hamstring-strain*) | 55631 |
| 4 | TI=('lidocaine' OR '2 (diethylamino) n (2, 6 dimethylbenzene) acetamide' OR '2 (diethylamino) n (2, 6 dimethylphenyl) acetamide' OR '2 diethylamino 2`, 6` acetoxylidide' OR 'acoin' OR 'actilogic' OR 'aeroderm' OR 'after burn spray' OR 'akten' OR 'algrx 3268' OR 'algrx3268' OR 'alpha diethylamino 2, 6 dimethylacetanilide' OR 'alphacaine' OR 'anestacaine' OR 'anestacon' OR 'anestacone' OR 'aritmal' OR 'asensil' OR 'basicaina' OR 'betacaine' OR 'bobodent' OR 'bravera control' OR 'calmotop' OR 'cidancaina' OR 'col 1077' OR 'col1077' OR 'corus 1030' OR 'corus1030' OR 'cuivasil spray' OR 'dalcaine' OR 'dentinox' OR 'dentipatch' OR 'dequaspray' OR 'dermovagisil' OR 'diethylamino 2, 6 dimethylacetanilide hydrochloride' OR 'diethylaminodimethylacetanilid hydrochloride' OR 'diethylaminodimethylacetanilide hydrochloride' OR 'dolicaine' OR 'dolocopin' OR 'dube spray' OR 'duncaine' OR 'dynexan' OR 'dynexangival' OR 'ecocain' OR 'ela-max' OR 'esliva' OR 'esracain' OR 'esracaine' OR 'farmacaina' OR 'gesicain' OR 'glydo' OR 'gravocain' OR 'instillido' OR 'isicaine' OR 'jetocaine' OR 'jetokain' OR 'l-caine' OR 'lambdalina' OR 'lecasin' OR 'leostesin' OR 'licain' OR 'lida mantle' OR 'lidbree' OR 'lidiaq' OR 'lidiq' OR 'lido-tec' OR 'lidocain' OR 'lidocard' OR 'lidocaton' OR 'lidocor' OR 'lidocorit' OR 'lidoderm' OR 'lidofast' OR 'lidogalen' OR 'lidonest' OR 'lidonostrum' OR 'lidopain' OR 'lidopen' OR 'lidoposterin' OR 'lidorx' OR 'lidosen' OR 'lidotec' OR 'lidothesin' OR 'lignocaine' OR 'lignostab' OR 'lignox' OR 'lincaine' OR 'linisol' OR 'liquocaine' OR 'liris' OR 'll 30' OR 'll30' OR 'lmx 4' OR 'lmx 5' OR 'lmx4' OR 'lonioban' OR 'luan' OR 'maricaine' OR 'maxilene (lidocaine)' OR 'medocaine' OR 'n, n diethyl n` (2, 6 dimethylphenyl) glycinamide' OR 'nanolipo' OR 'neo lidocaton' OR 'neo novutox' OR 'neolidocaton' OR 'nydriar' OR 'omega diethylamino 2, 6 dimethylacetanilide' OR 'ophtesic' OR 'ortodermina' OR 'otipax' OR 'otocalmine' OR 'penles' OR 'posterisan akut' OR 'radiaguard' OR 'ralvo' OR 'remicaine gel' OR 'roxicaina' OR 'rucaina' OR 'ruciana' OR 'ryligency' OR 'solarcaine' OR 'solcaine' OR 'sp 103' OR 'sp103' OR 'trachilid' OR 'trachisan' OR 'truvinut' OR 'truxacaine' OR 'uad caine' OR 'vagisil' OR 'vasocaine' OR 'versatis' OR 'vessatis' OR 'vobolon' OR 'xidocaine' OR 'xilina' OR 'xiline' OR 'xilocaina viscosa' OR 'xilonest pomada' OR 'xilonibsa' OR 'xilotane gel' OR 'xilyne' OR 'xylanaest' OR 'xylcaine' OR 'xylestesin' OR 'xylocain' OR 'xylocaina' OR 'xylocaine' OR 'xylocard' OR 'xylocitin' OR 'xylocontact' OR 'xyloctin' OR 'xylojell' OR 'xyloneural' OR 'xylonor' OR 'xyloproct n' OR 'xyloton' OR 'xylotox' OR 'xylozan' OR 'xylyne' OR 'zingo' OR 'ztlido' OR 'salicylic acid methyl ester' OR '2 hydroxybenzoic acid methyl ester' OR 'betula oil' OR 'gaultheria oil' OR 'methyl 2 hydroxybenzoate' OR 'methyl salicylate' OR 'methylsalicylate' OR 'oil of wintergreen' OR 'sweet birch oil' OR 'wintergreen oil' OR 'Hydroxyethyl-salicylate' OR '2 hydroxyethyl salicylate' OR 'ethyl glycol salicylate' OR 'ethylene glycol monosalicylate' OR 'ethylene glycol salicylate' OR 'ethylene glycol salicylic ester' OR 'ethyleneglycol monosalicylate' OR 'ethyleneglycol monosalicylic acid' OR 'gl 7' OR 'glycol monosalicylate' OR 'glycolsalicylate' OR 'glysal' OR 'monoglycol salicylate' OR 'phardol mono' OR 'rheumacyl' OR 'salicylic acid 2 hydroxyethyl ester' OR 'salicylic acid glycol ester' OR 'salicylic acid monoglycol ester' OR 'spirosal' OR 'traumasenex' OR 'glycol salicylate' OR Ethyl-salicylate OR Tetrahydrofurfuryl-salicylate OR Diethylamine-salicylate OR Acetylsalicylic-acid OR Salicylic-acid OR 'methyl nicotinate' OR 'methylnicotinate' OR 'midalgan' OR 'nicotinic acid methyl ester' OR 'hexyl nicotinate' OR 'hexylnicotinate' OR 'nicotinic acid hexyl ester' OR 'benzylnicotinate' OR 'nicotinic acid benzyl ester' OR 'pycaril' OR 'revulsin' OR 'rubriment' OR 'benzyl nicotinate' OR 'hh 50' OR 'n (3 methoxy 4 hydroxybenzyl) nonanamide' OR 'n (4 hydroxy 3 methoxybenzyl) nonanamide' OR 'n nonanoylvanillylamine' OR 'n vanillylnonamide' OR 'n vanillylnonanamide' OR 'nonanoic acid vanillylamide' OR 'nonanoylvanillylamine' OR 'nonilylic acid vanillylamide' OR 'nonylic acid vanillylamide' OR 'nonylvanillamide' OR 'vanillyl n nonoylamide' OR 'vanillyl n nonylamide' OR 'vanillylnonamide' OR 'vanillylnonanamide' OR 'nonivamide' OR 'nonivamid' OR '2 butoxyethyl nicotinate' OR 'butoxyethyl nicotinate' OR 'n 51' OR 'nicotinic acid 2 butoxyethyl ester' OR 'nicoboxil' OR 'diclofenac' OR '2 (2, 6 dichloroanilino) phenylacetic acid' OR '2 (2, 6 dichlorophenylamino) phenylacetic acid' OR '2 [ (2, 6 dichlorophenyl) amino] benzeneacetic acid' OR '2 [2 [ (2, 6 dichlorophenyl) amino] phenyl] acetic acid' OR 'abdiflam' OR 'abitren' OR 'acuflam' OR 'akis' OR 'algipatch' OR 'algistick' OR 'algopain eze' OR 'algoplast' OR 'allvoran' OR 'almiral' OR 'almiral gel' OR 'almiral sr' OR 'alonpin' OR 'amz 001' OR 'amz001' OR 'antalcalm' OR 'apo-diclofenac ec' OR 'arcanafenac' OR 'arthrifen' OR 'artren' OR 'artrenac' OR 'artrites' OR 'artrites retard' OR 'assaren' OR 'athrofen' OR 'ba 47210' OR 'ba47210' OR 'batafil' OR 'berafen gel' OR 'berifen' OR 'berifen gel' OR 'betaren' OR 'bolabomin' OR 'calozan' OR 'catanac' OR 'catas' OR 'cencenag' OR 'clo-far' OR 'clofec' OR 'clofen' OR 'clonac' OR 'clonaren' OR 'clonodifen' OR 'cordralan' OR 'curinflam' OR 'ddl plaster' OR 'declophen' OR 'decrol' OR 'deflam-k' OR 'deflamat' OR 'delimon (diclofenac)' OR 'delphinac' OR 'denaclof' OR 'depain' OR 'depain plaster' OR 'dic 075v' OR 'dic075v' OR 'diceus' OR 'dicipan' OR 'diclac' OR 'diclac duo' OR 'diclax' OR 'diclax sr' OR 'diclo' OR 'diclo basan' OR 'diclo divido' OR 'diclo divido long' OR 'diclo puren' OR 'diclo recip' OR 'diclo-basan' OR 'diclobasan' OR 'diclobene' OR 'diclod' OR 'diclodent' OR 'diclodoc' OR 'diclodolor' OR 'diclofen' OR 'diclofen cremogel' OR 'diclofenac rekur' OR 'diclofenac resin' OR 'diclofenac resinate' OR 'diclofenac sodium' OR 'dicloflam' OR 'diclohexal' OR 'dicloin' OR 'diclomax' OR 'diclomol' OR 'diclon' OR 'diclopax' OR 'diclophenac sodium' OR 'diclopuren' OR 'dicloral' OR 'dicloran gel' OR 'diclorecip' OR 'dicloren' OR 'dicloreum' OR 'diclosan sr' OR 'diclosian' OR 'diclotec' OR 'diclowal' OR 'dicsnal' OR 'difen' OR 'difena' OR 'difenac' OR 'difenol gel' OR 'difnal k' OR 'dioxaflex' OR 'dioxaflex retard' OR 'divoltar' OR 'dixol' OR 'dixol forte' OR 'doflastad' OR 'doflex' OR 'dolaren' OR 'dolaut gel' OR 'dolflam-retard' OR 'dolo voltaren' OR 'doloflam' OR 'dolotren' OR 'dolotren gel' OR 'dolotren retard' OR 'doragon' OR 'dosanac' OR 'duravolten' OR 'dycon sr' OR 'dyloject' OR 'ecofenac' OR 'econac' OR 'effekton' OR 'effekton retard' OR 'eflagen' OR 'epifenac' OR 'eslofen' OR 'evadol' OR 'evinopon' OR 'feloran' OR 'fenac' OR 'fenadium' OR 'fenaspec' OR 'flameril' OR 'flexagen' OR 'flogofenac' OR 'flogosin d' OR 'flogozan' OR 'fortfen sr' OR 'freejex' OR 'gavin (diclofenac)' OR 'glimbax (diclofenac)' OR 'gp 45840' OR 'grofenac' OR 'hizemin' OR 'imflac' OR 'inac gel' OR 'indicam' OR 'inflamac' OR 'inflanac' OR 'inforce (drug)' OR 'isv 205' OR 'isv205' OR 'jonac gel' OR 'kadiflam' OR 'kinespir' OR 'klofen l' OR 'klotaren' OR 'kriplex' OR 'lesflam' OR 'leviogel' OR 'lifenac' OR 'lofenac' OR 'lotirac' OR 'lynxorb' OR 'magluphen' OR 'merflam' OR 'modifenac' OR 'monoflam' OR 'motifene' OR 'naboal' OR 'nac gel' OR 'naclof' OR 'nacoflar' OR 'nadifen' OR 'novapirina' OR 'novo-difenac' OR 'novolten' OR 'ofenac' OR 'olfen' OR 'olfen gel' OR 'olfen roll-on' OR 'olfen-75 sr' OR 'optanac' OR 'orthophen' OR 'ortofen forte' OR 'osteoflam' OR 'painstop' OR 'panamor' OR 'pennsaid' OR 'profenac' OR 'prosorb-d' OR 'relaxyl gel' OR 'remethan' OR 'remethan gel' OR 'renvol emulgel' OR 'rewodina' OR 'rheufenac' OR 'rheumafen' OR 'rheumatac' OR 'rheumatac retard' OR 'rhewlin' OR 'rhewlin forte' OR 'rhewlin sr' OR 'rhumalgan' OR 'rolactin' OR 'sailib' OR 'savismin' OR 'sefnac' OR 'slofenac' OR 'sodium diclofenac' OR 'solaraze' OR 'sophenoderm' OR 'soproxen' OR 'spraymik' OR 'sr 318t' OR 'staren' OR 'sting gel' OR 'tabiflex' OR 'tds 943' OR 'tds943' OR 'tigen plaster' OR 'toraren' OR 'traulen' OR 'tsudohmin' OR 'uniclonax' OR 'uniren' OR 'urigon (diclofenac)' OR 'valentac' OR 'vartelon' OR 'vartelon gel' OR 'veral' OR 'voldal' OR 'voldic' OR 'voldic emulgel' OR 'volero' OR 'volfenac' OR 'volna-k' OR 'volsaid' OR 'volsaid retard' OR 'volta' OR 'voltadex emulgel' OR 'voltadvance' OR 'voltalen' OR 'voltalen emulgel' OR 'voltalgan' OR 'voltaren' OR 'voltaren acti-go' OR 'voltaren arthritis pain' OR 'voltaren colirio' OR 'voltaren forte' OR 'voltaren gel' OR 'voltaren k migraine' OR 'voltaren ofta' OR 'voltaren oftalmico' OR 'voltaren ophta' OR 'voltaren ophtha' OR 'voltaren ophthalmic' OR 'voltaren retard' OR 'voltaren spe' OR 'voltaren sr' OR 'voltaren xr' OR 'voltaren-xr' OR 'voltarene' OR 'voltarenspe' OR 'voltarol' OR 'voltarol 50' OR 'voltarol one a day muscle pain relief' OR 'voltarol rapid' OR 'voltarol retard' OR 'voltine' OR 'voltral' OR 'voltrix' OR 'voren emulgel' OR 'votalen' OR 'votalen sr' OR 'voveran' OR 'voveran emulgel' OR 'vurdon' OR 'wergyl' OR 'xenid' OR 'yuren' OR 'zolterol' OR 'zolterol sr' OR 'zorvolex' OR 'dolostrip' OR 'VoltarenPlast' OR NSAIDS OR Paracetamol OR Ibuprofen OR Naproxen OR 'algesalona (etofenamate)' OR 'algesalone' OR 'bay d 1107' OR 'bay d1107' OR 'bayro gel' OR 'bayrogel' OR 'bph 485' OR 'bph485' OR 'etophenamate' OR 'flufenamic acid 2 (2 hydroxyethyl) ethyl ester' OR 'reumagel' OR 'rheumon' OR 'rheumon gel' OR 'traumon' OR 'ty 485' OR 'ty485' OR 'etofenamate' OR '2 [2 (6, 6 dimethylbicyclo (3.1.1) hept 2 en 2 yl) ethoxy] triethylamine' OR 'algesal suractive' OR 'homomyrtenyloxytriethylamine' OR 'nopoxamine' OR 'myrtecaine' OR '1, 2 diphenyl 3, 5 dioxo 4 n butylpyrazolidine' OR '1, 2 diphenyl 3, 5 dioxo 4 normal butylpyrazolidine' OR '3, 5 dioxo 1, 2 diphenyl 4 n butylpyrazolidine' OR '4 butyl 1, 2 diphenyl 3, 5 dioxopyrazolidine' OR '4 butyl 1, 2 diphenyl 3, 5 pyrazolidinedione' OR 'alindor' OR 'alka butazolidin' OR 'alpha phenylbutazone' OR 'antadol' OR 'anuspiramin' OR 'anuspiranim' OR 'apo-phenylbutazone' OR 'artgrizone' OR 'arthril' OR 'arthrizin' OR 'arthrizon' OR 'arthryl (phenylbutazone)' OR 'artrizin' OR 'autadol' OR 'azolid' OR 'baliomel' OR 'basireuma' OR 'bumzolidina' OR 'busone' OR 'butacote' OR 'butadion' OR 'butadiona' OR 'butadione' OR 'butalidon' OR 'butapirazol' OR 'butapyrasol' OR 'butartril' OR 'butasan' OR 'butatab' OR 'butazina' OR 'butazine' OR 'butazolidin' OR 'butazolidin ointment' OR 'butazolidina' OR 'butazolidine' OR 'butazone-100' OR 'butidiona' OR 'butidione' OR 'butrex' OR 'butylpyridin' OR 'buzon' OR 'delta butazolidin' OR 'delta elmedal' OR 'diossidone' OR 'diozal' OR 'diphebuzol' OR 'diphenbutazone' OR 'diphenylbutazone' OR 'ecobutazon' OR 'ecobutazone' OR 'elmedal' OR 'equipalazone' OR 'exrheudon n' OR 'febuzine' OR 'fenbutazona' OR 'fenibutazona' OR 'fenibutazone' OR 'fenibutol' OR 'fenilbutazona' OR 'fenilbutazona gen-far' OR 'fenilbutazona mckesson' OR 'fenilbutina' OR 'fenotone' OR 'fenyl butazon' OR 'fenylbutazone' OR 'g 13871' OR 'g13871' OR 'ia but' OR 'intrabutazone' OR 'intrazone' OR 'ircodin' OR 'irgapan' OR 'mcn r 495' OR 'nadozone' OR 'neuro elmedal' OR 'phebuzine' OR 'phenbutazole' OR 'phenbutazone' OR 'phenobutazone' OR 'phenyl butazone' OR 'phenylarthrite' OR 'phenylbutasone' OR 'phenylbutazon' OR 'phenylbutazone ointment' OR 'phenylbutazone sodium' OR 'phenylmobuzon' OR 'pirarreumol b' OR 'pirrareumol b' OR 'pyrabutol' OR 'pyrazolidin' OR 'pyrazone' OR 'r 3 zon' OR 'reudo' OR 'reudox' OR 'reumuzol' OR 'servizolidin' OR 'shigrodin' OR 'sodium phenylbutazone' OR 'spondyril' OR 'ticinil' OR 'zolandin' OR 'zolaphen' OR 'phenylbutazone' OR '12, 19 difluoro 11 hydroxy 8 (2 hydroxyacetyl) 6, 6, 9, 13 tetramethyl 5, 7 dioxapentacyclo [10.8.0.0 (2, 9) .0 (4, 8) .0 (13, 18)] icosa 14, 17 dien 16 one' OR '4b, 12 difluoro 5 hydroxy 6b (hydroxyacetyl) 4a, 6a, 8, 8 tetramethyl 4a, 4b, 5, 6, 6a, 6b, 9a, 10, 10a, 10b, 11, 12 dodecahydro 2h naphtho [2`, 1`:4, 5] indeno [1, 2 d] [1, 3] dioxol 2 one' OR '4b, 12 difluoro 6b glycoloyl 5 hydroxy 4a, 6a, 8, 8 tetramethyl 4a, 4b, 5, 6, 6a, 6b, 9a, 10, 10a, 10b, 11, 12 dodecahydro 2h naphtho [2`, 1`:4, 5] indeno [1, 2 d] [1, 3] dioxol 2 one' OR '6, 9 difluoro 11, 21 dihydroxy 16, 17 [ (1 methylethylidene) bis (oxy)] pregna 1, 4 diene 3, 20 dione' OR '6alpha, 9alpha difluoro 11beta, 16alpha, 17alpha, 21 tetrahydroxy 1, 4 pregnadiene 3, 20 dione 16, 17 acetonide' OR '6alpha, 9alpha difluoro 16alpha hydroxyprednisolone 16, 17 acetonide' OR 'adermina' OR 'alfabios' OR 'alvadermo fuerte' OR 'aplosyn' OR 'capex' OR 'cervicum' OR 'cinolon' OR 'clofeet' OR 'cortilona' OR 'cremisona' OR 'cynozet' OR 'derma-smooth/fs' OR 'derma-smoothe/fs' OR 'dermalar' OR 'dermoflam' OR 'dermoran' OR 'dermotic' OR 'df 277' OR 'df277' OR 'ekzem (drug)' OR 'esacinone' OR 'flac (fluocinolone acetonide)' OR 'flozet' OR 'fluciderm' OR 'flucinar' OR 'flucinolone acetonide' OR 'flulone' OR 'flunolone-v' OR 'fluocet' OR 'fluocid' OR 'fluocid forte' OR 'fluocinolon acetonid' OR 'fluocinolone 16, 17 acetonide' OR 'fluocinonide acetonide' OR 'fluoderm' OR 'fluolar' OR 'fluonid' OR 'fluonide' OR 'fluotrex' OR 'fluovitef' OR 'fluquinol' OR 'flurosyn' OR 'flusonlen' OR 'fluzon' OR 'fs shampoo' OR 'fusalar' OR 'gelargin' OR 'gelidina' OR 'iluvien' OR 'inoderm' OR 'jellin' OR 'lluvien' OR 'localyn' OR 'localyn glicole' OR 'luci' OR 'medidur' OR 'nasal fluocinolone acetonide' OR 'nasal synalar' OR 'neosynalar' OR 'nsc 92339' OR 'nsc92339' OR 'ot 401' OR 'ot401' OR 'otazem' OR 'otoflamm' OR 'otoken' OR 'psoranide' OR 'radiocin' OR 'retisert' OR 'rs 1401 at' OR 'rs 1401at' OR 'rs1401 at' OR 'rs1401at' OR 'sinaflana' OR 'supralan' OR 'synalar' OR 'synalar 25' OR 'synalar forte' OR 'synalar gamma' OR 'synalar hp' OR 'synalar nasal' OR 'synalar simple' OR 'synalar-hp' OR 'synandone' OR 'synemol' OR 'synotic' OR 'syntopic' OR 'trisyn' OR 'yutiq' OR 'fluocinolone acetonide' OR Piroxicam OR '1 (3 dimethylaminopropylidene) 2, 3:6, 7 dibenzo 4 suberene' OR '3 (5h dibenzo [a, d] cyclohepten 5 ylidene) n, n dimethylpropylamine' OR '5 (3 dimethylaminopropylidene) (5h) dibenzo (a, d) cycloheptene' OR '5 (3 dimethylaminopropylidene) dibenzo (a, e) cycloheptatriene' OR '5 (3 dimethylaminopropylidene) dibenzo [a, e] cycloheptatriene' OR 'amrix' OR 'cyben' OR 'cyclobenzaprine hydrochloride' OR 'flexeril' OR 'flexiban' OR 'mk 130' OR 'mk130' OR 'n, n dimethyl 5h debenzo [a, d] cycloheptene delta5, alpha propylamine' OR 'proheptatriene' OR 'rp 9715' OR 'rp9715' OR 'tensodox' OR 'yurelax' OR 'cyclobenzaprine' OR '10, 11 dihydro 5 (gamma dimethylaminopropylidene) 5h dibenzo [a, d] 1, 4 cycloheptene' OR '10, 11 dihydro n, n dimethyl 5h dibenzo [a, d] cycloheptene delta5, gamma propylamine' OR '3 (10, 11 dihydro 5h dibenzo [a, d] cycloheptene 5 ylidene) n, n dimethylpropylamine' OR '5 (3 dimethylaminopropylidene) 10, 11 dihydro 5h dibenzo [a, d] cycloheptene' OR '5 (3 dimethylaminopropylidene) dibenzo [a, d] [1, 4] cycloheptadiene' OR '5 (gamma dimethylaminopropylidene) 5h dibenzo [a, d] 10, 11 dihydrocycloheptene' OR 'adepress' OR 'adepril' OR 'ambivalon' OR 'amilit' OR 'amineurin' OR 'amineurin retard' OR 'amiplin' OR 'amiprin' OR 'amirol' OR 'amirol 10 fc' OR 'amirol 25 fc' OR 'amitid' OR 'amitril' OR 'amitrip' OR 'amitriptylene' OR 'amitriptylene hydrochloride' OR 'amitriptylin' OR 'amitriptylin-ct' OR 'amitriptyline hydrochloride' OR 'amitriptylinumhydrochloride' OR 'amitryptiline' OR 'amitryptiline hydrochloride' OR 'amitryptilline' OR 'amitryptine' OR 'amitryptyline' OR 'amyline' OR 'amytril' OR 'amytriptiline' OR 'amytriptyline' OR 'amytryptiline' OR 'amyzol' OR 'anapsique' OR 'anp 3548' OR 'anp3548' OR 'antalin' OR 'antitriptyline' OR 'apo-amitriptyline' OR 'astilin' OR 'atx 01 (aliphatic amine)' OR 'atx01 (aliphatic amine)' OR 'damilen' OR 'damilene' OR 'damitriptyline' OR 'damylene' OR 'deprelio' OR 'domical' OR 'elatrol' OR 'elatrolet' OR 'elavil' OR 'enafon' OR 'endep' OR 'enovil' OR 'etafon' OR 'etafron' OR 'euplit' OR 'lantron' OR 'laroxal' OR 'laroxyl' OR 'lentizol' OR 'miketorin' OR 'mk 230' OR 'mk230' OR 'n 750' OR 'n, n dimethyl 3 (2 tricyclo [9.4.0.0 (3, 8)] pentadeca 1 (15), 3, 5, 7, 11, 13 hexaenylidene) propan 1 amine' OR 'n, n dimethyl 3 (dibenzo [a.d] 1, 4 cycloheptadien 5 yliden) propylamine hydrochloride' OR 'n750' OR 'novoprotect' OR 'ormal' OR 'pinsaun' OR 'proheptadien' OR 'qualitriptine' OR 'redomex' OR 'redomex diffucaps' OR 'ro 4 1575' OR 'ro4 1575' OR 'ro41575' OR | 545096 |
|  | 'sarboten retard 75' OR 'sarotard' OR 'saroten' OR 'saroten retard' OR 'sarotena' OR 'sarotex' OR 'sarotex retard' OR 'stelminal' OR 'sylvemid' OR 'syneudon' OR 'syneydon' OR 'teperin' OR 'terepin' OR 'trepiline' OR 'tridep' OR 'tripta' OR 'triptanol' OR 'triptizol' OR 'triptyl' OR 'triptyline' OR 'trynol' OR 'tryptanol' OR 'tryptizol' OR 'trytomer' OR 'uxen' OR 'vanatrip' OR 'amitriptyline' OR '2 (2 chlorophenyl) 2 (methylamino) cyclohexanone' OR '2 (2 chlorophenyl) 2 (methylamino) cyclohexanone hydrochloride' OR '2 (2 chlorophenyl) 2 methylaminocyclohexanone' OR '2 (methylamino) 2 (2 chlorophenyl) cyclohexanone' OR '2 (ortho chlorophenyl) 2 (methylamino) cyclohexanone' OR '2 (ortho chlorophenyl) 2 methylaminocyclohexanone' OR '2 (ortho chlorophenyl) 2 methylaminocyclohexanone hydrochloride' OR '2 methylamino 2 (2 chlorophenyl) cyclohexanone' OR '2 ortho chlorophenyl 2 methylaminocyclohexanone' OR 'anesject' OR 'calipsol' OR 'calypsol' OR 'ci 581' OR 'ci581' OR 'cl 369' OR 'cl369' OR 'cn 52, 372 2' OR 'cn 52372 2' OR 'cn 523722' OR 'cn52, 372 2' OR 'cn52372 2' OR 'cn523722' OR 'ereska' OR 'imalgene' OR 'kalipsol' OR 'katamine' OR 'keta-hameln' OR 'ketaject' OR 'ketalar' OR 'ketalin' OR 'ketamax' OR 'ketamine hcl' OR 'ketamine hydrochloride' OR 'ketaminol vet' OR 'ketanest' OR 'ketased' OR 'ketaset' OR 'ketaved' OR 'ketavet' OR 'ketmin' OR 'ketoject' OR 'ketolar' OR 'narkamon' OR 'narketan' OR 'pmi 100' OR 'pmi 150' OR 'pmi100' OR 'pmi150' OR 'sls 002' OR 'sls002' OR 'soon-soon' OR 'tekam' OR 'tur 002' OR 'tur002' OR 'velonarcon' OR 'vetalar' OR 'ketamine' OR '3 (4 chlorophenyl) 4 aminobutanoic acid' OR '3 (4 chlorophenyl) 4 aminobutyric acid' OR '3 (para chlorophenyl) 4 aminobutanoic acid' OR '3 (para chlorophenyl) 4 aminobutyric acid' OR '4 amino 3 (4 chlorophenyl) butanoic acid' OR '4 amino 3 (4 chlorophenyl) butyric acid' OR 'all 1401' OR 'all1401' OR 'alpha-baclofen' OR 'ba 34 647' OR 'ba 34647' OR 'ba34 647' OR 'ba34647' OR 'backen' OR 'baclan' OR 'baclapone' OR 'baclaxiro' OR 'baclo' OR 'baclocur' OR 'baclofene' OR 'baclon' OR 'baclopar' OR 'baclophen' OR 'baclosal' OR 'bacofen' OR 'bacron' OR 'bafen' OR 'baklofen' OR 'baropan' OR 'beta (4 chlorophenyl) 4 aminobutyric acid' OR 'beta (4 chlorophenyl) gamma aminobutyric acid' OR 'beta (aminomethyl) 4 chlorobenzenepropanoic acid' OR 'beta (aminomethyl) 4 chlorocinnamic acid' OR 'beta (aminomethyl) 4 chlorohydrocinnamic acid' OR 'beta (aminomethyl) para chlorocinnamic acid' OR 'beta (para chlorophenyl) 4 aminobutyric acid' OR 'beta 4 chlorophenyl 4 aminobutanoic acid' OR 'beta 4 chlorophenyl gamma aminobutyric acid' OR 'beta amino methyl chlorohydrocinnamic acid' OR 'beta aminomethyl para chlorohydrocinnamic acid' OR 'beta para chlorophenyl gamma aminobutyric acid' OR 'bigafen' OR 'ciba 34647 ba' OR 'ciba 34647ba' OR 'ciba34647ba' OR 'colmifen' OR 'curofen' OR 'dextro baclofen' OR 'espast' OR 'fleqsuvy' OR 'gabalon' OR 'gablofen' OR 'intralcal' OR 'ipx 056' OR 'ipx056' OR 'kemstro' OR 'lebic' OR 'levo baclofen' OR 'liofen' OR 'lionova' OR 'lioresal' OR 'lioresal intrathecal' OR 'lioresyl' OR 'liotec' OR 'lyflex' OR 'lyvispah' OR 'miorel (baclofen)' OR 'neteka' OR 'onelaxant-r' OR 'ozobax' OR 'ozobax ds' OR 'pacifen' OR 'spacyr' OR 'spinax' OR 'stelax' OR 'vioridon' OR 'baclofen' OR '19583 rp' OR '2 (3 benzoylphenyl) propionic acid' OR '2 (3 benzoylphenyl) propionic acid' OR '2 [3 benzoyl (phenyl)] propionic acid' OR 'actron (ketoprofen)' OR 'alreumat' OR 'alrhemun' OR 'alrheumat' OR 'alrheumin' OR 'alrheumun' OR 'alrhumat' OR 'aneol' OR 'anzema' OR 'apo-keto' OR 'arcental' OR 'begsan' OR 'bi-profenid' OR 'bi-profenid lp' OR 'bi-rofenid' OR 'biprofenid' OR 'birofenid' OR 'capisten' OR 'cetoprofen' OR 'diractin' OR 'dolofar' OR 'drastirel' OR 'efiken' OR 'els m11' OR 'elsm11' OR 'epatec' OR 'extraplus (drug)' OR 'fastum' OR 'fetik' OR 'flexen (ketoprofen)' OR 'floramil' OR 'gabrilen' OR 'gabrilen retard' OR 'gesicpad k' OR 'helenil' OR 'ibifen' OR 'idea 033' OR 'idea033' OR 'impracor' OR 'ipi 110' OR 'ipi110' OR 'iso k (drug)' OR 'jomezal' OR 'kaltrofen' OR 'kebanon' OR 'keduril' OR 'kefen' OR 'kehancer' OR 'kenhancer' OR 'kenofen gel' OR 'keotsan' OR 'keplat' OR 'keprofen' OR 'ketadom' OR 'ketin' OR 'keto film' OR 'ketofarm' OR 'ketofen' OR 'ketoflam' OR 'ketolgin' OR 'ketolgin gel' OR 'ketolgin sr' OR 'ketomex' OR 'ketonal' OR 'ketoprofen sodium' OR 'ketoprofene' OR 'ketores' OR 'ketorin' OR 'ketosolan' OR 'ketospray' OR 'ketotransdel' OR 'ketum' OR 'knavon' OR 'kpl 202' OR 'liotondol' OR 'lisactiv gola' OR 'mohrus' OR 'naxal' OR 'nexcede' OR 'novo-keto-ec' OR 'opokan keto' OR 'oralcare (drug)' OR 'orochet' OR 'orucote' OR 'orudis' OR 'orudis e-100' OR 'orudis ec' OR 'orudis kt' OR 'orudis r' OR 'orudis r-pr' OR 'orudis retard' OR 'orudis sr' OR 'oruvail' OR 'oruvail ec' OR 'oruvail sr' OR 'oscorel' OR 'ovurila' OR 'ovurila e' OR 'oxoprofene' OR 'phardol' OR 'profecom' OR 'profemigr' OR 'profenid' OR 'profenid 50' OR 'profenid lp' OR 'profika' OR 'prontoflex' OR 'protofen' OR 'provail cr' OR 'rematof' OR 'rhetoflam' OR 'rheuna pap' OR 'rofenid' OR 'rofixdol' OR 'rp 19, 583' OR 'rp 19583' OR 'siduro' OR 'sodium ketoprofen' OR 'spondylon' OR 'tdlp 110' OR 'tdlp110' OR 'tiloket' OR 'topofen' OR 'toprec' OR 'treosin' OR 'valket' OR 'yerasel' OR 'ketoprofen' OR '(2, 6 dichlorophenylamine) 2 imidazoline hydrochloride' OR '2 (2, 6 dichloranilino) 2 imidazoline' OR '2 (2, 6 dichloroanilino) 2 imidazoline' OR '2 (2, 6 dichloroanilino) 2 imidazoline hydrochloride' OR '2 (2, 6 dichloroanilino) imidazoline' OR '2 (2, 6 dichlorophenyl 1 amino) 2 imidazoline' OR '2 (2, 6 dichlorophenylamino) 2 imidazoline' OR '2 (2, 6 dichlorophenylamino) 2 imidazoline hydrochloride' OR '2 (2, 6 dichlorophenylamino) imidazoline' OR '2 [ (2, 6 dichlorophenyl) imino] imidazoline' OR '2 [2, 6 dichlorphenylamino] 2 imidazoline hydrochloride' OR '2, 6 dichloro n 2 imidazolidinylidenebenzenamine' OR '2, 6 dichloro n imidazolidin 2 ylideneaniline' OR '2, 6 dichlorophenylamine 2 imidazoline hydrochloride' OR 'adesipress tts 2' OR 'adesipress tts2' OR 'arkamin' OR 'atensina' OR 'calotep' OR 'caprysin' OR 'catapres' OR 'catapres tts' OR 'catapres-tts-1' OR 'catapres-tts-2' OR 'catapres-tts-3' OR 'catapresan' OR 'catapresan 100' OR 'catapresan depot' OR 'catapresan tts' OR 'catapressan' OR 'catapressan perlonguettes' OR 'catapressant' OR 'catasan' OR 'chlofazolin' OR 'chlophazolin' OR 'chlophelin' OR 'clinidine' OR 'clofelin' OR 'clofeline' OR 'clomidine' OR 'clondine' OR 'clonicel' OR 'clonidin' OR 'clonidine chlorhydrate' OR 'clonidine hydrochloride' OR 'clonidine tts-1' OR 'clonidine tts-2' OR 'clonidine tts-3' OR 'clonipresan' OR 'clonistada' OR 'clonistada retard' OR 'clonnirit' OR 'clophelin' OR 'clopheline' OR 'daipres' OR 'dcai' OR 'dichlorophenylaminoimidazoline' OR 'dichlorophenylaminoimidazoline hydrochloride' OR 'dixarit' OR 'duraclon' OR 'haemiton' OR 'hemiton' OR 'huma-clonidine' OR 'hypodine' OR 'isoglaucon' OR 'jenloga' OR 'kapvay' OR 'm 5041t' OR 'm5041t' OR 'melzin' OR 'n (2, 6 dichlorophenyl) 4, 5 dihydro 1h imidazol 2 amine' OR 'n (2, 6 dichlorophenyl) 4, 5 dihydro 2 imidazolamine' OR 'n (2, 6 dichlorophenyl) 4, 5 dihydroimidazol 2 amine' OR 'nexiclon' OR 'nexiclon xr' OR 'normopresan' OR 'normopresin' OR 'onyda' OR 'onyda xr' OR 'paracefan' OR 'st 155' OR 'st155' OR 'sulmidine' OR 'taitecin' OR 'tenso timelets' OR 'clonidine' OR '1 (aminomethyl) cyclohexaneacetic acid' OR '2 [1 (aminomethyl) cyclohexyl] acetic acid' OR 'ci 945' OR 'ci945' OR 'dineurin' OR 'dm 1796' OR 'dm 5689' OR 'dm1796' OR 'dm5689' OR 'gabalept' OR 'gabaliquid geriasan' OR 'gabapen' OR 'gabatin' OR 'gantin' OR 'go 3450' OR 'go3450' OR 'goe 3450' OR 'goe3450' OR 'gralise' OR 'kaptin' OR 'keneil' OR 'neurontin' OR 'neurotonin' OR 'nupentin' OR 'sefelsa' OR 'serada' OR 'gabapentin' OR Corticosteroid* OR Menthol OR Levomenthol OR Peppermint-oil OR Clove-oil OR camphor OR capsaicin OR Cajuput-oil OR Cineole OR eucalyptol OR ben-gay OR bengay OR icy-hot OR icyhot ) OR AB=('lidocaine' OR '2 (diethylamino) n (2, 6 dimethylbenzene) acetamide' OR '2 (diethylamino) n (2, 6 dimethylphenyl) acetamide' OR '2 diethylamino 2`, 6` acetoxylidide' OR 'acoin' OR 'actilogic' OR 'aeroderm' OR 'after burn spray' OR 'akten' OR 'algrx 3268' OR 'algrx3268' OR 'alpha diethylamino 2, 6 dimethylacetanilide' OR 'alphacaine' OR 'anestacaine' OR 'anestacon' OR 'anestacone' OR 'aritmal' OR 'asensil' OR 'basicaina' OR 'betacaine' OR 'bobodent' OR 'bravera control' OR 'calmotop' OR 'cidancaina' OR 'col 1077' OR 'col1077' OR 'corus 1030' OR 'corus1030' OR 'cuivasil spray' OR 'dalcaine' OR 'dentinox' OR 'dentipatch' OR 'dequaspray' OR 'dermovagisil' OR 'diethylamino 2, 6 dimethylacetanilide hydrochloride' OR 'diethylaminodimethylacetanilid hydrochloride' OR 'diethylaminodimethylacetanilide hydrochloride' OR 'dolicaine' OR 'dolocopin' OR 'dube spray' OR 'duncaine' OR 'dynexan' OR 'dynexangival' OR 'ecocain' OR 'ela-max' OR 'esliva' OR 'esracain' OR 'esracaine' OR 'farmacaina' OR 'gesicain' OR 'glydo' OR 'gravocain' OR 'instillido' OR 'isicaine' OR 'jetocaine' OR 'jetokain' OR 'l-caine' OR 'lambdalina' OR 'lecasin' OR 'leostesin' OR 'licain' OR 'lida mantle' OR 'lidbree' OR 'lidiaq' OR 'lidiq' OR 'lido-tec' OR 'lidocain' OR 'lidocard' OR 'lidocaton' OR 'lidocor' OR 'lidocorit' OR 'lidoderm' OR 'lidofast' OR 'lidogalen' OR 'lidonest' OR 'lidonostrum' OR 'lidopain' OR 'lidopen' OR 'lidoposterin' OR 'lidorx' OR 'lidosen' OR 'lidotec' OR 'lidothesin' OR 'lignocaine' OR 'lignostab' OR 'lignox' OR 'lincaine' OR 'linisol' OR 'liquocaine' OR 'liris' OR 'll 30' OR 'll30' OR 'lmx 4' OR 'lmx 5' OR 'lmx4' OR 'lonioban' OR 'luan' OR 'maricaine' OR 'maxilene (lidocaine)' OR 'medocaine' OR 'n, n diethyl n` (2, 6 dimethylphenyl) glycinamide' OR 'nanolipo' OR 'neo lidocaton' OR 'neo novutox' OR 'neolidocaton' OR 'nydriar' OR 'omega diethylamino 2, 6 dimethylacetanilide' OR 'ophtesic' OR 'ortodermina' OR 'otipax' OR 'otocalmine' OR 'penles' OR 'posterisan akut' OR 'radiaguard' OR 'ralvo' OR 'remicaine gel' OR 'roxicaina' OR 'rucaina' OR 'ruciana' OR 'ryligency' OR 'solarcaine' OR 'solcaine' OR 'sp 103' OR 'sp103' OR 'trachilid' OR 'trachisan' OR 'truvinut' OR 'truxacaine' OR 'uad caine' OR 'vagisil' OR 'vasocaine' OR 'versatis' OR 'vessatis' OR 'vobolon' OR 'xidocaine' OR 'xilina' OR 'xiline' OR 'xilocaina viscosa' OR 'xilonest pomada' OR 'xilonibsa' OR 'xilotane gel' OR 'xilyne' OR 'xylanaest' OR 'xylcaine' OR 'xylestesin' OR 'xylocain' OR 'xylocaina' OR 'xylocaine' OR 'xylocard' OR 'xylocitin' OR 'xylocontact' OR 'xyloctin' OR 'xylojell' OR 'xyloneural' OR 'xylonor' OR 'xyloproct n' OR 'xyloton' OR 'xylotox' OR 'xylozan' OR 'xylyne' OR 'zingo' OR 'ztlido' OR 'salicylic acid methyl ester' OR '2 hydroxybenzoic acid methyl ester' OR 'betula oil' OR 'gaultheria oil' OR 'methyl 2 hydroxybenzoate' OR 'methyl salicylate' OR 'methylsalicylate' OR 'oil of wintergreen' OR 'sweet birch oil' OR 'wintergreen oil' OR 'Hydroxyethyl-salicylate' OR '2 hydroxyethyl salicylate' OR 'ethyl glycol salicylate' OR 'ethylene glycol monosalicylate' OR 'ethylene glycol salicylate' OR 'ethylene glycol salicylic ester' OR 'ethyleneglycol monosalicylate' OR 'ethyleneglycol monosalicylic acid' OR 'gl 7' OR 'glycol monosalicylate' OR 'glycolsalicylate' OR 'glysal' OR 'monoglycol salicylate' OR 'phardol mono' OR 'rheumacyl' OR 'salicylic acid 2 hydroxyethyl ester' OR 'salicylic acid glycol ester' OR 'salicylic acid monoglycol ester' OR 'spirosal' OR 'traumasenex' OR 'glycol salicylate' OR Ethyl-salicylate OR Tetrahydrofurfuryl-salicylate OR Diethylamine-salicylate OR Acetylsalicylic-acid OR Salicylic-acid OR 'methyl nicotinate' OR 'methylnicotinate' OR 'midalgan' OR 'nicotinic acid methyl ester' OR 'hexyl nicotinate' OR 'hexylnicotinate' |  |

|  | OR 'nicotinic acid hexyl ester' OR 'benzylnicotinate' OR 'nicotinic acid benzyl ester' OR 'pycaril' OR 'revulsin' OR 'rubriment' OR 'benzyl nicotinate' OR 'hh 50' OR 'n (3 methoxy 4 hydroxybenzyl) nonanamide' OR 'n (4 hydroxy 3 methoxybenzyl) nonanamide' OR 'n nonanoylvanillylamine' OR 'n vanillylnonamide' OR 'n vanillylnonanamide' OR 'nonanoic acid vanillylamide' OR 'nonanoylvanillylamine' OR 'nonilylic acid vanillylamide' OR 'nonylic acid vanillylamide' OR 'nonylvanillamide' OR 'vanillyl n nonoylamide' OR 'vanillyl n nonylamide' OR 'vanillylnonamide' OR 'vanillylnonanamide' OR 'nonivamide' OR 'nonivamid' OR '2 butoxyethyl nicotinate' OR 'butoxyethyl nicotinate' OR 'n 51' OR 'nicotinic acid 2 butoxyethyl ester' OR 'nicoboxil' OR 'diclofenac' OR '2 (2, 6 dichloroanilino) phenylacetic acid' OR '2 (2, 6 dichlorophenylamino) phenylacetic acid' OR '2 [ (2, 6 dichlorophenyl) amino] benzeneacetic acid' OR '2 [2 [ (2, 6 dichlorophenyl) amino] phenyl] acetic acid' OR 'abdiflam' OR 'abitren' OR 'acuflam' OR 'akis' OR 'algipatch' OR 'algistick' OR 'algopain eze' OR 'algoplast' OR 'allvoran' OR 'almiral' OR 'almiral gel' OR 'almiral sr' OR 'alonpin' OR 'amz 001' OR 'amz001' OR 'antalcalm' OR 'apo-diclofenac ec' OR 'arcanafenac' OR 'arthrifen' OR 'artren' OR 'artrenac' OR 'artrites' OR 'artrites retard' OR 'assaren' OR 'athrofen' OR 'ba 47210' OR 'ba47210' OR 'batafil' OR 'berafen gel' OR 'berifen' OR 'berifen gel' OR 'betaren' OR 'bolabomin' OR 'calozan' OR 'catanac' OR 'catas' OR 'cencenag' OR 'clo-far' OR 'clofec' OR 'clofen' OR 'clonac' OR 'clonaren' OR 'clonodifen' OR 'cordralan' OR 'curinflam' OR 'ddl plaster' OR 'declophen' OR 'decrol' OR 'deflam-k' OR 'deflamat' OR 'delimon (diclofenac)' OR 'delphinac' OR 'denaclof' OR 'depain' OR 'depain plaster' OR 'dic 075v' OR 'dic075v' OR 'diceus' OR 'dicipan' OR 'diclac' OR 'diclac duo' OR 'diclax' OR 'diclax sr' OR 'diclo' OR 'diclo basan' OR 'diclo divido' OR 'diclo divido long' OR 'diclo puren' OR 'diclo recip' OR 'diclo-basan' OR 'diclobasan' OR 'diclobene' OR 'diclod' OR 'diclodent' OR 'diclodoc' OR 'diclodolor' OR 'diclofen' OR 'diclofen cremogel' OR 'diclofenac rekur' OR 'diclofenac resin' OR 'diclofenac resinate' OR 'diclofenac sodium' OR 'dicloflam' OR 'diclohexal' OR 'dicloin' OR 'diclomax' OR 'diclomol' OR 'diclon' OR 'diclopax' OR 'diclophenac sodium' OR 'diclopuren' OR 'dicloral' OR 'dicloran gel' OR 'diclorecip' OR 'dicloren' OR 'dicloreum' OR 'diclosan sr' OR 'diclosian' OR 'diclotec' OR 'diclowal' OR 'dicsnal' OR 'difen' OR 'difena' OR 'difenac' OR 'difenol gel' OR 'difnal k' OR 'dioxaflex' OR 'dioxaflex retard' OR 'divoltar' OR 'dixol' OR 'dixol forte' OR 'doflastad' OR 'doflex' OR 'dolaren' OR 'dolaut gel' OR 'dolflam-retard' OR 'dolo voltaren' OR 'doloflam' OR 'dolotren' OR 'dolotren gel' OR 'dolotren retard' OR 'doragon' OR 'dosanac' OR 'duravolten' OR 'dycon sr' OR 'dyloject' OR 'ecofenac' OR 'econac' OR 'effekton' OR 'effekton retard' OR 'eflagen' OR 'epifenac' OR 'eslofen' OR 'evadol' OR 'evinopon' OR 'feloran' OR 'fenac' OR 'fenadium' OR 'fenaspec' OR 'flameril' OR 'flexagen' OR 'flogofenac' OR 'flogosin d' OR 'flogozan' OR 'fortfen sr' OR 'freejex' OR 'gavin (diclofenac)' OR 'glimbax (diclofenac)' OR 'gp 45840' OR 'grofenac' OR 'hizemin' OR 'imflac' OR 'inac gel' OR 'indicam' OR 'inflamac' OR 'inflanac' OR 'inforce (drug)' OR 'isv 205' OR 'isv205' OR 'jonac gel' OR 'kadiflam' OR 'kinespir' OR 'klofen l' OR 'klotaren' OR 'kriplex' OR 'lesflam' OR 'leviogel' OR 'lifenac' OR 'lofenac' OR 'lotirac' OR 'lynxorb' OR 'magluphen' OR 'merflam' OR 'modifenac' OR 'monoflam' OR 'motifene' OR 'naboal' OR 'nac gel' OR 'naclof' OR 'nacoflar' OR 'nadifen' OR 'novapirina' OR 'novo-difenac' OR 'novolten' OR 'ofenac' OR 'olfen' OR 'olfen gel' OR 'olfen roll-on' OR 'olfen-75 sr' OR 'optanac' OR 'orthophen' OR 'ortofen forte' OR 'osteoflam' OR 'painstop' OR 'panamor' OR 'pennsaid' OR 'profenac' OR 'prosorb-d' OR 'relaxyl gel' OR 'remethan' OR 'remethan gel' OR 'renvol emulgel' OR 'rewodina' OR 'rheufenac' OR 'rheumafen' OR 'rheumatac' OR 'rheumatac retard' OR 'rhewlin' OR 'rhewlin forte' OR 'rhewlin sr' OR 'rhumalgan' OR 'rolactin' OR 'sailib' OR 'savismin' OR 'sefnac' OR 'slofenac' OR 'sodium diclofenac' OR 'solaraze' OR 'sophenoderm' OR 'soproxen' OR 'spraymik' OR 'sr 318t' OR 'staren' OR 'sting gel' OR 'tabiflex' OR 'tds 943' OR 'tds943' OR 'tigen plaster' OR 'toraren' |  |
| --- | --- | --- |
| 5 | TI=(topical-analgesi* OR topical-pain-relie* OR transdermal-analgesi* OR transdermal-pain-relie* OR trans-dermal-analgesi* OR trans-dermal-pain-relie* OR transcutaneous-analgesi* OR transcutaneous-pain-relie* OR trans-cutaneous-analgesi* OR trans-cutaneous-pain-relie* OR (Topical* OR transdermal* OR trans-dermal* OR patch* OR ointment* OR cream* OR "gel" OR "gels" OR Liniment*) ) OR AB=(topical-analgesi* OR topical-pain-relie* OR transdermal-analgesi* OR transdermal-pain-relie* OR trans-dermal-analgesi* OR trans-dermal-pain-relie* OR transcutaneous-analgesi* OR transcutaneous-pain-relie* OR trans-cutaneous-analgesi* OR trans-cutaneous-pain-relie* OR (Topical* OR transdermal* OR trans-dermal* OR patch* OR ointment* OR cream* OR "gel" OR "gels" OR Liniment*)) OR AK=(topical-analgesi* OR topical-pain-relie* OR transdermal-analgesi* OR transdermal-pain-relie* OR trans-dermal-analgesi* OR trans-dermal-pain-relie* OR transcutaneous-analgesi* OR transcutaneous-pain-relie* OR trans-cutaneous-analgesi* OR trans-cutaneous-pain-relie* OR (Topical* OR transdermal* OR trans-dermal* OR patch* OR ointment* OR cream* OR "gel" OR "gels" OR Liniment*)) | 1114380 |
| 6 | #1 AND #2 | 12054 |
| 7 | #6 OR #3 | 63071 |
| 8 | #4 AND #5 AND #7 | 138 |
| 9 | #4 AND #5 AND #7 and Review Article or Editorial Material or Note (Exclude – Document Types) | 113 |

CINAHL (EBSCOhost, EBSCO)

| No. | CINAHL Query | Results |
| --- | --- | --- |
| 1 | XB ('sport medicine' OR 'sport physiology' OR 'sports physiology' OR 'sports medicine' OR 'rehabilitation science' OR 'rehabilitation medicine' OR Sport* OR athlet* ) OR AB=('sport medicine' OR 'sport physiology' OR 'sports physiology' OR 'sports medicine' OR 'rehabilitation sci-ence' OR 'rehabilitation medicine' OR 'athlete injury' OR 'athlete trauma' OR 'athletic injuries' OR 'athletic injury' OR 'athletic trauma' OR 'injury, athletic' OR 'sport accident' OR 'sport related injury' OR 'sport trauma' OR 'sportrelated injury' OR 'sports injury' OR 'sports related injury' OR 'sports trauma' OR 'sportsrelated injury' OR 'sport injury' OR 're-sumption of sport' OR 'resumption of sporting activities' OR 'return to play (sport)' OR 'return to playing sport' OR 'return to sporting activities' OR 'return to sport' OR Sport* OR athlet*) OR (MH "Sports Medicine" OR MH "Rehabilitation" OR MH "Athletic Injuries") OR (MH "Sports" OR MH "Aeronautical Sports" OR MH "Amateur Sports" OR MH "Horseback Riding" OR MH "Aquatic Sports+" OR MH "Body Building" OR MH "Bowling" OR MH "Caving" OR MH "College Sports" OR MH "Contact Sports+" OR MH "Cycling" OR MH "Endurance Sports" OR MH "Extreme Sports" OR MH "Fencing" OR MH "Golf" OR MH "Gymnastics" OR MH "Handball" OR MH "Martial Arts" OR MH "Mountaineering" OR MH "Professional Sports" OR MH "Race Walking" OR MH "Racquet Sports+" OR MH "Rock Climbing" OR MH "Running+" OR MH "Skating+" OR MH "Skiing+" OR MH "Sports Re-Entry" OR MH "Target Sports+" OR MH "Team Sports+" OR MH "Track and Field" OR MH "Triathlon" OR MH "Weight Lifting" OR MH "Winter Sports+") OR XB (Sport* OR athlet*) | 173054 |
| 2 | (MH "Pain" OR MH "Groin Pain" OR MH "Knee Pain" OR MH "Metatarsalgia" OR MH "Muscle Pain" OR MH "Musculoskeletal Pain") OR XB ('arthrodynia' OR 'joint ache' OR 'joint pain' OR 'arthralgia' OR myalgia OR muscle-pain* OR muscle-soreness* OR Joint-injur* OR Ligament-injur* OR tendon-injur* OR shin-splint* OR sprain* OR muscle-strain* OR muscle-injur* OR muscular-pain* OR muscular-sore* OR muscular-injur* OR strained-muscle* OR injured-muscle* OR sore-muscle*) OR XB ((Pain* NEAR/3 (relie* OR manag* OR control* OR mitigat* OR reduc*)) OR analgesi*) OR (MH "Pain Management (Iowa NIC)" OR MH "Analgesia" OR MH "Patient-Controlled Analgesia") | 146943 |
| 3 | (XB ('bursitis, radiohumeral' OR 'elbow, tennis' OR 'epicondylatgia humeri' OR 'epicondylitis humeri' OR 'epicondylitis lateralis' OR 'epicondylitis, external humeral' OR 'epicondylitis, radiohumeral' OR 'epycondylitis humeri' OR 'external humeral epicondylitis' OR 'humeral epicondylitis' OR 'humerus epicondylitis' OR 'lateral elbow tendinopathy' OR 'lateral elbow tendinosis' OR 'lateral elbow tendonopathy' OR 'lateral epicondylitis' OR 'lateral humeral epicondylitis' OR 'radiohumeral bursitis' OR 'radiohumeral epicondylitis' OR 'tennis arm' OR 'tennis elbow' OR 'epicondylitis medialis' OR 'golfer arm' OR 'golfer elbow' OR 'medial elbow tendinopathy' OR 'medial elbow tendinosis' OR 'medial humeral epicondylitis' OR 'medial epicondylitis' OR 'anterior cruciate ligament injuries' OR 'anterior cruciate ligament injury' OR 'anterior cruciate knee ligament rupture' OR 'rupture, anterior crucial ligament' OR 'anterior cruciate ligament rupture' OR 'achilles tendinopathy' OR 'achilles tendinosis' OR 'achilles tendonopathy' OR 'achilles tendinitis' OR 'patella femoral pain syndrome' OR 'patellar femoral pain syndrome' OR 'patello-femoral pain syndrome' OR 'patello-femoral stress syndrome' OR 'patello-femoral syndrome' OR 'patellofemoral stress syndrome' OR 'patellofemoral syndrome' OR 'patellofemoral pain syndrome' OR 'bursitis' OR 'calcaneal bursitis' OR 'capped hock' OR 'heel bursitis' OR 'retrocalcaneal bursitis' OR 'infection in the bursa' OR 'infectious bursitis' OR 'infective bursitis' OR 'septic bursitis' OR 'shoulder bursitis' OR 'shoulder`s bursitis' OR 'subacromial-subdeltoid bursitis' OR 'subacromial/subdeltoid bursitis' OR 'subdeltoid bursitis' OR 'subacromial bursitis' OR 'rotator cuff impingement' OR 'rotator cuff syndrome' OR 'rotator cuff tendinitis' OR 'rotator cuff tendonitis' OR 'shoulder cuff syndrome' OR 'shoulder impingement' OR 'subacromial impingement' OR 'subacromial impingement syndrome' OR 'syndrome, shoulder impingement' OR 'shoulder impingement syndrome' OR 'cuff injury' OR 'cuff lesion' OR 'rotator cuff disease' OR 'rotator cuff disorder' OR 'rotator cuff disorders' OR 'rotator cuff injuries' OR 'rotator cuff lesion' OR 'rotator cuff lesions' OR 'rotator cuff pathology' OR 'rotator cuff tendinopathies' OR 'rotator cuff tendinopathy' OR 'shoulder rotator cuff degeneration' OR 'shoulder rotator cuff lesion' OR 'rotator cuff injury' OR 'quadrangular space syndrome' OR 'quadrilateral space syndrome' OR 'rotator cuff ruptures' OR 'rotator cuff tear' OR 'rotator cuff tears' OR 'rotatory cuff rupture' OR 'shoulder rotator cuff rupture' OR 'shoulder rotator cuff tear' OR 'rotator cuff rupture' OR 'apatite-associated destructive arthritis' OR 'cuff tear arthropathy' OR 'Milwaukee shoulder' OR 'Milwaukee shoulder syndrome' OR 'rotator cuff tear arthropathy' OR (('collateral ligament, ulnar' OR 'ligamentum collaterale ulnare' OR 'ulna collateral ligament' OR 'ulnar collateral ligament') AND ('ligament injuries' OR 'ligament trauma' OR 'ligament injury' OR 'ligament rupture')) OR 'fracture, meniscus' OR 'knee meniscal rupture' OR 'knee meniscus injury' OR 'knee meniscus lesion' OR 'knee meniscus trauma' OR 'lesion, meniscus' OR 'meniscal fracture' OR 'meniscal lesion' OR 'meniscal lesions' OR 'meniscal rupture, knee' OR 'meniscopathy' OR 'meniscus damage' OR 'meniscus fracture' OR 'meniscus injury' OR 'meniscus lesion' OR 'meniscus rupture, knee' OR 'meniscus tear' OR 'meniscus trauma' OR 'rupture, meniscus' OR 'Tibial Meniscus Injuries' OR 'torn meniscus' OR 'trauma, meniscus' OR 'knee meniscus rupture' OR 'ankle joint sprain' OR 'ankle ligament sprain' OR 'sprain, ankle' OR 'sprained ankle' OR 'ankle sprain' OR (('ankle ligament, lateral' OR 'lateral ligament, ankle' OR 'ligament, ankle, lateral' OR 'ankle lateral ligament') AND ('ligament injuries' OR 'ligament trauma' OR 'ligament injury' OR 'ligament rupture')) OR (Hip-flexor* AND (strain* OR trauma* OR injur*)) OR Groin-pull* OR pulled-groin* OR groin-injur* OR groin-strain* OR hamstring-pull* OR pulled-hamstring* OR hamstring-injur* OR hamstring-strain*) AND (MH "Bursitis" OR MH "Adhesive Capsulitis" OR MH "Milwaukee Shoulder Syndrome" OR MH "Shoulder Impingement Syndrome" OR MH "Patellofemoral Pain Syndrome" OR MH "Shoulder Pain" OR MH "Knee Pain" OR MH "Rotator Cuff Injuries" OR MH "Anterior Cruciate Ligament Injuries" OR MH "Medial Collateral Ligament Sprain" OR MH "Ulnar Collateral Ligament Injury of the Thumb" OR MH "Meniscal Injuries" OR MH "Ankle Sprain"))) | 8,558 |
| 4 | XB ('lidocaine' OR 'betacaine' OR 'xylocaine' OR 'salicylic acid methyl ester' OR 'betula oil' OR 'gaultheria oil' OR 'methyl salicylate' OR 'methylsalicylate' OR 'oil of wintergreen' OR 'sweet birch oil' OR 'wintergreen oil' OR 'Hydroxyethyl-salicylate' OR 'ethyl glycol salicylate' OR 'ethylene glycol salicylate' OR Ethyl-salicylate OR Tetrahydrofurfuryl-salicylate OR Diethylamine-salicylate OR Acetylsalicylic-acid OR Salicylic-acid OR 'methyl nicotinate' OR 'methylnicotinate' OR 'midalgan' OR 'nicotinic acid methyl ester' OR 'hexyl nicotinate' OR 'hexylnicotinate' OR 'nicotinic acid hexyl ester' OR 'benzylnicotinate' OR 'nicotinic acid benzyl ester' OR 'rubriment' OR 'benzyl nicotinate' OR 'nicoboxil' OR 'diclofenac' OR 'almiral gel' OR 'berafen gel' OR 'berifen gel' OR 'ddl plaster' OR 'depain plaster' OR 'difenol gel' OR 'dolaut gel' OR 'dolotren gel' OR 'inac gel' OR 'jonac gel' OR 'leviogel' OR 'olfen gel' OR 'olfen roll-on' OR 'relaxyl gel' OR OR 'remethan gel' OR 'renvol emulgel' OR 'solaraze' OR 'vartelon gel' OR 'voldic emulgel' OR 'voltaren' OR Ibuprofen OR Naproxen OR 'etofenamate' OR 'phenylbutazone' OR 'fluocinolone acetonide' OR Piroxicam OR 'amitriptyline' OR 'ketoprofen' OR 'clonidine' OR 'gabapentin' OR Corticosteroid* OR Menthol OR Levomenthol OR Peppermint-oil OR Clove-oil OR camphor OR capsaicin OR Cajuput-oil OR Cineole OR eucalyptol OR ben-gay OR bengay OR icy-hot OR icyhot) OR (MH "Antiinflammatory Agents, Non-Steroidal+" OR MH "Acetaminophen" OR MH "Anesthetics, Local+" OR MH "Adrenal Cortex Hormones+" OR MH "Gabapentin" OR MH "Pregabalin" OR MH "Adrenergic alpha-Antagonists+" OR MH "Ketamine" OR MH "Clonidine" OR MH "Diclofenac" OR MH "Lidocaine" OR MH "Neuromuscular Blocking Agents+") | 135,878 |
| 5 | (MH "Administration, Topical" OR MH "Medication Administration: Topical (Iowa NIC)" OR MH "Administration, Transcutaneous" OR MH "Transdermal Patches, Drugs" OR MH "Creams" OR MH "Liniments" OR MH "Ointments") OR XB ((Topical* OR transdermal* OR trans-dermal* OR patch* OR ointment* OR cream* OR "gel" OR "gels" OR Liniment*) OR 'topical analgesi*' OR 'topical pain relie*' OR 'transdermal analgesi*' OR 'transdermal pain relie*' OR 'trans dermal analgesi*' OR 'trans dermal pain relie*' OR 'transcutaneous analgesi*' OR 'transcutaneous pain relie*' OR 'trans cutaneous analgesi*' OR 'trans cutaneous pain relie*') | 60,491 |
| 6 | ((S1 AND S2) OR S3) AND S4 AND S5 | 60 |

SPORTDiscus (EBSCOhost, EBSCO)

| No. | SPORTDiscus Query | Results |
| --- | --- | --- |
| 1 | (DE "SPORTS medicine" OR DE "SPORTS injuries" OR DE "SPORTS physiology" OR DE "SPORTS re-entry" OR DE "SPORTS" OR DE "AMATEUR sports" OR DE "AQUATIC sports" OR DE "BALL games" OR DE "BASEBALL" OR DE "COLLEGE sports" OR DE "COMBAT sports" OR DE "CONTACT sports" OR DE "CROSS-training (Sports)" OR DE "DISC golf" OR DE "ENDURANCE sports" OR DE "EXTREME sports" OR DE "GYMNASTICS" OR DE "HOCKEY" OR DE "INDIVIDUAL sports" OR DE "PARKOUR" OR DE "PROFESSIONAL sports" OR DE "RACKET games" OR DE "RECREATIONAL sports" OR DE "SKATEBOARDING" OR DE "SOFTBALL" OR DE "TEAM sports" OR DE "WINTER sports" OR DE "ATHLETES" OR DE "ATHLETICS" OR DE "RUNNING" OR DE "BAREFOOT running" OR DE "CROSS-country running" OR DE "JOGGING" OR DE "LONG-distance running" OR DE "MIDDLE distance running" OR DE "MINIMALIST running" OR DE "ROAD running" OR DE "SPRINTING" OR DE "TRAIL running" OR DE "WOMEN'S running") OR XB ('sport medicine' OR 'sport physiology' OR 'sports physiology' OR 'sports medicine' OR 'rehabilitation science' OR 'rehabilitation medicine' OR 'athlete injury' OR 'athlete trauma' OR 'athletic injuries' OR 'athletic injury' OR 'athletic trauma' OR 'injury, athletic' OR 'sport accident' OR 'sport related injury' OR 'sport trauma' OR 'sportrelated injury' OR 'sports injury' OR 'sports related injury' OR 'sports trauma' OR 'sportsrelated injury' OR 'sport injury' OR 're-sumption of sport' OR 'resumption of sporting activities' OR 'return to play (sport)' OR 'return to playing sport' OR 'return to sporting activities' OR 'return to sport' OR Sport* OR athlet*) | 670,657 |
| 2 | DE "BURSITIS" OR DE "TENNIS elbow" OR DE "GOLF elbow" OR DE "ACHILLES tendinitis" OR DE "ACHILLES tendon rupture" OR DE "ACHILLES tendon injuries" OR DE "ANTERIOR cruciate ligament injuries" OR DE "ANTERIOR cruciate ligament injuries" OR DE "PATELLOFEMORAL joint injuries" OR DE "PLICA syndrome" OR DE "ROTATOR cuff injuries" OR DE "COLLATERAL ligament injuries" OR DE "MENISCUS injuries" OR DE "SPRAINS" OR DE "AVULSION fractures" OR DE "HAMSTRING muscle injuries" OR DE "GROIN injuries" OR XB ('bursitis, radiohumeral' OR 'elbow, tennis' OR 'epicondylatgia humeri' OR 'epicondylitis humeri' OR 'epicondylitis lateralis' OR 'epicondylitis, external humeral' OR 'epicondylitis, radiohumeral' OR 'epycondylitis humeri' OR 'external humeral epicondylitis' OR 'humeral epicondylitis' OR 'humerus epicondylitis' OR 'lateral elbow tendinopathy' OR 'lateral elbow tendinosis' OR 'lateral elbow tendonopathy' OR 'lateral epicondylitis' OR 'lateral humeral epicondylitis' OR 'radiohumeral bursitis' OR 'radiohumeral epicondylitis' OR 'tennis arm' OR 'tennis elbow' OR 'epicondylitis medialis' OR 'golfer arm' OR 'golfer elbow' OR 'medial elbow tendinopathy' OR 'medial elbow tendinosis' OR 'medial humeral epicondylitis' OR 'medial epicondylitis' OR 'anterior cruciate ligament injuries' OR 'anterior cruciate ligament injury' OR 'anterior cruciate knee ligament rupture' OR 'rupture, anterior crucial ligament' OR 'anterior cruciate ligament rupture' OR 'achilles tendinopathy' OR 'achilles tendinosis' OR 'achilles tendonopathy' OR 'achilles tendinitis' OR 'patella femoral pain syndrome' OR 'patellar femoral pain syndrome' OR 'patello-femoral pain syndrome' OR 'patello-femoral stress syndrome' OR 'patello-femoral syndrome' OR 'patellofemoral stress syndrome' OR 'patellofemoral syndrome' OR 'patellofemoral pain syndrome' OR 'bursitis' OR 'calcaneal bursitis' OR 'capped hock' OR 'heel bursitis' OR 'retrocalcaneal bursitis' OR 'infection in the bursa' OR 'infectious bursitis' OR 'infective bursitis' OR 'septic bursitis' OR 'shoulder bursitis' OR 'shoulder`s bursitis' OR 'subacromial-subdeltoid bursitis' OR 'subacromial/subdeltoid bursitis' OR 'subdeltoid bursitis' OR 'subacromial bursitis' OR 'rotator cuff impingement' OR 'rotator cuff syndrome' OR 'rotator cuff tendinitis' OR 'rotator cuff tendonitis' OR 'shoulder cuff syndrome' OR 'shoulder impingement' OR 'subacromial impingement' OR 'subacromial impingement syndrome' OR 'syndrome, shoulder impingement' OR 'shoulder impingement syndrome' OR 'cuff injury' OR 'cuff lesion' OR 'rotator cuff disease' OR 'rotator cuff disorder' OR 'rotator cuff disorders' OR 'rotator cuff injuries' OR 'rotator cuff lesion' OR 'rotator cuff lesions' OR 'rotator cuff pathology' OR 'rotator cuff tendinopathies' OR 'rotator cuff tendinopathy' OR 'shoulder rotator cuff degeneration' OR 'shoulder rotator cuff lesion' OR 'rotator cuff injury' OR 'quadrangular space syndrome' OR 'quadrilateral space syndrome' OR 'rotator cuff ruptures' OR 'rotator cuff tear' OR 'rotator cuff tears' OR 'rotatory cuff rupture' OR 'shoulder rotator cuff rupture' OR 'shoulder rotator cuff tear' OR 'rotator cuff rupture' OR 'apatite-associated destructive arthritis' OR 'cuff tear arthropathy' OR 'Milwaukee shoulder' OR 'Milwaukee shoulder syndrome' OR 'rotator cuff tear arthropathy' OR (('collateral ligament, ulnar' OR 'ligamentum collaterale ulnare' OR 'ulna collateral ligament' OR 'ulnar collateral ligament') AND ('ligament injuries' OR 'ligament trauma' OR 'ligament injury' OR 'ligament rupture')) OR 'fracture, meniscus' OR 'knee meniscal rupture' OR 'knee meniscus injury' OR 'knee meniscus lesion' OR 'knee meniscus trauma' OR 'lesion, meniscus' OR 'meniscal fracture' OR 'meniscal lesion' OR 'meniscal lesions' OR 'meniscal rupture, knee' OR 'meniscopathy' OR 'meniscus damage' OR 'meniscus fracture' OR 'meniscus injury' OR 'meniscus lesion' OR 'meniscus rupture, knee' OR 'meniscus tear' OR 'meniscus trauma' OR 'rupture, meniscus' OR 'Tibial Meniscus Injuries' OR 'torn meniscus' OR 'trauma, meniscus' OR 'knee meniscus rupture' OR 'ankle joint sprain' OR 'ankle ligament sprain' OR 'sprain, ankle' OR 'sprained ankle' OR 'ankle sprain' OR (('ankle ligament, lateral' OR 'lateral ligament, ankle' OR 'ligament, ankle, lateral' OR 'ankle lateral ligament') AND ('ligament injuries' OR 'ligament trauma' OR 'ligament injury' OR 'ligament rupture')) OR (Hip-flexor* AND (strain* OR trauma* OR injur*)) OR Groin-pull* OR pulled-groin* OR groin-injur* OR groin-strain* OR hamstring-pull* OR pulled-hamstring* OR hamstring-injur* OR hamstring-strain*) | 23,928 |
| 3 | (DE "ANALGESIA" OR DE "PAIN" OR DE "ELBOW pain" OR DE "FOOT pain" OR DE "GROIN pain" OR DE "KNEE pain" OR DE "LEG pain" OR DE "MYALGIA" OR DE "PAIN management" OR DE "SHOULDER pain") OR XB ('arthrodynia' OR 'joint ache' OR 'joint pain' OR 'arthralgia' OR myalgia OR muscle-pain* OR muscle-soreness* OR Joint-injur* OR Ligament-injur* OR tendon-injur* OR shin-splint* OR sprain* OR muscle-strain* OR muscle-injur* OR muscular-pain* OR muscular-sore* OR muscular-injur* OR strained-muscle* OR injured-muscle* OR sore-muscle* OR (Pain* NEAR/3 (relie* OR manag* OR control* OR mitigat* OR reduc*)) OR analgesi*) | 41,155 |
| 4 | XB ('lidocaine' OR 'betacaine' OR 'xylocaine' OR 'salicylic acid methyl ester' OR 'betula oil' OR 'gaultheria oil' OR 'methyl salicylate' OR 'methylsalicylate' OR 'oil of wintergreen' OR 'sweet birch oil' OR 'wintergreen oil' OR 'Hydroxyethyl-salicylate' OR 'ethyl glycol salicylate' OR 'ethylene glycol salicylate' OR Ethyl-salicylate OR Tetrahydrofurfuryl-salicylate OR Diethylamine-salicylate OR Acetylsalicylic-acid OR Salicylic-acid OR 'methyl nicotinate' OR 'methylnicotinate' OR 'midalgan' OR 'nicotinic acid methyl ester' OR 'hexyl nicotinate' OR 'hexylnicotinate' OR 'nicotinic acid hexyl ester' OR 'benzylnicotinate' OR 'nicotinic acid benzyl ester' OR 'rubriment' OR 'benzyl nicotinate' OR 'nicoboxil' OR 'diclofenac' OR 'almiral gel' OR 'berafen gel' OR 'berifen gel' OR 'ddl plaster' OR 'depain plaster' OR 'difenol gel' OR 'dolaut gel' OR 'dolotren gel' OR 'inac gel' OR 'jonac gel' OR 'leviogel' OR 'olfen gel' OR 'olfen roll-on' OR 'relaxyl gel' OR OR 'remethan gel' OR 'renvol emulgel' OR 'solaraze' OR 'vartelon gel' OR 'voldic emulgel' OR 'voltaren' OR Ibuprofen OR Naproxen OR 'etofenamate' OR 'phenylbutazone' OR 'fluocinolone acetonide' OR Piroxicam OR 'amitriptyline' OR 'ketoprofen' OR 'clonidine' OR 'gabapentin' OR Corticosteroid* OR Menthol OR Levomenthol OR Peppermint-oil OR Clove-oil OR camphor OR capsaicin OR Cajuput-oil OR Cineole OR eucalyptol OR ben-gay OR bengay OR icy-hot OR icyhot) OR (DE "ANALGESICS" OR DE "ACETAMINOPHEN" OR DE "CLONIDINE" OR DE "DEXMEDETOMIDINE" OR DE "IBUPROFEN") OR DE "NONSTEROIDAL anti-inflammatory agents" OR DE "LOCAL anesthesia" OR DE "CORTICOSTEROIDS" OR DE "DICLOFENAC" | 8262 |
| 5 | XB ('topical analgesi*' OR 'topical pain relie*' OR 'transdermal analgesi*' OR 'transdermal pain relie*' OR 'trans dermal analgesi*' OR 'trans dermal pain relie*' OR 'transcutaneous analgesi*' OR 'transcutaneous pain relie*' OR 'trans cutaneous analgesi*' OR 'trans cutaneous pain relie*') OR DE "OINTMENTS" OR XB ((Topical* OR transdermal* OR trans-dermal* OR patch* OR ointment* OR cream* OR "gel" OR "gels" OR Liniment*)) | 9556 |
| 6 | ((((S1 OR S2) AND S3) AND S4) AND S5 | 54 |
